# Unconditional quantile regressions to determine the social gradient of obesity in Spain 1993–2014

**DOI:** 10.1186/s12939-016-0454-1

**Published:** 2016-10-19

**Authors:** Alejandro Rodriguez-Caro, Laura Vallejo-Torres, Beatriz Lopez-Valcarcel

**Affiliations:** 1 Department of Quantitative Methods, Universidad de Las Palmas de Gran Canaria, Las Palmas de Gran Canaria, Spain; 2 UCL Department of Applied Health Research, UCL, University College London, Gower Street, London, WC1E 6BT UK

**Keywords:** Obesity, Social inequalities, Unconditional quantile regression

## Abstract

**Background:**

There is a well-documented social gradient in obesity in most developed countries. Many previous studies have conventionally categorised individuals according to their body mass index (BMI), focusing on those above a certain threshold and thus ignoring a large amount of the BMI distribution. Others have used linear BMI models, relying on mean effects that may mask substantial heterogeneity in the effects of socioeconomic variables across the population.

**Method:**

In this study, we measure the social gradient of the BMI distribution of the adult population in Spain over the past two decades (1993–2014), using unconditional quantile regressions. We use three socioeconomic variables (education, income and social class) and evaluate differences in the corresponding effects on different percentiles of the log-transformed BMI distribution. Quantile regression methods have the advantage of estimating the socioeconomic effect across the whole BMI distribution allowing for this potential heterogeneity.

**Results:**

The results showed a large and increasing social gradient in obesity in Spain, especially among females. There is, however, a large degree of heterogeneity in the socioeconomic effect across the BMI distribution, with patterns that vary according to the socioeconomic indicator under study. While the income and educational gradient is greater at the end of the BMI distribution, the main impact of social class is around the median BMI values. A steeper social gradient is observed with respect to educational level rather than household income or social class.

**Conclusion:**

The findings of this study emphasise the heterogeneous nature of the relationship between social factors and obesity across the BMI distribution as a whole. Quantile regression methods might provide a more suitable framework for exploring the complex socioeconomic gradient of obesity.

**Electronic supplementary material:**

The online version of this article (doi:10.1186/s12939-016-0454-1) contains supplementary material, which is available to authorized users.

## Background

Many economic and epidemiological studies have documented the increasing prevalence of obesity in adults in developed societies, as well as the presence of an important social gradient in this respect, especially among women, measured in terms of education, income and/or occupation-social class [1]. The mechanisms and processes underlying this gradient have been analysed in the framework of various theories, such as human capital, rational addiction, contagion, patterns and social standards of population subgroups. A WHO paper proposed the social determinants of health as a framework, and suggested that “the causes of the causes” of obesity should be analysed [2].

The weight-height ratio is usually measured by the Body Mass Index (BMI), defined as weight in kilograms divided by the square of height in metres. From this calculation, the following levels are defined: < 18.5 underweight; < 25 normal weight; < 30 overweight and > 30 obesity. Many research papers have categorised BMI and measured the gradient in terms of the relative likelihood of being obese (or overweight) according to whether the individual is a member of more or less privileged social categories. Working with a continuous BMI scale, however, enables more nuanced results to be obtained.

Traditional methods of measuring socioeconomic inequalities in obesity [3] take a single measure or estimate, referring to the average of the distribution (assuming, therefore, that the effect of education or income is the same for all individuals, all else being equal, regardless of body mass). Nevertheless, this may not be a realistic hypothesis. Becoming obese takes place over considerable time, and the BMI recorded today is the outcome of a lifelong, continuous and cumulative process. It is plausible, as we hypothesise in this paper and show empirically, that the impact of a socioeconomic variable on the BMI may not be homogeneous across the entire distribution of this index. In this case, determining a gradient by calculating averages (for a single parameter) is a simplification, which may reflect the reality in the vicinity of the median of the distribution, but not at its extremities (extremely thin or obese people). In other words, focusing on “mean effects” may mask substantial heterogeneity in the effects of socioeconomic variables across the population.

Conditional quantile regression (CQR) has been used in recent studies of the determinants of obesity to measure the impact of a covariate on a quantile of the BMI, conditional on specific values of other covariates [4–9]. For the most part, these are cross-sectional analyses with observational data, although some use longitudinal information. On the other way around, some studies estimate the effects of BMI on wages, and found heterogeneity along the distribution, which cannot be estimated with ordinary least squares (OLS) [10]. Censored CQR has also been used to assess the impact of fiscal policy (VAT increase) on the consumption of healthy/unhealthy food. The conclusion reached is that an increase in VAT “is more effective in reducing purchases of unhealthy foods among high-purchasing households than a VAT removal is in increasing the purchases of healthy foods among low-purchasing households” [11]. Virtually all published studies using QR have found that the effects are not homogeneous across the whole distribution of BMI and therefore that OLS is not the most appropriate method to represent the associations between obesity and its determinants.

Fewer studies have been conducted to monitor the evolution of the social gradient in obesity, and hardly any have dynamically compared the magnitudes of the impacts of different sources of social inequality, arising from various underlying mechanisms. Inequalities in the prevalence of obesity associated with education background are mainly due to differences in tastes (which in turn are related to the formation of preferences since childhood) and to economic restrictions on the capacity to consume a healthy diet (calorie-dense, high-energy foods are cheaper, and their price has tended to fall further due to prevailing trends in global and local markets), given the association between education and income. More highly educated people are more efficient producers of health [12] and better able to manage information, and thus have a greater ability to design good, healthy diets [1]. Education improves productive efficiency (better use of inputs for health) and allocative efficiency (more use of health inputs) [13]. Furthermore, household income and social class approximate the socioeconomic status of the family. Household income can impose economic restrictions on the diet consumed, and such limitations would affect the lower deciles in particular, and this process has been more intense in recent years because the structure of relative prices has made fresh food more costly than processed food [14]. Social class usually combines information about employment and about the education of the head of the household, and therefore this parameter tends to remain more stable over time than income. For a specific individual, household variables are less controllable than education. Most of the literature use measures of social class based solely on occupation. For example, the official measure in United Kingdom’s population census and population surveys is the ‘Registrar-General’s Social Classes’ introduced in 1913, and that was renamed in 1990 as ‘Social Class based on Occupation’. The standard definition and measure of social class in Spain is similar to that in the UK.

Each of these socioeconomic indicators captures different facets of the social gradient in obesity, and their comparison enables us to explore their role in greater detail.

In this study, we measure changes in the BMI distribution of the adult population in Spain over the past two decades (1993–2014). The main reason to choose this research topic is that obesity is a serious public health problem in Spain and its prevalence is increasing among adults. Around 17 % of persons older than 18 years are obese in Spain (53 % are overweight or obese). Besides that, the social gradient of obesity in Spain is substantial as shown by raw numbers: 5.3 % of women with higher education are obese, while 30 % of women with no primary studies are obese. Some studies have measured the social gradient of obesity in Spain, for example [15–17]. However, no previous work has analysed different sources of inequality in obesity in Spain allowing for a potential heterogeneous effect across the BMI distribution.

The main objective of this paper is thus to estimate the effects of three socioeconomic variables (education, income and social class) on the BMI, and to evaluate differences in the corresponding effects on different percentiles of the BMI distribution. Possible changes in these effects over time are also discussed. In contrast to most previous studies in this field, we use unconditional quantile regression (UQR) models [18]. Although CQR is employed more frequently, UQR is preferable in order to interpret the heterogeneity across the distribution of outcomes in a population and policy context (16), because CQR results might not be generalizable.

This approach enables us to compare the gradient attributable to different proxies of socioeconomic level, as the estimates can be interpreted as effects on the same (unconditional) distribution of BMI. Another distinctive facet is that we model the logarithm of BMI as a dependent variable, rather than using the simple BMI, which could amplify the heterogeneity of the effects in the distribution. Indeed, one BMI point represents only a small proportion of the body mass of a person with obesity but a substantial proportion of that of a person with low weight. In estimating relative or proportional changes, using logarithms, we re-scale the effects, thus avoiding such an amplification.

In summary, a fundamental contribution of the present study is that it enables us to compare the social gradient in obesity among the three alternative ways of measuring the socioeconomic status of individuals and households. Thus, we pose very flexible hypotheses about the distribution of the effects among the population, through the use of UQR. Moreover, the effects over time and according to gender can be compared. In addition, we model the relative changes in BMI, which ensures that the scale of the effect is comparable between quantiles, avoiding the risk of amplification of the effects due to a simple question of scale.

## Methods

### Data

This study uses independent cross-section databases from the Spanish National Health Survey (SNHS) 1993 (*n* = 19,504) and 2006 (*n* = 28,507) and the 2014 European Health Survey (sample from Spain, *n* = 21,877). The SNHS is an official survey conducted by the Ministry of Health, Social Services and Equality in collaboration with the National Institute of Statistics. It is designed to obtain information about the overall health of citizens, their degree of access to and use of health services, and the determinants of health, among other questions. To achieve these goals, our research considers all persons residing in main family dwellings, throughout Spain. Data were compiled over a period of 1 year, by three-stage stratified sampling.

The study population was aged 18 years or more. For 1993, the only socioeconomic variable was education, while for the other 2 years all three variables were available.

### Statistical methods

Unconditional quantile regression (UQR) models [19, 20] were used, with the logarithm of BMI as the dependent variable. All models were controlled for age, region of residence, marital status and employment status. Men and women were modelled separately. The models alternately measure socioeconomic status through education (4 levels), equivalent household income (Q10, Q25, Q50, Q75, Q90) and social class defined by the occupation of the head of household (six categories). The three measures are homogeneous among the different surveys considered.

Quantile regression has a fundamental advantage over least squares estimation in that it not only estimates the changes that occur around the mean of the endogenous variable, conditional on the values of the exogenous ones, but also the effects across the entire distribution of the endogenous variable. The least squares model produces a single coefficient for the effect of the cause variable (in the present case, for example, education) on the effect variable (BMI). Therefore, it assumes homogeneity throughout the BMI distribution, or that inference is performed locally around the mean BMI of the sample. In cross-sectional comparisons, it would be interpreted as the expected change in BMI, ceteris paribus, in a person with a low educational background who had an average BMI and who after schooling completed their education. If the coefficient was non-significant, we could conclude that education had no effect on mean BMI, but we would be unable to conclude anything about other points of the population distribution of BMI. Nevertheless, education can affect different individuals in different ways.

One way to model this individual unobservable heterogeneity is by assuming that heterogeneity is associated with a person’s weight (BMI), and by applying quantile regression. This approach generalises the estimation of a single coefficient and better illustrates the social gradient in obesity. For example, a background of higher education may provide greater protection against obesity for people who are already overweight, i.e. the education gradient would be steeper at the upper end of the BMI distribution than around the mean.

Unlike OLS, CQR estimates the effects at different points of the distribution of the endogenous variable, for example at the 5^th^, 25^th^, 50^th^ and 95^th^ percentiles. This tells us how the independent variable or cause affects the entire distribution of the dependent or effect variable, and not only its mean, always conditional to the exogenous values. The coefficients are interpreted in relation to the quantiles of the conditional distribution defined by the covariates, and therefore the different models are not comparable.

CQR estimation is based on minimising a function of mean absolute deviations:

1 $$ {\displaystyle {\sum}_{i:{y}_i\ge {x}_i\beta}^N\tau \left|{y}_i-{x}_i\beta \right|+{\displaystyle {\sum}_{i:{y}_i\le {x}_i\beta}^N\left(1-\tau \right)\left|{y}_i-{x}_i\beta \right|}} $$

where *y* is the dependent variable, *x* the explanatory variables, β the parameters to be estimated and τ the percentile to be obtained. Application of this technique reveals the effects of each covariate on the different percentiles of the dependent variable, conditional to the value of the other exogenous variables in the model. For the 50^th^ percentile, the estimator is called the minimum absolute deviation (MAD) and coincides with the OLS for the Laplacian (exponential two-tailed) distribution. The MAD estimator is traditionally used in econometrics as an estimator that is robust to non-normality and the presence of outliers [21].

UQR is based on extending the concept of Influence Function to what has been termed the Recent Influence Function (RIF) (4). This is defined as follows:

2 $$ RIF\left(y;{q}_{\tau}\right)={q}_{\tau }+\frac{\tau -I\left[y\le {q}_{\tau}\right]}{f_y\left({q}_{\tau}\right)} $$

where *q*_*τ*_ is the value of percentile *τ*, f_y_(q_τ_) is the sample density function in the sample percentile *τ*, and I is a dichotomous variable that takes the value one when the value of y is less than the corresponding percentile.

After recalculating the variables of interest, the following regression is then estimated by OLS:

3 $$ RIF\left(y;{q}_{\tau}\right)=X{\beta}^{UQR}+\varepsilon $$

Since the explanatory variables do not enter into the transformation of equation (2), although the X’s in the model change, the interpretation of the estimated effects does not vary, and so alternative models can be compared and different sources of socioeconomic inequality incorporated. The main advantage of this method over conditional regression is that the estimated effects do not depend on the set of explanatory variables in the model. Moreover, as in the conditional regression, the estimates are robust to outliers [22, 23].

In practice, the greatest difficulty encountered is that of estimating the density function of Y, which is usually done by nonparametric kernel estimators. Since these estimates may be sensitive to the choice of bandwidth, a sensitivity analysis should be performed previously. The results shown in the text are based on a Gaussian kernel with an optimal bandwidth calculated according to Silverman [24]. The standard errors were calculated using bootstrap with 400 replications.

### Variables

The dependent variable is the natural logarithm of self-reported BMI. The three variables of interest, which alternately measure the social gradient, are occupation/social class, household income and education.

Occupation/Social class: Social class refers to the occupation and if applicable the education background of the main provider of the household. The following survey categories and definitions were used:

- ○ Social class I - Owners and managers of establishments with 10 or more employees and professional staff with university degrees.
- ○ Social class II - Owners and managers of establishments with fewer than 10 employees, professional staff with college diplomas and other technical support staff. Sportspersons and artists.
- ○ Social class III – Intermediate occupations and self-employed persons.
- ○ Social class IV – Supervisors and skilled workers.
- ○ Social class V – Primary sector skilled workers and other semi-skilled workers.
- ○ Social class VI - Unskilled workers.

Household income: categorised from the percentiles of the equivalent household income, according to the weights established by the OECD^1^: 1 for the first adult, 0.5 for each other adult in the household and 0.3 for each child. In order to standardise the income data from the surveys considered, we defined five cut-off points or percentiles of equivalent household income for the year of the survey, at 10, 25, 50, 75 and 90 %, thus creating six intervals of income, which reflect the extreme values of the distribution, together with the median.

Education: four homogeneous levels of education are defined in the three surveys considered: unfinished primary, primary, secondary and university studies.

In addition, all models include the following covariates: age, in years, and its square; dummies for the Autonomous Community (region) of residence (17 in total, excluding those of Ceuta and Melilla, which are autonomous cities on the African mainland); marital status, with five categories: single, married, widowed, separated and divorced; employment status, with six categories: working, unemployed, retired, studying, housework and others.

To test for robustness, we estimated models for the BMI (rather than its logarithm). Moreover, since a key part of the method is to estimate the density function, which is determined by the kernel and the optimal bandwidth, we estimated models, as a sensitivity analysis, with different kernels and optimal bandwidth criteria [25]. Three kernels, Epanechnikov, Gaussian and Rectangular, were used alternately, together with three methods to choose the optimal bandwidth, Silverman [24], Härdle [26] and Scott [27]. Consequently, a total of 11,890 parameters were estimated, following the nine possibilities for optimal bandwidth and kernels.

## Results

### Description of the sample

The sample includes the 19,788, 28,507 and 21,877 adults recorded in the surveys of 1993, 2006 and 2014, respectively. As shown in Table 1, the biggest changes occurred in both sexes between 1993 and 2006. Up to the middle of the distribution, the BMI of the men is higher than that of the women, and the values converge close to the median. This equality then persists until the 95^th^ percentile, above which the women have a higher BMI.

**Table 1:** Percentiles of BMI by sex

Year	5 %	15 %	25 %	50 %	75 %	85 %	95 %
Women							
1993	18.9	20.3	21.3	23.7	26.7	28.4	32.0
2006	19.2	20.8	22.0	24.8	28.0	30.1	34.1
2014	19.1	20.7	22.0	24.7	28.2	30.5	34.4
Men							
1993	20.6	22.3	23.4	25.31	27.5	28.8	31.2
2006	21.1	22.9	24.0	26.2	28.7	30.3	33.5
2014	21.1	22.9	24.0	26.1	28.8	30.5	33.8

The prevalence of overweight increased between 1993 and 2006, and then remained stable until 2014. The increase in both sexes was 10 %, and the value for men remained 15 percentage points above that for women. However, the percentage of persons with obesity was very similar in both sexes, with an increase of around 6–7 points from 1993 to 2006.

**Fig. 1:**
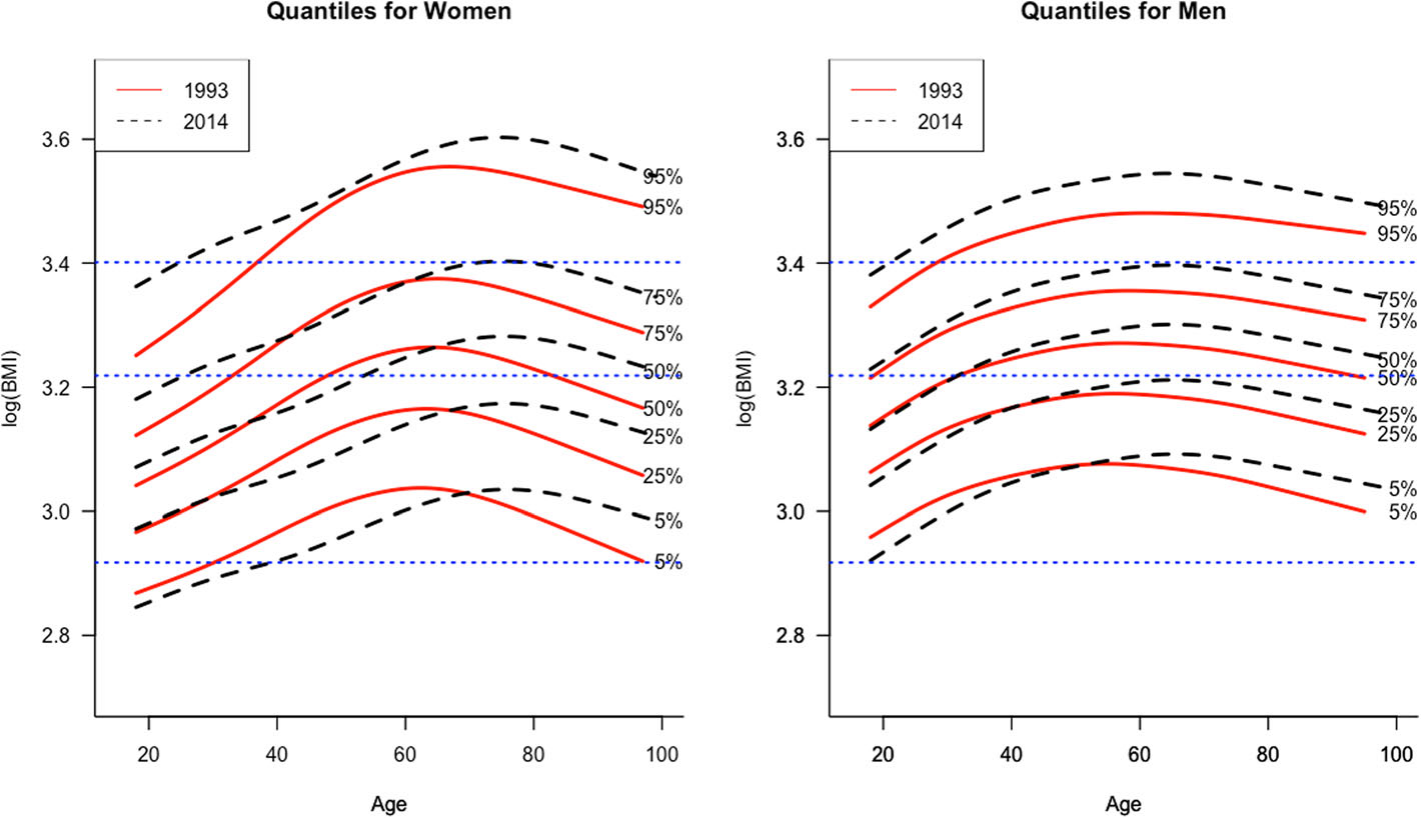
BMI percentiles by sex and age

Figure 1 compares the BMI distribution in the initial and final years of the study (1993–2014), showing the 5^th^, 25^th^, 50^th^, 75^th^ and 95^th^ percentiles, by age, for men and women. The lines of points parallel to the X axis mark the four BMI zones (underweight, normal, overweight and obese).

The population distribution of BMI shifted to the right from 1993 to 2014 for men and women (median BMI in 1993 was 23.7 for women and 25.3 for men; in 2014 it was 24.7 and 26.1, respectively). By age, there is a gender difference: the BMI for men worsened at almost all ages, while for women of middle age (40–65 years) close to the median, the BMI improved.

The male population has gained weight in the last 21 years, as shown by the fact that BMI values increased in all ages and percentiles except among those younger than 35–40 years and with BMI below the median. Among women, the 95^th^ is the percentile of greatest weight for all ages, but for the other percentiles, the weight was lower in 2014 for persons of middle age and those aged over 60 years, approximately. The prevalence of underweight was higher in 2014 than in 1993 for young women and those aged up to 40 years.

**Fig. 2:**
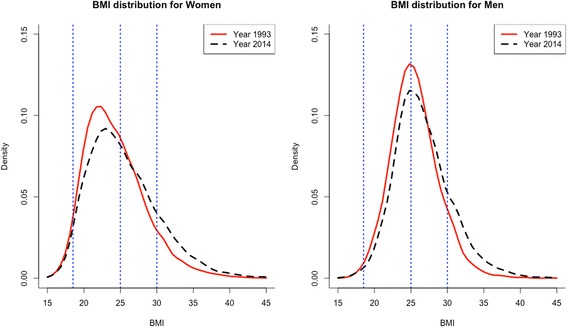
Density estimations for BMI by sex

Figure 2 presents the estimates by sex of the BMI sampling densities, for 1993 and 2014. The rightward shift of the BMI distribution over these two decades, especially for men, is confirmed.

Table 2 contains the univariate descriptives of the sample for the 3 years.

**Table 2:** Descriptive statistics

Variable	Category	1993	2006	2014
Social Class (*n* and % in each category)	I	7293 (36.9 %)	2532 (8.9 %)	2376 (10.9 %)
	II	306 (1.5 %)	2739 (9.6 %)	1776 (8.1 %)
	III	876 (4.4 %)	7188 (25.2 %)	4038 (18.5 %)
	IV	2847 (14.4 %)	7766 (27.2 %)	3199 (14.6 %)
	V	5694 (28.8 %)	3816 (13.4 %)	7029 (32.1 %)
	VI	2540 (12.8 %)	3846 (13.5 %)	2952 (13.5 %)
	Missing	232 (1.2 %)	620 (2.2 %)	507 (2.3 %)
Equivalent family income (euros/month) (mean and standard deviation)	Percentile 10		273.45 (71.6)	287.23 (48.7)
	Percentile 10–25		467.39 (42.2)	480.36 (20.5)
	Percentile 25–50		640.96 (70)	711.69 (93.3)
	Percentile 50–75		902.22 (130)	1081.57 (130.8)
	Percentile 75–90		1368.12 (117.7)	1618.53 (188)
	Percentile 90–100		2192.99 (641.2)	2938.84 (765.6)
Education (*n* and % in each category)	No education qualifications or primary studies incomplete	3175 (16 %)	3968 (13.9 %)	2832 (12.9 %)
	Primary	9835 (49.7 %)	9909 (34.8 %)	4944 (22.6 %)
	Secondary	4947 (25 %)	10,109 (35.5 %)	9931 (45.4 %)
	University	1696 (8.6 %)	4385 (15.4 %)	4170 (19.1 %)
	Missing	135 (0.7 %)	136 (0.5 %)	0 (0 %)
Age (mean and standard deviation)		44.88 (17.9)	51.19 (18.2)	53.29 (18.1)
Marital status (*n* and % in each category)	Single	5270 (26.6 %)	6867 (24.1 %)	5336 (24.4 %)
	Married	12,581 (63.6 %)	16,430 (57.6 %)	12,081 (55.2 %)
	Separated	264 (1.3 %)	868 (3 %)	545 (2.5 %)
	Divorced	100 (0.5 %)	679 (2.4 %)	1006 (4.6 %)
	Widowed	1512 (7.6 %)	3598 (12.6 %)	2887 (13.2 %)
	Missing	61 (0.3 %)	65 (0.2 %)	22 (0.1 %)
Employment situation (*n* and % in each category)	Working	8177 (41.3 %)	13,375 (46.9 %)	7958 (36.4 %)
	Retired	3626 (18.3 %)	7930 (27.8 %)	6447 (29.5 %)
	Unemployed	1789 (9 %)	1747 (6.1 %)	2476 (11.3 %)
	Studying	1437 (7.3 %)	731 (2.6 %)	2393 (10.9 %)
	Housework	4621 (23.4 %)	4373 (15.3 %)	1613 (7.4 %)
	Other	82 (0.4 %)	289 (1 %)	990 (4.5 %)
	Missing	56 (0.3 %)	62 (0.2 %)	0 (0 %)

### Quantile regression. Results of the estimations

**Fig. 3:**
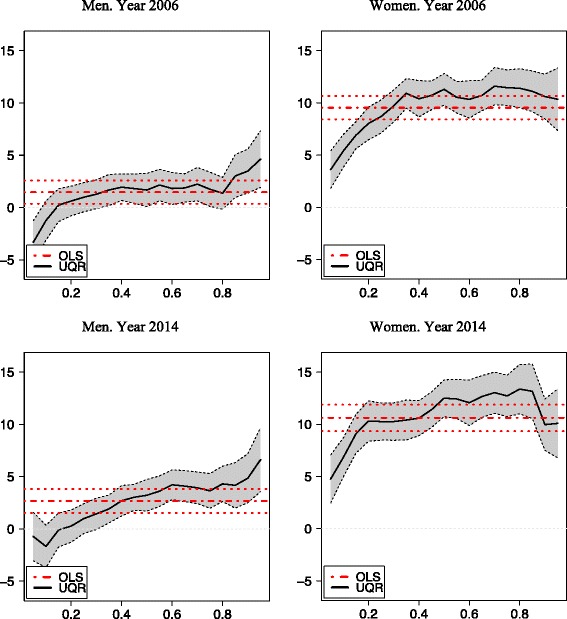
OLS and UQR estimations. Social Class VI vs Social Class I

**Fig. 4:**
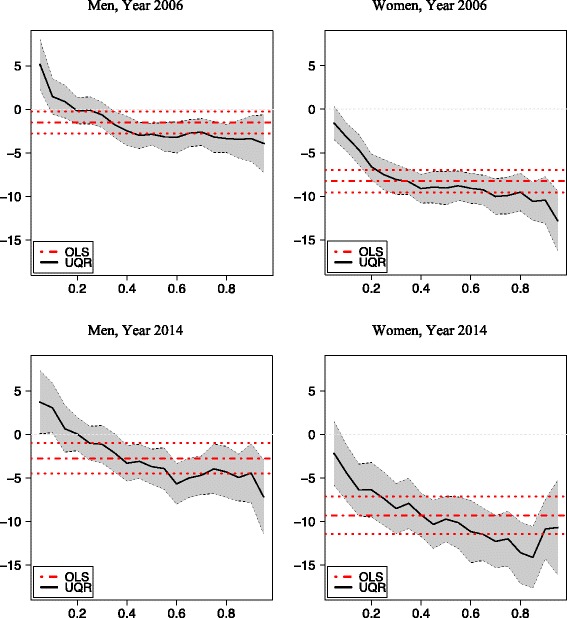
OLS and UQR estimations. Higher Income vs lower Income intervals

**Fig. 5:**
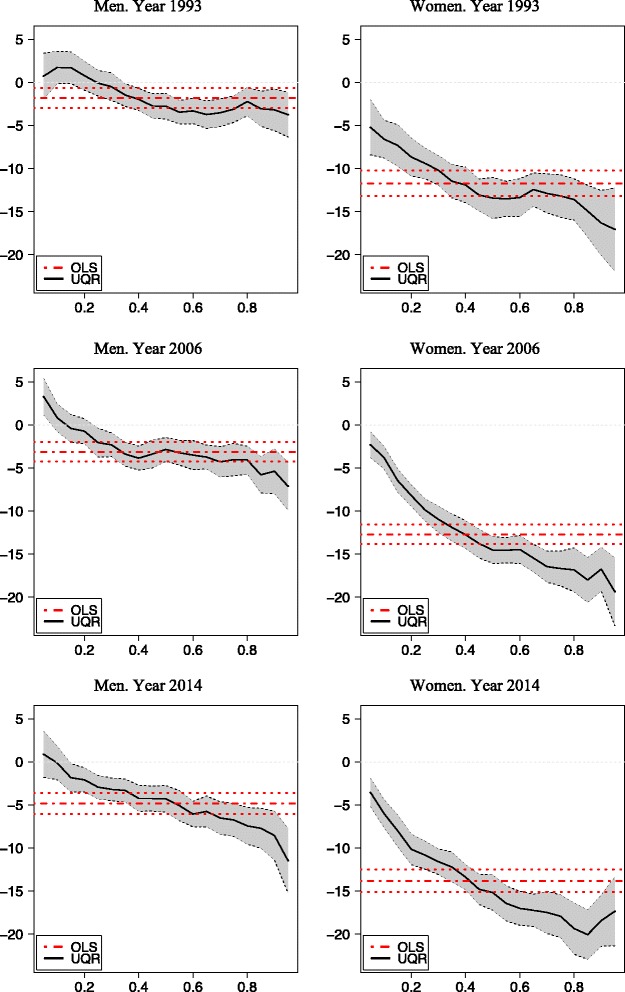
OLS and UQR estimations. University education vs No studies or unfinished primary studies

Table 3 contains the estimations for each year of the OLS coefficients (and their standard errors) and of the UQR for five selected quantiles (5, 25, 50, 75 and 95) for men and women. Figures 3, 4 and 5 represent the coefficients and the corresponding 95 % confidence intervals for the extreme values of the social class, income and education categories, respectively, for men and women.

**Table 3:** OLS and UQR estimations. Dependent variable log(BMI)

	OLS		Percentile 5		Percentile 25		Median		Percentile 75		Percentile 95	
	Male	Female	Male	Female	Male	Female	Male	Female	Male	Female	Male	Female
1993												
Primary Edu	0.007	−0.038^a^	0.007	0.000	0.016^a^	−0.001	0.008	−0.031^a^	0.009	−0.063^a^	−0.006	−0.128^a^
Secondary Edu	−0.011^b^	−0.083^a^	−0.001	−0.018^b^	0.001	−0.052^a^	−0.014^b^	−0.093^a^	−0.015^b^	−0.109^a^	−0.02^b^	−0.161^a^
University Edu	−0.018^a^	−0.117^a^	0.007	−0.052^a^	−0.001	−0.094^a^	−0.028^a^	−0.134^a^	−0.031^a^	−0.132^a^	−0.037^a^	−0.171^a^
2006												
Income 25	0.005	−0.008	0.017	0.003	0.007	−0.007	−0.002	−0.006	0.001	−0.013	0.003	−0.04^b^
Income 50	0.006	−0.024^a^	0.035^a^	−0.007	0.009	−0.012^b^	0.004	−0.017^b^	0.000	−0.03^a^	−0.002	−0.069^a^
Income 75	−0.001	−0.037^a^	0.037^a^	−0.005	0.008	−0.027^a^	−0.005	−0.034^a^	−0.011	−0.052^a^	−0.026	−0.076^a^
Income 90	−0.006	−0.066^a^	0.054^a^	−0.018−	0.005	−0.059^a^	−0.012^b^	−0.071^a^	−0.018^b^	−0.087^a^	−0.045^a^	−0.112^a^
Income 100	−0.015^b^	−0.083^a^	0.052^a^	−0.016	−0.001	−0.075^a^	−0.029^a^	−0.091^a^	−0.032^a^	−0.099^a^	−0.039^b^	−0.128^a^
Social Class II	0.000	0.026^a^	−0.007	0.012	0.000	0.022^a^	0.002	0.039^a^	−0.005	0.033^a^	0.008	0.002
Social Class III	0.015^a^	0.051^a^	−0.001	0.026^a^	0.014^b^	0.057^a^	0.019^a^	0.063^a^	0.015^a^	0.06^a^	0.03^b^	0.033^a^
Social Class IV	0.015^a^	0.083^a^	−0.02^b^	0.039^a^	0.011	0.081^a^	0.022^a^	0.105^a^	0.021^a^	0.097^a^	0.022−	0.076^a^
Social Class V	0.019^a^	0.077^a^	−0.019-	0.034^a^	0.019^a^	0.08^a^	0.029^a^	0.1^a^	0.023^a^	0.087^a^	0.042^a^	0.062^a^
Social Class VI	0.015^a^	0.095^a^	−0.033^a^	0.036^a^	0.010	0.087^a^	0.017^b^	0.113^a^	0.017^b^	0.115^a^	0.046^a^	0.103^a^
Primary Edu	−0.003	−0.039^a^	0.008	0.002	−0.003	−0.005	0.005	−0.032^a^	0.001	−0.062^a^	−0.017	−0.117^a^
Secondary Edu	−0.014^b^	−0.081^a^	0.010	−0.004	−0.006	−0.043^a^	−0.006	−0.088^a^	−0.016^b^	−0.117^a^	−0.041^a^	−0.16^a^
University Edu	−0.031^a^	−0.127^a^	.033^a^	−0.023^a^	−0.021^b^	−0.098^a^	−0.028^a^	−0.145^a^	−0.04^a^	−0.167^a^	−0.071^a^	−0.194^a^
2014												
Income 25	−0.02^a^	−0.014^b^	0.000	0.005	−0.017^b^	0.005	−0.016	−0.003	−0.022^b^	−0.034^b^	−0.051^b^	−0.046^b^
Income 50	−0.008	−0.005	0.014	−0.005	0.004	0.005	−0.004	0.005	−0.018-	−0.003	−0.049^b^	−0.033
Income 75	−0.009	−0.036^a^	0.023	0.003	−0.005	−0.004	−0.008	−0.041^a^	−0.012	−0.056^a^	−0.051^a^	−0.071^a^
Income 90	−0.026^a^	−0.061^a^	0.013	−0.017	−0.013	−0.034^a^	−0.026^a^	−0.061^a^	−0.041^a^	−0.088^a^	−0.069^a^	−0.089^a^
Income 100	−0.028^a^	−0.093^a^	0.037^b^	−0.022	−0.010	−0.074^a^	−0.037^a^	−0.097^a^	−0.04^a^	−0.12^a^	−0.072^a^	−0.107^a^
Social Class II	0.005	0.023^a^	0.003	0.017	−0.003	0.031^a^	0.009	0.034^a^	0.012	0.025^a^	0.024−	−0.005
Social Class III	0.015^a^	0.046^a^	0.004	0.023^b^	0.008	0.051^a^	0.015^b^	0.051^a^	0.019^a^	0.063^a^	0.055^a^	0.032^a^
Social Class IV	0.028^a^	0.082^a^	0.006	0.044^a^	0.02^a^	0.078^a^	0.031^a^	0.1^a^	0.038^a^	0.097^a^	0.038^a^	0.074^a^
Social Class V	0.034^a^	0.088^a^	0.003	0.037^a^	0.018^a^	0.085^a^	0.036^a^	0.104^a^	0.045^a^	0.103^a^	0.072^a^	0.081^a^
Social Class VI	0.027^a^	0.106^a^	−0.007	0.048^a^	0.010	0.103^a^	0.032^a^	0.125^a^	0.036^a^	0.127^a^	0.066^a^	0.101^a^
Primary Edu	−0.008	−0.031^a^	−0.010	0.002	0.000	−0.013^a^	−0.004	−0.023^a^	−0.005	−0.052^a^	−0.04^b^	−0.072^a^
Secondary Edu	−0.019^a^	−0.083^a^	0.003	−0.014^b^	−0.003	−0.047^a^	−0.011	−0.08^a^	−0.029^a^	−0.12^a^	−0.061^a^	−0.13^a^
University Edu	−0.048^a^	−0.138^a^	0.009	−0.035^a^	−0.029^a^	−0.108^a^	−0.043^a^	−0.152^a^	−0.067^a^	−0.179^a^	−0.115^a^	−0.173^a^

a: 1 % significance; b: 5 % significance; c: 10 % significance

The gradient of obesity is clearly apparent, with the three socioeconomic variables, both with OLS and with UQR. The social gradient is steeper for women than for men. When the reference category is one extreme of the distribution (the lowest levels of education and income, and the highest of social class), the coefficients estimated by OLS have the expected sign and monotonic function, increasing in absolute value; with few exceptions, these coefficients are always significant. However, in most cases, OLS does not properly represent a heterogeneous reality, which on the other hand is reflected in the UQR estimates, with stronger impacts at the upper end of the BMI distribution, i.e. for the persons with obesity in some cases, and an inverted U-shaped profile (i.e., with stronger impacts in the proximity of the medium and lesser weights at both extremes) in others.

### Social class

The gradient for social class is very different for men and women, being notably steeper for the latter, and this difference was greater in 2014 than in 2006. For men, the differences in obesity by social classes are very small or non-significant, and OLS in general provides an accurate reflection of the impacts across the BMI distribution. However, for women there are very significant differences among social classes, in both years, with greater differences in 2014 than in 2006, and the effects are heterogeneous according to the BMI distribution, with an inverted U shape. By OLS, between a woman of class I and another of class VI there is expected to be a difference of 9.5 % in BMI in 2006 and 10.6 % in 2014. But those differences increase to 10.5 and 11.7 % respectively around the 75^th^ percentile, and decrease to 3.6 and 4.8 % for the 5^th^ percentile. The major change in this respect occurs between classes III and IV, especially for women.

### Income

With respect to the gradient of obesity according to household income, this too is more intense for women. By OLS, in 2006 women with the highest levels of income had a BMI that was 8 % lower than that of women with the lowest income (among men, the corresponding difference was only 2 %). As with social class, this gradient became steeper in 2014, but only slightly so and not homogeneously. By UQR estimation, there were no significant differences among the top three income brackets. The major change takes place in the fourth bracket, and from this point there is a clear difference in favour of wealthier women. This difference was greater than among men, and also greater among persons with overweight or obesity. In 2006, the maximum difference was recorded around the 95^th^ percentile (the BMI was 13 % lower in women from the wealthiest households than those from the poorest ones). In 2014, the maximum difference (12 %) occurred around the 75^th^ percentile. However, the variations between 2006 and 2014 were not very large.

### Education

Of the three sources of social inequality in obesity considered in this study, education is the most important and the one expressing the greatest difference between the sexes, being considerably more intense for women. It is also the most heterogeneous source of inequality, across the entire distribution of BMI. Therefore, OLS does not accurately represent the educational gradient in obesity in Spain. For the 1993, 2006 and 2014 models, the OLS gradient indicates a continuous but moderate increase in the education gradient. Thus, the difference in BMI between a university-educated woman and one with no formal education was 12 % in 1993, 13 % in 2006 and 14 % in 2014. For men, too, a similar progressive worsening was observed, although the gradient was much less steep (2, 3 and 5 % in the three respective years). The protective effect of university education seems to have intensified over time, but the effect of primary school studies compared with no studies remained unchanged.

Education has markedly heterogeneous effects across the distribution of BMI, but particularly at the extremes. UQR gives results that differ significantly from those obtained by OLS in these intervals. For example, for women in the 5^th^ percentile in 2006, the difference in BMI between those with a university education and those with no formal education was 2 %, while for those in the 95^th^ percentile it was 19 %. Among men, there is some evidence of a positive gradient for those with underweight (in 2006, University graduates in the 5^th^ percentile had a higher BMI than persons without qualifications), while the gradient was negative in the remaining percentiles. The latter effect, which is only apparent with the QR analysis, is indicative of a protective effect of higher education against underweight.

The dynamics of the education gradient differ among the BMI percentiles. For the lower ones (thin women), the gradient between university graduates and those with no qualifications steepened in the 1990s and early 2000s but remained stable or even decreased in more recent years. Among women with overweight or obesity, around the 75^th^ percentile, the gradient decreased slightly. At the extreme points of the distribution, although the gradient increased between 1993 and 2006, it fell slightly between 2006 and 2014.

### Tests of robustness

Our estimation of the linear models (for BMI rather than its logarithm) revealed similar patterns to those of the log-linear ones, although some coefficients were significant in the former but not in the latter. Detailed results of the linear models are shown in the Additional file 1.

A sensitivity analysis was performed with nine combinations of kernel and optimal bandwidth, based on the three estimates for each kernel and the corresponding optimal bandwidths. The mean difference between the estimates did not exceed 1 % for the Epanechnikov and Gaussian kernels, while the Rectangular kernel was more variable, with a mean of around 6 %. The Gaussian kernel (which the routine uses by default) was usually situated between the other two kernels used.

Average differences between the optimal bandwidth values were around 3 % for the three kernels proposed (0.7 % excluding the Rectangular kernel, which presented the greatest variability). The default option used [24] provided intermediate results between the other two.

In summary, the models are robust to alternative specifications and estimation methods. The detailed results of the sensitivity analysis are shown in the Additional file 1.

## Discussion

The social gradient of obesity has different dimensions. As Geyer et al., 2006 concluded: “*education, income, and occupational class cannot be used interchangeably as indicators of a hypothetical latent social dimension. Although correlated, they measure different phenomena and tap into different causal mechanisms” *[28]. Corroborating previous studies, we observed a significant social gradient in obesity in Spain. This social gradient has remained stable or increased during the last two decades, and is heterogeneous across the BMI distribution of the population. Therefore, an important conclusion to be drawn is that using OLS to model the socioeconomic gradient in BMI may mask socioeconomic differentiated effects of the variables, especially at the socioeconomic ends of the distribution. Previous studies that have employed OLS models on BMI have relied on a mean effect, while others that have focused on particular parts of the BMI distribution, e.g. exclusively on persons categorised as obese, have inevitably ignored a large part of the information on the distribution of BMI.

The advantage of using UQR instead of the more usual method, CQR, is that it enables us to compare the magnitude of the effects of alternative measures of socioeconomic status, and the coefficients estimated for a given BMI percentile can be interpreted directly as differences in the percentage of BMI within the same population. Our study compares the associations of obesity with two socioeconomic variables for the household (income and social class) and one for the individual (education). Using one or the other as the basis for measuring the social gradient in obesity and its evolution over time can lead to very different consequences.

In Spain, education is the main source of social inequality in obesity, and the one presenting greatest differences between men and women. In comparison with those with less education, women with university studies and in the 75^th^ percentile had approximately 18 % lower BMI in 2014. Our analysis found that the educational gradient in women doubles the gradient in men, and that although it worsened from 1993 to the mid 2000, it has remained rather stable since then. Education policies might be directed to the underlying cause, i.e. the educational gradient, by monitoring cases of girls dropping out of school in early ages. In Spain nowadays more women than men attain a university degree, but dropping out rates in primary and secondary school are higher than in most developed countries.

Between the richest and the poorest in the same percentile, the difference was 12 %, and between two women in the highest and lowest social classes, in the same percentile of BMI, the difference was roughly the same (12.7 %). The difference in the effects among social indicators is even more pronounced in the 95^th^ percentile. Corroborating other studies, we found that income and education have a stronger impact on the upper tail of the unconditional distribution of BMI, i.e., people with obesity. In contrast, we found that the maximum impact of social class was measured at intermediate levels of obesity. This finding, that the gradient for social class is less steep at the extremes, has not been previously reported in the literature.

One of the strengths of this paper is that we present logarithmic models of BMI, to rescale the changes in relative terms, thus reflecting the fact that the gain of a single BMI point by a person with obesity (BMI > 30) is not the same as that by a person with normal weight (18.5 < BMI < 25). By working with semilogarithmic models, we avoided overestimating effect differences within the range of BMI values. Nevertheless, the robustness tests showed that the sign of the results obtained does not change if a linear model of BMI is used.

Our results are in accordance with the findings of several previous studies. In Italy, the effect of education on BMI is found to be amplified when heterogeneity is incorporated into the distribution of BMI, using discontinuous regression methods [13]. In the same country it has been found that the effect of education is much more pronounced for persons with overweight and obesity. Our results are also similar to those presented in a study in Canada [29] in that the education gradient is steeper for high levels of BMI, and this gradient has not improved in recent decades. One of the few studies to employ UQR to measure the income gradient in obesity concluded that in the USA [30]. In Spain, no consistent positive gradient was observed in the area of underweight, which may reflect nutritional problems associated with extreme poverty. The only positive, significant effect was found among men with university studies versus those with no education qualifications in the 5^th^ percentile of BMI in 2006, which tends to support the latter hypothesis.

The causal channels between socioeconomic status and health are two-way [31] and so a limitation of our study is that it may be subject to endogeneity bias. As no instruments were found for education and the other socioeconomic variables, and as the cross-sectional data were independent, we cannot state unequivocally that we actually measured causal relationships. Nevertheless, different tests showed that our association results were very robust.

The results of this study show that neither the social gradient nor the gender gap are decreasing. In terms of policies, education stands up as the field where most needs to be done to offer a long-term approach to the problem. Furthermore, the evidence of a social gradient for income and social class suggests that poverty is a major risk factor for obesity, which provides an additional argument for income support and anti-poverty policies.

## Conclusion

Education is the most important source of social gradient in obesity among women in Spain, and this gradient is not decreasing. Inequalities among social classes and among income levels are roughly comparable, but with different patterns of heterogeneity within the distribution of BMI. The use of OLS to measure the social gradient in obesity may not be suitable because this method masks differences in the effects for varying degrees of obesity or overweight. UQR is preferable to CQR, although the latter is more commonly employed. We use UQR because it is easier to interpret, estimating the effects in the BMI quantiles across the entire population and not merely among certain population subgroups defined by the exogenous control variables. In addition, unconditional regression allows us to compare models with different explanatory covariates, which is the aim of this study.

## Additional file

Additional file 1: Unconditional Quantile Regression. BMI and Log (BMI). Sensibility analysis by kernel and bandwidth. (DOCX 306 kb)

## Abbreviations

BMI: Body mass index
CQR: Conditional quantile regression
OLS: Ordinary least squares
RIF: Recentered influence function
UQR: Unconditional quantile regression

## Footnotes

1
http://www.oecd.org/eco/growth/OECD-Note-EquivalenceScales.pdf
