# Supplementary material for: Unconditional quantile regressions to determine the social gradient of obesity in Spain 1993–2014
Source: Int J Equity Health. 2016 Oct 19;15:175. doi: 10.1186/s12939-016-0454-1 (PMC5070139; doi:10.1186/s12939-016-0454-1)
Supplement: Additional file 1: — Unconditional Quantile Regression. BMI and Log (BMI). Sensibility analysis by kernel and bandwidth. (DOCX 306 kb) [file 12939_2016_454_MOESM1_ESM.docx]

Unconditional Quantile Regression. BMI and Log(BMI)

|  | Men | | Women | |
| --- | --- | --- | --- | --- |
| Percentil | BMI | log(BMI) | BMI | log(BMI) |
| 1993 | | | | |
| Primary Education | | | | |
| 0.05 | 0.138 | 0.007 | 0.005 | 0 |
| 0.1 | 0.191 | 0.009 | 0.016 | 0.001 |
| 0.15 | 0.375^b^ | 0.017^b^ | 0.04 | 0.002 |
| 0.2 | 0.343^b^ | 0.015^b^ | -0.008 | 0 |
| 0.25 | 0.366^a^ | 0.016^a^ | -0.031 | -0.001 |
| 0.3 | 0.357^a^ | 0.015^b^ | -0.088 | -0.004 |
| 0.35 | 0.243^c^ | 0.01^c^ | -0.196c | -0.009c |
| 0.4 | 0.257^b^ | 0.01^c^ | -0.271b | -0.012c |
| 0.45 | 0.249^c^ | 0.01^c^ | -0.474^a^ | -0.02^a^ |
| 0.5 | 0.197 | 0.008 | -0.722^a^ | -0.031^a^ |
| 0.55 | 0.103 | 0.004 | -0.826^a^ | -0.034^a^ |
| 0.6 | 0.021 | 0.001 | -1.025^a^ | -0.041^a^ |
| 0.65 | 0.067 | 0.003 | -1.132^a^ | -0.045^a^ |
| 0.7 | 0.109 | 0.004 | -1.438^a^ | -0.055^a^ |
| 0.75 | 0.237 | 0.009 | -1.675^a^ | -0.063^a^ |
| 0.8 | 0.397^c^ | 0.014^c^ | -2.041^a^ | -0.075^a^ |
| 0.85 | 0.171 | 0.006 | -2.43^a^ | -0.085^a^ |
| 0.9 | -0.018 | -0.001 | -3.089^a^ | -0.103^a^ |
| 0.95 | -0.181 | -0.006 | -4.242^a^ | -0.128^a^ |
| Secondary | | | | |
| 0.05 | -0.013 | -0.001 | -0.334b | -0.018b |
| 0.1 | 0.14 | 0.006 | -0.55^a^ | -0.027^a^ |
| 0.15 | 0.202 | 0.009 | -0.658^a^ | -0.032^a^ |
| 0.2 | 0.089 | 0.004 | -0.924^a^ | -0.044^a^ |
| 0.25 | 0.029 | 0.001 | -1.11^a^ | -0.052^a^ |
| 0.3 | 0.013 | 0.001 | -1.339^a^ | -0.061^a^ |
| 0.35 | -0.222 | -0.009 | -1.428^a^ | -0.064^a^ |
| 0.4 | -0.216 | -0.009 | -1.573^a^ | -0.069^a^ |
| 0.45 | -0.276^c^ | -0.011^c^ | -1.9^a^ | -0.082^a^ |
| 0.5 | -0.345^b^ | -0.014^c^ | -2.199^a^ | -0.093^a^ |
| 0.55 | -0.51^a^ | -0.02^a^ | -2.317^a^ | -0.096^a^ |
| 0.6 | -0.554^a^ | -0.021^a^ | -2.512^a^ | -0.101^a^ |
| 0.65 | -0.579^a^ | -0.022^a^ | -2.507^a^ | -0.099^a^ |
| 0.7 | -0.472^b^ | -0.017^b^ | -2.686^a^ | -0.103^a^ |
| 0.75 | -0.409^b^ | -0.015^b^ | -2.884^a^ | -0.109^a^ |
| 0.8 | -0.204 | -0.007 | -3.055^a^ | -0.113^a^ |
| 0.85 | -0.435^c^ | -0.015^c^ | -3.617^a^ | -0.127^a^ |
| 0.9 | -0.571^c^ | -0.019^c^ | -4.279^a^ | -0.143^a^ |
| 0.95 | -0.602 | -0.02^c^ | -5.34^a^ | -0.161^a^ |
| Universitaryersitary | | | | |
| 0.05 | 0.153 | 0.007 | -0.981^a^ | -0.052^a^ |
| 0.1 | 0.378^c^ | 0.017^c^ | -1.322^a^ | -0.066^a^ |
| 0.15 | 0.384^c^ | 0.017^c^ | -1.509^a^ | -0.073^a^ |
| 0.2 | 0.18 | 0.008 | -1.822^a^ | -0.087^a^ |
| 0.25 | -0.019 | -0.001 | -2.007^a^ | -0.094^a^ |
| 0.3 | -0.119 | -0.005 | -2.224^a^ | -0.102^a^ |
| 0.35 | -0.352^b^ | -0.015^b^ | -2.559^a^ | -0.115^a^ |
| 0.4 | -0.484^a^ | -0.02^a^ | -2.706^a^ | -0.119^a^ |
| 0.45 | -0.678^a^ | -0.027^a^ | -3.026^a^ | -0.131^a^ |
| 0.5 | -0.699^a^ | -0.028^a^ | -3.17^a^ | -0.134^a^ |
| 0.55 | -0.885^a^ | -0.035^a^ | -3.265^a^ | -0.135^a^ |
| 0.6 | -0.859^a^ | -0.033^a^ | -3.312^a^ | -0.134^a^ |
| 0.65 | -0.986^a^ | -0.037^a^ | -3.146^a^ | -0.124^a^ |
| 0.7 | -0.95^a^ | -0.035^a^ | -3.345^a^ | -0.129^a^ |
| 0.75 | -0.847^a^ | -0.031^a^ | -3.497^a^ | -0.132^a^ |
| 0.8 | -0.62^b^ | -0.022^a^ | -3.689^a^ | -0.136^a^ |
| 0.85 | -0.883^a^ | -0.031^a^ | -4.252^a^ | -0.149^a^ |
| 0.9 | -0.943^a^ | -0.032^b^ | -4.883^a^ | -0.163^a^ |
| 0.95 | -1.126^a^ | -0.037^a^ | -5.642^a^ | -0.171^a^ |
| 2006 | | | | |
| Income 100 | | | | |
| 0.05 | 1.092^a^ | 0.052^a^ | -0.307 | -0.016 |
| 0.1 | 0.327 | 0.015 | -0.655^a^ | -0.032^a^ |
| 0.15 | 0.203 | 0.009 | -0.975^a^ | -0.047^a^ |
| 0.2 | -0.042 | -0.002 | -1.419^a^ | -0.066^a^ |
| 0.25 | -0.023 | -0.001 | -1.661^a^ | -0.075^a^ |
| 0.3 | -0.158 | -0.006 | -1.823^a^ | -0.081^a^ |
| 0.35 | -0.438^b^ | -0.018^b^ | -1.92^a^ | -0.083^a^ |
| 0.4 | -0.624^a^ | -0.025^a^ | -2.157^a^ | -0.091^a^ |
| 0.45 | -0.772^a^ | -0.03^a^ | -2.162^a^ | -0.089^a^ |
| 0.5 | -0.747^a^ | -0.029^a^ | -2.242^a^ | -0.091^a^ |
| 0.55 | -0.837^a^ | -0.031^a^ | -2.222^a^ | -0.088^a^ |
| 0.6 | -0.872^a^ | -0.032^a^ | -2.337^a^ | -0.091^a^ |
| 0.65 | -0.752^a^ | -0.027^a^ | -2.444^a^ | -0.093^a^ |
| 0.7 | -0.729^a^ | -0.026^a^ | -2.717^a^ | -0.1^a^ |
| 0.75 | -0.914^a^ | -0.032^a^ | -2.78^a^ | -0.099^a^ |
| 0.8 | -0.973^a^ | -0.034^a^ | -2.749^a^ | -0.095^a^ |
| 0.85 | -1.052^a^ | -0.034^a^ | -3.194^a^ | -0.106^a^ |
| 0.9 | -1.055^a^ | -0.034^b^ | -3.235^a^ | -0.104^a^ |
| 0.95 | -1.313^b^ | -0.039^b^ | -4.439^a^ | -0.128^a^ |
| Income 25 | | | | |
| 0.05 | 0.363 | 0.017 | 0.053 | 0.003 |
| 0.1 | 0.191 | 0.009 | -0.095 | -0.005 |
| 0.15 | 0.341^c^ | 0.015^b^ | -0.027 | -0.001 |
| 0.2 | 0.156 | 0.007 | -0.235c | -0.011 |
| 0.25 | 0.164 | 0.007 | -0.146 | -0.007 |
| 0.3 | 0.196 | 0.008 | -0.059 | -0.003 |
| 0.35 | 0.135 | 0.005 | 0.041 | 0.002 |
| 0.4 | 0.046 | 0.002 | -0.041 | -0.002 |
| 0.45 | -0.102 | -0.004 | 0.013 | 0.001 |
| 0.5 | -0.044 | -0.002 | -0.154 | -0.006 |
| 0.55 | -0.04 | -0.001 | -0.108 | -0.004 |
| 0.6 | -0.024 | -0.001 | -0.197 | -0.008 |
| 0.65 | 0.09 | 0.003 | -0.156 | -0.006 |
| 0.7 | 0.074 | 0.003 | -0.402 | -0.015 |
| 0.75 | 0.027 | 0.001 | -0.376 | -0.013 |
| 0.8 | -0.129 | -0.004 | -0.352 | -0.012 |
| 0.85 | 0.086 | 0.003 | -0.468 | -0.016 |
| 0.9 | 0.156 | 0.005 | -0.74c | -0.024c |
| 0.95 | 0.116 | 0.003 | -1.373b | -0.04b |
| Income 50 | | | | |
| 0.05 | 0.735^a^ | 0.035^a^ | -0.142 | -0.007 |
| 0.1 | 0.252 | 0.011 | -0.115 | -0.006 |
| 0.15 | 0.212 | 0.009 | -0.09 | -0.004 |
| 0.2 | 0.14 | 0.006 | -0.363^a^ | -0.017b |
| 0.25 | 0.217 | 0.009 | -0.275c | -0.012c |
| 0.3 | 0.181 | 0.007 | -0.227 | -0.01 |
| 0.35 | 0.063 | 0.003 | -0.284c | -0.012c |
| 0.4 | 0.01 | 0 | -0.36b | -0.015b |
| 0.45 | 0.015 | 0.001 | -0.328c | -0.014b |
| 0.5 | 0.106 | 0.004 | -0.413b | -0.017b |
| 0.55 | 0.019 | 0.001 | -0.396b | -0.016b |
| 0.6 | 0 | 0 | -0.542^a^ | -0.021^a^ |
| 0.65 | 0.095 | 0.003 | -0.628^a^ | -0.024^a^ |
| 0.7 | 0.077 | 0.003 | -0.883^a^ | -0.033^a^ |
| 0.75 | -0.012 | 0 | -0.829^a^ | -0.03^a^ |
| 0.8 | -0.037 | -0.001 | -0.968^a^ | -0.034^a^ |
| 0.85 | 0.067 | 0.002 | -1.356^a^ | -0.045^a^ |
| 0.9 | -0.015 | 0 | -1.46^a^ | -0.047^a^ |
| 0.95 | -0.067 | -0.002 | -2.383^a^ | -0.069^a^ |
| Income 75 | | | | |
| 0.05 | 0.789^a^ | 0.037^a^ | -0.099 | -0.005 |
| 0.1 | 0.327 | 0.015 | -0.226c | -0.011c |
| 0.15 | 0.256 | 0.011 | -0.286b | -0.014b |
| 0.2 | 0.141 | 0.006 | -0.576^a^ | -0.027^a^ |
| 0.25 | 0.194 | 0.008 | -0.607^a^ | -0.027^a^ |
| 0.3 | 0.166 | 0.007 | -0.606^a^ | -0.027^a^ |
| 0.35 | 0.013 | 0.001 | -0.635^a^ | -0.028^a^ |
| 0.4 | -0.094 | -0.004 | -0.709^a^ | -0.03^a^ |
| 0.45 | -0.14 | -0.005 | -0.719^a^ | -0.03^a^ |
| 0.5 | -0.119 | -0.005 | -0.84^a^ | -0.034^a^ |
| 0.55 | -0.193 | -0.007 | -0.937^a^ | -0.037^a^ |
| 0.6 | -0.265 | -0.01 | -1.002^a^ | -0.039^a^ |
| 0.65 | -0.173 | -0.006 | -1.112^a^ | -0.042^a^ |
| 0.7 | -0.189 | -0.007 | -1.399^a^ | -0.052^a^ |
| 0.75 | -0.314 | -0.011 | -1.46^a^ | -0.052^a^ |
| 0.8 | -0.346 | -0.012 | -1.454^a^ | -0.05^a^ |
| 0.85 | -0.378 | -0.012 | -1.789^a^ | -0.059^a^ |
| 0.9 | -0.534 | -0.017 | -1.81^a^ | -0.058^a^ |
| 0.95 | -0.862^c^ | -0.026 | -2.624^a^ | -0.076^a^ |
| Income 90 | | | | |
| 0.05 | 1.135^a^ | 0.054^a^ | -0.338c | -0.018c |
| 0.1 | 0.56^b^ | 0.025^b^ | -0.501^a^ | -0.025^a^ |
| 0.15 | 0.49^b^ | 0.021^b^ | -0.727^a^ | -0.035^a^ |
| 0.2 | 0.234 | 0.01 | -1.086^a^ | -0.051^a^ |
| 0.25 | 0.132 | 0.005 | -1.293^a^ | -0.059^a^ |
| 0.3 | 0.01 | 0 | -1.421^a^ | -0.063^a^ |
| 0.35 | -0.148 | -0.006 | -1.452^a^ | -0.063^a^ |
| 0.4 | -0.275 | -0.011 | -1.581^a^ | -0.067^a^ |
| 0.45 | -0.266 | -0.01 | -1.602^a^ | -0.066^a^ |
| 0.5 | -0.316 | -0.012^c^ | -1.76^a^ | -0.071^a^ |
| 0.55 | -0.37^c^ | -0.014^c^ | -1.695^a^ | -0.067^a^ |
| 0.6 | -0.422^b^ | -0.016^c^ | -1.787^a^ | -0.069^a^ |
| 0.65 | -0.296 | -0.011 | -1.957^a^ | -0.074^a^ |
| 0.7 | -0.431^c^ | -0.015^b^ | -2.3^a^ | -0.085^a^ |
| 0.75 | -0.519^b^ | -0.018^c^ | -2.443^a^ | -0.087^a^ |
| 0.8 | -0.641^b^ | -0.022^a^ | -2.341^a^ | -0.081^a^ |
| 0.85 | -0.788^b^ | -0.026^a^ | -2.828^a^ | -0.094^a^ |
| 0.9 | -0.958^b^ | -0.031^b^ | -2.693^a^ | -0.087^a^ |
| 0.95 | -1.508^a^ | -0.045^a^ | -3.877^a^ | -0.112^a^ |
| Primary | | | | |
| 0.05 | 0.161 | 0.008 | 0.035 | 0.002 |
| 0.1 | 0.17 | 0.008 | 0.129 | 0.006 |
| 0.15 | 0.126 | 0.005 | -0.021 | -0.001 |
| 0.2 | 0.149 | 0.006 | -0.036 | -0.002 |
| 0.25 | -0.084 | -0.003 | -0.107 | -0.005 |
| 0.3 | -0.066 | -0.003 | -0.216b | -0.01b |
| 0.35 | -0.111 | -0.004 | -0.309^a^ | -0.013b |
| 0.4 | -0.145 | -0.006 | -0.419^a^ | -0.018^a^ |
| 0.45 | 0.045 | 0.002 | -0.58^a^ | -0.024^a^ |
| 0.5 | 0.142 | 0.005 | -0.783^a^ | -0.032^a^ |
| 0.55 | 0.075 | 0.003 | -0.908^a^ | -0.036^a^ |
| 0.6 | 0.055 | 0.002 | -0.971^a^ | -0.038^a^ |
| 0.65 | 0 | 0 | -1.326^a^ | -0.05^a^ |
| 0.7 | -0.098 | -0.004 | -1.628^a^ | -0.06^a^ |
| 0.75 | 0.04 | 0.001 | -1.74^a^ | -0.062^a^ |
| 0.8 | -0.011 | 0 | -2.141^a^ | -0.074^a^ |
| 0.85 | -0.262 | -0.009 | -2.609^a^ | -0.086^a^ |
| 0.9 | -0.239 | -0.008 | -2.684^a^ | -0.087^a^ |
| 0.95 | -0.573 | -0.017 | -4.037^a^ | -0.117^a^ |
| Secondary | | | | |
| 0.05 | 0.222 | 0.01 | -0.069 | -0.004 |
| 0.1 | 0.202 | 0.009 | -0.167 | -0.008 |
| 0.15 | 0.117 | 0.005 | -0.525^a^ | -0.025^a^ |
| 0.2 | 0.075 | 0.003 | -0.713^a^ | -0.033^a^ |
| 0.25 | -0.135 | -0.006 | -0.951^a^ | -0.043^a^ |
| 0.3 | -0.245 | -0.01 | -1.176^a^ | -0.052^a^ |
| 0.35 | -0.331^b^ | -0.013^c^ | -1.369^a^ | -0.059^a^ |
| 0.4 | -0.454^a^ | -0.018^a^ | -1.602^a^ | -0.068^a^ |
| 0.45 | -0.281^c^ | -0.011 | -1.896^a^ | -0.078^a^ |
| 0.5 | -0.165 | -0.006 | -2.183^a^ | -0.088^a^ |
| 0.55 | -0.287 | -0.011^c^ | -2.273^a^ | -0.09^a^ |
| 0.6 | -0.348^c^ | -0.013^c^ | -2.325^a^ | -0.09^a^ |
| 0.65 | -0.408^b^ | -0.015^c^ | -2.73^a^ | -0.103^a^ |
| 0.7 | -0.534^b^ | -0.019^b^ | -3.027^a^ | -0.112^a^ |
| 0.75 | -0.462^b^ | -0.016^c^ | -3.297^a^ | -0.117^a^ |
| 0.8 | -0.479^c^ | -0.017^b^ | -3.544^a^ | -0.123^a^ |
| 0.85 | -0.871^a^ | -0.028^a^ | -4.179^a^ | -0.138^a^ |
| 0.9 | -0.853^b^ | -0.027^b^ | -3.98^a^ | -0.128^a^ |
| 0.95 | -1.384^a^ | -0.041^a^ | -5.551^a^ | -0.16^a^ |
| Social Class II | | | | |
| 0.05 | -0.145 | -0.007 | 0.233 | 0.012 |
| 0.1 | -0.132 | -0.006 | 0.383c | 0.019b |
| 0.15 | 0.005 | 0 | 0.53^a^ | 0.025^a^ |
| 0.2 | 0.148 | 0.006 | 0.558^a^ | 0.026^a^ |
| 0.25 | 0.01 | 0 | 0.496b | 0.022^a^ |
| 0.3 | -0.013 | -0.001 | 0.626^a^ | 0.028^a^ |
| 0.35 | 0.015 | 0.001 | 0.82^a^ | 0.036^a^ |
| 0.4 | 0.026 | 0.001 | 0.949^a^ | 0.04^a^ |
| 0.45 | 0.093 | 0.004 | 0.931^a^ | 0.039^a^ |
| 0.5 | 0.047 | 0.002 | 0.97^a^ | 0.039^a^ |
| 0.55 | 0.112 | 0.004 | 0.995^a^ | 0.039^a^ |
| 0.6 | -0.026 | -0.001 | 0.801^a^ | 0.031^a^ |
| 0.65 | 0.057 | 0.002 | 0.82^a^ | 0.031^a^ |
| 0.7 | 0.032 | 0.001 | 0.948^a^ | 0.035^a^ |
| 0.75 | -0.132 | -0.005 | 0.914^a^ | 0.033^a^ |
| 0.8 | -0.228 | -0.008 | 0.684^a^ | 0.024^a^ |
| 0.85 | 0.027 | 0.001 | 0.473c | 0.016b |
| 0.9 | -0.087 | -0.003 | 0.433 | 0.014 |
| 0.95 | 0.262 | 0.008 | 0.084 | 0.002 |
| Social Class III | | | | |
| 0.05 | -0.013 | -0.001 | 0.496^a^ | 0.026^a^ |
| 0.1 | 0.224 | 0.01 | 0.755^a^ | 0.037^a^ |
| 0.15 | 0.35^b^ | 0.015^b^ | 0.889^a^ | 0.042^a^ |
| 0.2 | 0.399^a^ | 0.017^a^ | 1.098^a^ | 0.051^a^ |
| 0.25 | 0.335^b^ | 0.014^b^ | 1.263^a^ | 0.057^a^ |
| 0.3 | 0.359^b^ | 0.015^b^ | 1.358^a^ | 0.06^a^ |
| 0.35 | 0.351^b^ | 0.014^b^ | 1.491^a^ | 0.065^a^ |
| 0.4 | 0.324^b^ | 0.013^b^ | 1.426^a^ | 0.06^a^ |
| 0.45 | 0.4^b^ | 0.016^a^ | 1.486^a^ | 0.061^a^ |
| 0.5 | 0.487^a^ | 0.019^a^ | 1.559^a^ | 0.063^a^ |
| 0.55 | 0.507^a^ | 0.019^a^ | 1.561^a^ | 0.062^a^ |
| 0.6 | 0.393^b^ | 0.014^b^ | 1.4^a^ | 0.054^a^ |
| 0.65 | 0.456^a^ | 0.017^a^ | 1.444^a^ | 0.055^a^ |
| 0.7 | 0.523^a^ | 0.019^a^ | 1.674^a^ | 0.062^a^ |
| 0.75 | 0.443^b^ | 0.015^a^ | 1.689^a^ | 0.06^a^ |
| 0.8 | 0.292 | 0.01 | 1.525^a^ | 0.053^a^ |
| 0.85 | 0.666^a^ | 0.022^b^ | 1.422^a^ | 0.047^a^ |
| 0.9 | 0.569^c^ | 0.018^b^ | 1.242^a^ | 0.04^a^ |
| 0.95 | 1.003^a^ | 0.03^b^ | 1.132^a^ | 0.033^a^ |
| Social Class IV | | | | |
| 0.05 | -0.421^b^ | -0.02^b^ | 0.756^a^ | 0.039^a^ |
| 0.1 | 0.056 | 0.003 | 0.979^a^ | 0.048^a^ |
| 0.15 | 0.194 | 0.008 | 1.234^a^ | 0.059^a^ |
| 0.2 | 0.321^b^ | 0.014^b^ | 1.601^a^ | 0.074^a^ |
| 0.25 | 0.274^c^ | 0.011 | 1.8^a^ | 0.081^a^ |
| 0.3 | 0.343^b^ | 0.014^b^ | 2.049^a^ | 0.091^a^ |
| 0.35 | 0.399^a^ | 0.016^b^ | 2.283^a^ | 0.099^a^ |
| 0.4 | 0.49^a^ | 0.019^a^ | 2.349^a^ | 0.099^a^ |
| 0.45 | 0.516^a^ | 0.02^a^ | 2.446^a^ | 0.101^a^ |
| 0.5 | 0.58^a^ | 0.022^a^ | 2.598^a^ | 0.105^a^ |
| 0.55 | 0.569^a^ | 0.021^a^ | 2.591^a^ | 0.103^a^ |
| 0.6 | 0.459^a^ | 0.017^a^ | 2.458^a^ | 0.095^a^ |
| 0.65 | 0.567^a^ | 0.021^a^ | 2.493^a^ | 0.094^a^ |
| 0.7 | 0.657^a^ | 0.023^a^ | 2.667^a^ | 0.098^a^ |
| 0.75 | 0.593^a^ | 0.021^a^ | 2.734^a^ | 0.097^a^ |
| 0.8 | 0.405^b^ | 0.014^b^ | 2.576^a^ | 0.089^a^ |
| 0.85 | 0.929^a^ | 0.03^a^ | 2.595^a^ | 0.086^a^ |
| 0.9 | 0.684^b^ | 0.022^b^ | 2.293^a^ | 0.074^a^ |
| 0.95 | 0.72^c^ | 0.022^c^ | 2.642^a^ | 0.076^a^ |
| Social Class V | | | | |
| 0.05 | -0.394^c^ | -0.019^c^ | 0.647^a^ | 0.034^a^ |
| 0.1 | -0.145 | -0.006 | 0.907^a^ | 0.044^a^ |
| 0.15 | 0.184 | 0.008 | 1.211^a^ | 0.058^a^ |
| 0.2 | 0.309^c^ | 0.013^c^ | 1.528^a^ | 0.071^a^ |
| 0.25 | 0.463^a^ | 0.019^a^ | 1.771^a^ | 0.08^a^ |
| 0.3 | 0.479^a^ | 0.019^a^ | 1.98^a^ | 0.088^a^ |
| 0.35 | 0.559^a^ | 0.022^a^ | 2.172^a^ | 0.094^a^ |
| 0.4 | 0.582^a^ | 0.023^a^ | 2.236^a^ | 0.095^a^ |
| 0.45 | 0.643^a^ | 0.025^a^ | 2.322^a^ | 0.096^a^ |
| 0.5 | 0.771^a^ | 0.029^a^ | 2.488^a^ | 0.1^a^ |
| 0.55 | 0.844^a^ | 0.032^a^ | 2.466^a^ | 0.098^a^ |
| 0.6 | 0.718^a^ | 0.027^a^ | 2.215^a^ | 0.086^a^ |
| 0.65 | 0.645^a^ | 0.023^a^ | 2.258^a^ | 0.086^a^ |
| 0.7 | 0.682^a^ | 0.024^a^ | 2.446^a^ | 0.09^a^ |
| 0.75 | 0.649^a^ | 0.023^a^ | 2.428^a^ | 0.087^a^ |
| 0.8 | 0.535^b^ | 0.018^b^ | 2.416^a^ | 0.084^a^ |
| 0.85 | 0.905^a^ | 0.03^a^ | 2.511^a^ | 0.083^a^ |
| 0.9 | 0.875^b^ | 0.028^a^ | 2.021^a^ | 0.065^a^ |
| 0.95 | 1.423^a^ | 0.042^a^ | 2.155^a^ | 0.062^a^ |
| Social Class VI | | | | |
| 0.05 | -0.704^a^ | -0.033^a^ | 0.695^a^ | 0.036^a^ |
| 0.1 | -0.277 | -0.012 | 1.102^a^ | 0.054^a^ |
| 0.15 | 0.052 | 0.002 | 1.444^a^ | 0.069^a^ |
| 0.2 | 0.148 | 0.006 | 1.727^a^ | 0.08^a^ |
| 0.25 | 0.238 | 0.01 | 1.922^a^ | 0.087^a^ |
| 0.3 | 0.313^c^ | 0.013^c^ | 2.192^a^ | 0.097^a^ |
| 0.35 | 0.418^b^ | 0.017^b^ | 2.518^a^ | 0.109^a^ |
| 0.4 | 0.493^a^ | 0.019^a^ | 2.456^a^ | 0.104^a^ |
| 0.45 | 0.466^a^ | 0.018^b^ | 2.59^a^ | 0.107^a^ |
| 0.5 | 0.438^b^ | 0.017^b^ | 2.797^a^ | 0.113^a^ |
| 0.55 | 0.573^a^ | 0.022^a^ | 2.662^a^ | 0.105^a^ |
| 0.6 | 0.493^b^ | 0.018^b^ | 2.66^a^ | 0.103^a^ |
| 0.65 | 0.515^b^ | 0.019^a^ | 2.828^a^ | 0.107^a^ |
| 0.7 | 0.624^a^ | 0.022^a^ | 3.14^a^ | 0.116^a^ |
| 0.75 | 0.503^b^ | 0.017^b^ | 3.216^a^ | 0.115^a^ |
| 0.8 | 0.395^c^ | 0.014^c^ | 3.29^a^ | 0.114^a^ |
| 0.85 | 0.924^a^ | 0.03^a^ | 3.352^a^ | 0.111^a^ |
| 0.9 | 1.092^a^ | 0.035^a^ | 3.286^a^ | 0.106^a^ |
| 0.95 | 1.548^a^ | 0.046^a^ | 3.585^a^ | 0.103^a^ |
| Universitaryersitary | | | | |
| 0.05 | 0.695^a^ | 0.033^a^ | -0.443^a^ | -0.023^a^ |
| 0.1 | 0.183 | 0.008 | -0.777^a^ | -0.038^a^ |
| 0.15 | -0.093 | -0.004 | -1.351^a^ | -0.065^a^ |
| 0.2 | -0.173 | -0.007 | -1.76^a^ | -0.082^a^ |
| 0.25 | -0.497^a^ | -0.021^b^ | -2.175^a^ | -0.098^a^ |
| 0.3 | -0.568^a^ | -0.023^a^ | -2.473^a^ | -0.11^a^ |
| 0.35 | -0.844^a^ | -0.034^a^ | -2.747^a^ | -0.119^a^ |
| 0.4 | -0.977^a^ | -0.039^a^ | -3.01^a^ | -0.127^a^ |
| 0.45 | -0.873^a^ | -0.034^a^ | -3.327^a^ | -0.138^a^ |
| 0.5 | -0.742^a^ | -0.028^a^ | -3.595^a^ | -0.145^a^ |
| 0.55 | -0.86^a^ | -0.032^a^ | -3.679^a^ | -0.146^a^ |
| 0.6 | -0.95^a^ | -0.035^a^ | -3.72^a^ | -0.145^a^ |
| 0.65 | -1.027^a^ | -0.037^a^ | -4.08^a^ | -0.155^a^ |
| 0.7 | -1.201^a^ | -0.043^a^ | -4.454^a^ | -0.164^a^ |
| 0.75 | -1.161^a^ | -0.04^a^ | -4.678^a^ | -0.167^a^ |
| 0.8 | -1.181^a^ | -0.041^a^ | -4.856^a^ | -0.168^a^ |
| 0.85 | -1.774^a^ | -0.058^a^ | -5.43^a^ | -0.18^a^ |
| 0.9 | -1.683^a^ | -0.054^a^ | -5.185^a^ | -0.167^a^ |
| 0.95 | -2.386^a^ | -0.071^a^ | -6.707^a^ | -0.194^a^ |
| 2014 | | | | |
| Income 100 | | | | |
| 0.05 | 0.789^b^ | 0.037^b^ | -0.425 | -0.022 |
| 0.1 | 0.678^b^ | 0.031^b^ | -0.9^a^ | -0.044^a^ |
| 0.15 | 0.149 | 0.007 | -1.333^a^ | -0.064^a^ |
| 0.2 | 0.011 | 0 | -1.37^a^ | -0.064^a^ |
| 0.25 | -0.245 | -0.01 | -1.631^a^ | -0.074^a^ |
| 0.3 | -0.278 | -0.011 | -1.924^a^ | -0.085^a^ |
| 0.35 | -0.527^b^ | -0.021^c^ | -1.832^a^ | -0.079^a^ |
| 0.4 | -0.837^a^ | -0.033^a^ | -2.178^a^ | -0.092^a^ |
| 0.45 | -0.795^a^ | -0.031^a^ | -2.492^a^ | -0.103^a^ |
| 0.5 | -0.968^a^ | -0.037^a^ | -2.398^a^ | -0.097^a^ |
| 0.55 | -1.041^a^ | -0.039^a^ | -2.564^a^ | -0.101^a^ |
| 0.6 | -1.541^a^ | -0.057^a^ | -2.887^a^ | -0.112^a^ |
| 0.65 | -1.38^a^ | -0.05^a^ | -3.055^a^ | -0.115^a^ |
| 0.7 | -1.325^a^ | -0.047^a^ | -3.345^a^ | -0.123^a^ |
| 0.75 | -1.14^a^ | -0.04^a^ | -3.372^a^ | -0.12^a^ |
| 0.8 | -1.269^a^ | -0.043^a^ | -3.959^a^ | -0.136^a^ |
| 0.85 | -1.518^a^ | -0.05^a^ | -4.307^a^ | -0.141^a^ |
| 0.9 | -1.417^a^ | -0.045^b^ | -3.505^a^ | -0.109^a^ |
| 0.95 | -2.486^a^ | -0.072^a^ | -3.792^a^ | -0.107^a^ |
| Income 25 | | | | |
| 0.05 | 0.002 | 0 | 0.1 | 0.005 |
| 0.1 | -0.343 | -0.015 | 0.169 | 0.008 |
| 0.15 | -0.308 | -0.013 | 0.219 | 0.011 |
| 0.2 | -0.463^b^ | -0.02^b^ | 0.161 | 0.007 |
| 0.25 | -0.4^c^ | -0.017^b^ | 0.119 | 0.005 |
| 0.3 | -0.387^c^ | -0.016^c^ | 0.206 | 0.009 |
| 0.35 | -0.417^c^ | -0.017^c^ | 0.197 | 0.009 |
| 0.4 | -0.449^b^ | -0.018^c^ | 0.07 | 0.003 |
| 0.45 | -0.291 | -0.011 | 0.023 | 0.001 |
| 0.5 | -0.411^c^ | -0.016 | -0.076 | -0.003 |
| 0.55 | -0.443^c^ | -0.017^c^ | -0.15 | -0.006 |
| 0.6 | -0.626^b^ | -0.023^b^ | -0.324 | -0.013 |
| 0.65 | -0.505^c^ | -0.018^c^ | -0.367 | -0.014 |
| 0.7 | -0.556^c^ | -0.02^c^ | -0.759b | -0.028b |
| 0.75 | -0.62^c^ | -0.022^b^ | -0.967b | -0.034b |
| 0.8 | -0.71^c^ | -0.024^b^ | -1.675^a^ | -0.057^a^ |
| 0.85 | -0.887^b^ | -0.029^b^ | -1.418^a^ | -0.047b |
| 0.9 | -0.882^c^ | -0.028^c^ | -1.185b | -0.037b |
| 0.95 | -1.754^b^ | -0.051^b^ | -1.645c | -0.046c |
| Income 50 | | | | |
| 0.05 | 0.288 | 0.014 | -0.096 | -0.005 |
| 0.1 | 0.29 | 0.013 | 0.138 | 0.007 |
| 0.15 | 0.224 | 0.01 | 0.195 | 0.009 |
| 0.2 | 0.143 | 0.006 | 0.123 | 0.006 |
| 0.25 | 0.102 | 0.004 | 0.111 | 0.005 |
| 0.3 | 0.12 | 0.005 | 0.167 | 0.007 |
| 0.35 | 0.072 | 0.003 | 0.033 | 0.001 |
| 0.4 | -0.08 | -0.003 | -0.057 | -0.002 |
| 0.45 | -0.149 | -0.006 | 0.024 | 0.001 |
| 0.5 | -0.113 | -0.004 | 0.127 | 0.005 |
| 0.55 | -0.019 | -0.001 | 0.119 | 0.005 |
| 0.6 | -0.23 | -0.009 | -0.131 | -0.005 |
| 0.65 | -0.137 | -0.005 | -0.124 | -0.005 |
| 0.7 | -0.282 | -0.01 | -0.359 | -0.013 |
| 0.75 | -0.513 | -0.018^c^ | -0.096 | -0.003 |
| 0.8 | -0.798^b^ | -0.027^b^ | -0.475 | -0.016 |
| 0.85 | -0.797^c^ | -0.026^b^ | -0.662 | -0.022 |
| 0.9 | -0.835^c^ | -0.026 | -0.017 | -0.001 |
| 0.95 | -1.689^b^ | -0.049^b^ | -1.174 | -0.033 |
| Income 75 | | | | |
| 0.05 | 0.498 | 0.023 | 0.051 | 0.003 |
| 0.1 | 0.423^c^ | 0.019^c^ | 0.147 | 0.007 |
| 0.15 | 0.148 | 0.006 | 0.145 | 0.007 |
| 0.2 | 0.033 | 0.001 | -0.034 | -0.002 |
| 0.25 | -0.114 | -0.005 | -0.093 | -0.004 |
| 0.3 | 0.006 | 0 | -0.156 | -0.007 |
| 0.35 | -0.003 | 0 | -0.292 | -0.013 |
| 0.4 | -0.1 | -0.004 | -0.584b | -0.025b |
| 0.45 | -0.046 | -0.002 | -0.81^a^ | -0.034^a^ |
| 0.5 | -0.204 | -0.008 | -1.022^a^ | -0.041^a^ |
| 0.55 | -0.301 | -0.011 | -1.134^a^ | -0.045^a^ |
| 0.6 | -0.448^c^ | -0.017^b^ | -1.479^a^ | -0.057^a^ |
| 0.65 | -0.338 | -0.012 | -1.359^a^ | -0.051^a^ |
| 0.7 | -0.372 | -0.013 | -1.541^a^ | -0.057^a^ |
| 0.75 | -0.356 | -0.012 | -1.581^a^ | -0.056^a^ |
| 0.8 | -0.673^b^ | -0.023^b^ | -2.158^a^ | -0.074^a^ |
| 0.85 | -0.744^b^ | -0.024^b^ | -2.331^a^ | -0.076^a^ |
| 0.9 | -0.731^c^ | -0.023 | -2.077^a^ | -0.064^a^ |
| 0.95 | -1.769^b^ | -0.051^a^ | -2.52^a^ | -0.071^a^ |
| Income 90 | | | | |
| 0.05 | 0.285 | 0.013 | -0.328 | -0.017 |
| 0.1 | 0.196 | 0.009 | -0.318 | -0.016 |
| 0.15 | -0.072 | -0.003 | -0.557b | -0.027b |
| 0.2 | -0.246 | -0.01 | -0.737^a^ | -0.034^a^ |
| 0.25 | -0.314 | -0.013 | -0.757^a^ | -0.034^a^ |
| 0.3 | -0.326 | -0.013^c^ | -0.873^a^ | -0.039^a^ |
| 0.35 | -0.402^c^ | -0.016^c^ | -0.863^a^ | -0.037^a^ |
| 0.4 | -0.471^b^ | -0.019^b^ | -1.252^a^ | -0.053^a^ |
| 0.45 | -0.496^b^ | -0.019^b^ | -1.408^a^ | -0.058^a^ |
| 0.5 | -0.671^a^ | -0.026^a^ | -1.496^a^ | -0.061^a^ |
| 0.55 | -0.761^a^ | -0.029^a^ | -1.73^a^ | -0.068^a^ |
| 0.6 | -1.081^a^ | -0.04^a^ | -2.005^a^ | -0.078^a^ |
| 0.65 | -1.1^a^ | -0.04^a^ | -2.197^a^ | -0.083^a^ |
| 0.7 | -1.128^a^ | -0.04^a^ | -2.467^a^ | -0.091^a^ |
| 0.75 | -1.179^a^ | -0.041^a^ | -2.471^a^ | -0.088^a^ |
| 0.8 | -1.359^a^ | -0.046^a^ | -3.174^a^ | -0.109^a^ |
| 0.85 | -1.494^a^ | -0.049^a^ | -3.138^a^ | -0.103^a^ |
| 0.9 | -1.517^a^ | -0.048^a^ | -2.776^a^ | -0.086^a^ |
| 0.95 | -2.384^a^ | -0.069^a^ | -3.174^a^ | -0.089^a^ |
| Primary | | | | |
| 0.05 | -0.207 | -0.01 | 0.041 | 0.002 |
| 0.1 | -0.101 | -0.005 | -0.002 | 0 |
| 0.15 | -0.041 | -0.002 | -0.02 | -0.001 |
| 0.2 | 0.013 | 0.001 | -0.115 | -0.005 |
| 0.25 | -0.001 | 0 | -0.291b | -0.013b |
| 0.3 | 0.041 | 0.002 | -0.358^a^ | -0.016^a^ |
| 0.35 | 0.108 | 0.004 | -0.364^a^ | -0.016^a^ |
| 0.4 | -0.003 | 0 | -0.425^a^ | -0.018^a^ |
| 0.45 | -0.008 | 0 | -0.523^a^ | -0.022^a^ |
| 0.5 | -0.1 | -0.004 | -0.567^a^ | -0.023^a^ |
| 0.55 | -0.139 | -0.005 | -0.741^a^ | -0.029^a^ |
| 0.6 | -0.191 | -0.007 | -0.802^a^ | -0.031^a^ |
| 0.65 | -0.023 | -0.001 | -0.923^a^ | -0.035^a^ |
| 0.7 | -0.128 | -0.005 | -1.124^a^ | -0.041^a^ |
| 0.75 | -0.132 | -0.005 | -1.467^a^ | -0.052^a^ |
| 0.8 | -0.285 | -0.01 | -1.926^a^ | -0.066^a^ |
| 0.85 | -0.43 | -0.014 | -2.208^a^ | -0.072^a^ |
| 0.9 | -0.789^c^ | -0.025^c^ | -2.293^a^ | -0.071^a^ |
| 0.95 | -1.386^b^ | -0.04^b^ | -2.56^a^ | -0.072^a^ |
| Secondary | | | | |
| 0.05 | 0.064 | 0.003 | -0.269b | -0.014b |
| 0.1 | 0.144 | 0.007 | -0.449^a^ | -0.022^a^ |
| 0.15 | 0.026 | 0.001 | -0.614^a^ | -0.029^a^ |
| 0.2 | 0.029 | 0.001 | -0.87^a^ | -0.04^a^ |
| 0.25 | -0.077 | -0.003 | -1.043^a^ | -0.047^a^ |
| 0.3 | -0.09 | -0.004 | -1.295^a^ | -0.057^a^ |
| 0.35 | -0.058 | -0.002 | -1.366^a^ | -0.059^a^ |
| 0.4 | -0.227 | -0.009 | -1.621^a^ | -0.069^a^ |
| 0.45 | -0.238 | -0.009 | -1.871^a^ | -0.078^a^ |
| 0.5 | -0.277 | -0.011 | -1.97^a^ | -0.08^a^ |
| 0.55 | -0.436^b^ | -0.016^b^ | -2.337^a^ | -0.092^a^ |
| 0.6 | -0.632^a^ | -0.023^a^ | -2.562^a^ | -0.099^a^ |
| 0.65 | -0.563^b^ | -0.02^a^ | -2.697^a^ | -0.101^a^ |
| 0.7 | -0.751^a^ | -0.027^a^ | -2.94^a^ | -0.108^a^ |
| 0.75 | -0.824^a^ | -0.029^a^ | -3.37^a^ | -0.12^a^ |
| 0.8 | -1.097^a^ | -0.037^a^ | -3.99^a^ | -0.137^a^ |
| 0.85 | -1.13^a^ | -0.037^a^ | -4.534^a^ | -0.149^a^ |
| 0.9 | -1.603^a^ | -0.05^a^ | -4.251^a^ | -0.132^a^ |
| 0.95 | -2.125^a^ | -0.061^a^ | -4.625^a^ | -0.13^a^ |
| Social Class II | | | | |
| 0.05 | 0.066 | 0.003 | 0.335 | 0.017 |
| 0.1 | -0.147 | -0.007 | 0.373 | 0.018 |
| 0.15 | -0.112 | -0.005 | 0.709^a^ | 0.034^a^ |
| 0.2 | -0.112 | -0.005 | 0.725^a^ | 0.034^a^ |
| 0.25 | -0.064 | -0.003 | 0.679^a^ | 0.031^a^ |
| 0.3 | -0.037 | -0.002 | 0.753^a^ | 0.033^a^ |
| 0.35 | 0.062 | 0.003 | 0.849^a^ | 0.037^a^ |
| 0.4 | 0.155 | 0.006 | 0.71^a^ | 0.03^a^ |
| 0.45 | 0.126 | 0.005 | 0.638^a^ | 0.026b |
| 0.5 | 0.242 | 0.009 | 0.83^a^ | 0.034^a^ |
| 0.55 | 0.376^c^ | 0.014^c^ | 0.564b | 0.022b |
| 0.6 | 0.292 | 0.011 | 0.442c | 0.017c |
| 0.65 | 0.322 | 0.012 | 0.523b | 0.02b |
| 0.7 | 0.4 | 0.014^c^ | 0.58b | 0.021b |
| 0.75 | 0.345 | 0.012 | 0.702^a^ | 0.025b |
| 0.8 | 0.407 | 0.014 | 0.718^a^ | 0.025b |
| 0.85 | 0.167 | 0.005 | 0.457 | 0.015 |
| 0.9 | 0.026 | 0.001 | -0.018 | -0.001 |
| 0.95 | 0.843^c^ | 0.024^c^ | -0.179 | -0.005 |
| Social Class III | | | | |
| 0.05 | 0.082 | 0.004 | 0.446b | 0.023c |
| 0.1 | 0.064 | 0.003 | 0.643^a^ | 0.032^a^ |
| 0.15 | 0.2 | 0.009 | 0.885^a^ | 0.042^a^ |
| 0.2 | 0.216 | 0.009 | 1.113^a^ | 0.052^a^ |
| 0.25 | 0.19 | 0.008 | 1.117^a^ | 0.051^a^ |
| 0.3 | 0.23 | 0.009^c^ | 1.16^a^ | 0.051^a^ |
| 0.35 | 0.211 | 0.008 | 1.172^a^ | 0.051^a^ |
| 0.4 | 0.291^c^ | 0.012^c^ | 1.027^a^ | 0.044^a^ |
| 0.45 | 0.321^b^ | 0.012^c^ | 1.164^a^ | 0.048^a^ |
| 0.5 | 0.391^b^ | 0.015^b^ | 1.254^a^ | 0.051^a^ |
| 0.55 | 0.408^b^ | 0.015^b^ | 1.35^a^ | 0.053^a^ |
| 0.6 | 0.333^c^ | 0.012^c^ | 1.233^a^ | 0.048^a^ |
| 0.65 | 0.339^c^ | 0.012^c^ | 1.391^a^ | 0.052^a^ |
| 0.7 | 0.466^b^ | 0.017^b^ | 1.634^a^ | 0.06^a^ |
| 0.75 | 0.543^a^ | 0.019^a^ | 1.764^a^ | 0.063^a^ |
| 0.8 | 0.602^a^ | 0.02^a^ | 1.825^a^ | 0.063^a^ |
| 0.85 | 0.586^b^ | 0.019^b^ | 1.589^a^ | 0.052^a^ |
| 0.9 | 0.609^b^ | 0.019^b^ | 1.285^a^ | 0.04^a^ |
| 0.95 | 1.906^a^ | 0.055^a^ | 1.127^a^ | 0.032^a^ |
| Social Class IV | | | | |
| 0.05 | 0.134 | 0.006 | 0.866^a^ | 0.044^a^ |
| 0.1 | 0.203 | 0.009 | 1.128^a^ | 0.056^a^ |
| 0.15 | 0.422^b^ | 0.018^a^ | 1.502^a^ | 0.072^a^ |
| 0.2 | 0.468^a^ | 0.02^a^ | 1.737^a^ | 0.081^a^ |
| 0.25 | 0.489^a^ | 0.02^a^ | 1.732^a^ | 0.078^a^ |
| 0.3 | 0.577^a^ | 0.023^a^ | 1.822^a^ | 0.081^a^ |
| 0.35 | 0.686^a^ | 0.028^a^ | 2.032^a^ | 0.088^a^ |
| 0.4 | 0.809^a^ | 0.032^a^ | 2.162^a^ | 0.092^a^ |
| 0.45 | 0.84^a^ | 0.033^a^ | 2.286^a^ | 0.095^a^ |
| 0.5 | 0.822^a^ | 0.031^a^ | 2.458^a^ | 0.1^a^ |
| 0.55 | 0.953^a^ | 0.036^a^ | 2.519^a^ | 0.1^a^ |
| 0.6 | 0.881^a^ | 0.033^a^ | 2.472^a^ | 0.096^a^ |
| 0.65 | 0.962^a^ | 0.035^a^ | 2.716^a^ | 0.102^a^ |
| 0.7 | 1.111^a^ | 0.039^a^ | 2.734^a^ | 0.101^a^ |
| 0.75 | 1.094^a^ | 0.038^a^ | 2.736^a^ | 0.097^a^ |
| 0.8 | 0.979^a^ | 0.033^a^ | 2.936^a^ | 0.101^a^ |
| 0.85 | 0.842^a^ | 0.028^a^ | 2.466^a^ | 0.081^a^ |
| 0.9 | 0.822^a^ | 0.026^a^ | 2.309^a^ | 0.072^a^ |
| 0.95 | 1.319^a^ | 0.038^a^ | 2.615^a^ | 0.074^a^ |
| Social Class V | | | | |
| 0.05 | 0.069 | 0.003 | 0.727^a^ | 0.037^a^ |
| 0.1 | 0.129 | 0.006 | 1.075^a^ | 0.053^a^ |
| 0.15 | 0.357^b^ | 0.016^a^ | 1.467^a^ | 0.07^a^ |
| 0.2 | 0.457^a^ | 0.019^a^ | 1.865^a^ | 0.087^a^ |
| 0.25 | 0.443^a^ | 0.018^a^ | 1.879^a^ | 0.085^a^ |
| 0.3 | 0.509^a^ | 0.021^a^ | 2.01^a^ | 0.089^a^ |
| 0.35 | 0.632^a^ | 0.025^a^ | 2.144^a^ | 0.093^a^ |
| 0.4 | 0.803^a^ | 0.032^a^ | 2.29^a^ | 0.097^a^ |
| 0.45 | 0.842^a^ | 0.033^a^ | 2.462^a^ | 0.102^a^ |
| 0.5 | 0.946^a^ | 0.036^a^ | 2.553^a^ | 0.104^a^ |
| 0.55 | 1.002^a^ | 0.038^a^ | 2.637^a^ | 0.104^a^ |
| 0.6 | 1.143^a^ | 0.042^a^ | 2.665^a^ | 0.103^a^ |
| 0.65 | 1.114^a^ | 0.04^a^ | 2.839^a^ | 0.107^a^ |
| 0.7 | 1.195^a^ | 0.042^a^ | 2.942^a^ | 0.108^a^ |
| 0.75 | 1.297^a^ | 0.045^a^ | 2.908^a^ | 0.103^a^ |
| 0.8 | 1.409^a^ | 0.048^a^ | 3.096^a^ | 0.106^a^ |
| 0.85 | 1.471^a^ | 0.048^a^ | 2.982^a^ | 0.098^a^ |
| 0.9 | 1.557^a^ | 0.049^a^ | 2.852^a^ | 0.088^a^ |
| 0.95 | 2.481^a^ | 0.072^a^ | 2.885^a^ | 0.081^a^ |
| Social Class VI | | | | |
| 0.05 | -0.155 | -0.007 | 0.931^a^ | 0.048^a^ |
| 0.1 | -0.372 | -0.017 | 1.383^a^ | 0.068^a^ |
| 0.15 | -0.023 | -0.001 | 1.894^a^ | 0.091^a^ |
| 0.2 | 0.065 | 0.003 | 2.216^a^ | 0.103^a^ |
| 0.25 | 0.238 | 0.01 | 2.267^a^ | 0.103^a^ |
| 0.3 | 0.354^b^ | 0.014^c^ | 2.316^a^ | 0.103^a^ |
| 0.35 | 0.475^a^ | 0.019^a^ | 2.406^a^ | 0.104^a^ |
| 0.4 | 0.679^a^ | 0.027^a^ | 2.495^a^ | 0.106^a^ |
| 0.45 | 0.779^a^ | 0.03^a^ | 2.745^a^ | 0.114^a^ |
| 0.5 | 0.841^a^ | 0.032^a^ | 3.078^a^ | 0.125^a^ |
| 0.55 | 0.957^a^ | 0.036^a^ | 3.141^a^ | 0.124^a^ |
| 0.6 | 1.143^a^ | 0.042^a^ | 3.12^a^ | 0.121^a^ |
| 0.65 | 1.128^a^ | 0.041^a^ | 3.368^a^ | 0.127^a^ |
| 0.7 | 1.11^a^ | 0.039^a^ | 3.543^a^ | 0.13^a^ |
| 0.75 | 1.047^a^ | 0.036^a^ | 3.574^a^ | 0.127^a^ |
| 0.8 | 1.267^a^ | 0.043^a^ | 3.894^a^ | 0.134^a^ |
| 0.85 | 1.276^a^ | 0.042^a^ | 4.007^a^ | 0.131^a^ |
| 0.9 | 1.543^a^ | 0.049^a^ | 3.21^a^ | 0.1^a^ |
| 0.95 | 2.292^a^ | 0.066^a^ | 3.577^a^ | 0.101^a^ |
| Universitaryersitary | | | | |
| 0.05 | 0.189 | 0.009 | -0.691^a^ | -0.035^a^ |
| 0.1 | -0.032 | -0.001 | -1.217^a^ | -0.06^a^ |
| 0.15 | -0.423^b^ | -0.018^b^ | -1.667^a^ | -0.08^a^ |
| 0.2 | -0.494^a^ | -0.021^a^ | -2.181^a^ | -0.101^a^ |
| 0.25 | -0.71^a^ | -0.029^a^ | -2.39^a^ | -0.108^a^ |
| 0.3 | -0.785^a^ | -0.032^a^ | -2.616^a^ | -0.116^a^ |
| 0.35 | -0.828^a^ | -0.033^a^ | -2.822^a^ | -0.122^a^ |
| 0.4 | -1.069^a^ | -0.042^a^ | -3.154^a^ | -0.134^a^ |
| 0.45 | -1.095^a^ | -0.043^a^ | -3.564^a^ | -0.148^a^ |
| 0.5 | -1.123^a^ | -0.043^a^ | -3.737^a^ | -0.152^a^ |
| 0.55 | -1.348^a^ | -0.051^a^ | -4.154^a^ | -0.164^a^ |
| 0.6 | -1.643^a^ | -0.061^a^ | -4.397^a^ | -0.17^a^ |
| 0.65 | -1.585^a^ | -0.057^a^ | -4.586^a^ | -0.172^a^ |
| 0.7 | -1.839^a^ | -0.065^a^ | -4.755^a^ | -0.175^a^ |
| 0.75 | -1.941^a^ | -0.067^a^ | -5.038^a^ | -0.179^a^ |
| 0.8 | -2.185^a^ | -0.074^a^ | -5.636^a^ | -0.193^a^ |
| 0.85 | -2.361^a^ | -0.077^a^ | -6.11^a^ | -0.201^a^ |
| 0.9 | -2.721^a^ | -0.086^a^ | -5.939^a^ | -0.184^a^ |
| 0.95 | -3.976^a^ | -0.115^a^ | -6.152^a^ | -0.173^a^ |

**Sensibility analysis by kernel and bandwidth**

|  | Epanechnikov | | | Gaussian | | | Rectangular | | |
| --- | --- | --- | --- | --- | --- | --- | --- | --- | --- |
|  | Hardle | Scott | Silver | Hardle | Scott | Silver | Hardle | Scott | Silver |
| 1993 |  |  |  |  |  |  |  |  |  |
| Primary |  |  |  |  |  |  |  |  |  |
| men |  |  |  |  |  |  |  |  |  |
| 0.05 | 0.0068 | 0.0068 | 0.0067 | 0.0066 | 0.0066 | 0.0066 | 0.0063 | 0.0063 | 0.0066 |
| 0.1 | 0.0088 | 0.0089 | 0.0087 | 0.0088 | 0.0088 | 0.0087 | 0.0090 | 0.0091 | 0.0089 |
| 0.15 | 0.0167 | 0.0168 | 0.0167 | 0.0167 | 0.0167 | 0.0166 | 0.0168 | 0.0168 | 0.0172 |
| 0.2 | 0.0148 | 0.0148 | 0.0149 | 0.0149 | 0.0150 | 0.0149 | 0.0152 | 0.0143 | 0.0156 |
| 0.25 | 0.0157 | 0.0156 | 0.0158 | 0.0156 | 0.0155 | 0.0157 | 0.0166 | 0.0152 | 0.0148 |
| 0.3 | 0.0150 | 0.0150 | 0.0150 | 0.0150 | 0.0150 | 0.0151 | 0.0140 | 0.0150 | 0.0150 |
| 0.35 | 0.0100 | 0.0099 | 0.0101 | 0.0100 | 0.0100 | 0.0101 | 0.0100 | 0.0102 | 0.0103 |
| 0.4 | 0.0107 | 0.0105 | 0.0107 | 0.0105 | 0.0104 | 0.0106 | 0.0100 | 0.0099 | 0.0103 |
| 0.45 | 0.0100 | 0.0100 | 0.0100 | 0.0100 | 0.0100 | 0.0100 | 0.0100 | 0.0095 | 0.0101 |
| 0.5 | 0.0077 | 0.0077 | 0.0077 | 0.0078 | 0.0078 | 0.0078 | 0.0075 | 0.0079 | 0.0078 |
| 0.55 | 0.0041 | 0.0040 | 0.0041 | 0.0040 | 0.0040 | 0.0041 | 0.0039 | 0.0040 | 0.0040 |
| 0.6 | 0.0008 | 0.0008 | 0.0008 | 0.0008 | 0.0008 | 0.0008 | 0.0008 | 0.0008 | 0.0008 |
| 0.65 | 0.0025 | 0.0025 | 0.0025 | 0.0025 | 0.0025 | 0.0025 | 0.0025 | 0.0027 | 0.0025 |
| 0.7 | 0.0040 | 0.0040 | 0.0040 | 0.0040 | 0.0040 | 0.0040 | 0.0041 | 0.0041 | 0.0043 |
| 0.75 | 0.0089 | 0.0088 | 0.0089 | 0.0088 | 0.0087 | 0.0088 | 0.0080 | 0.0084 | 0.0085 |
| 0.8 | 0.0143 | 0.0142 | 0.0142 | 0.0142 | 0.0142 | 0.0142 | 0.0147 | 0.0139 | 0.0138 |
| 0.85 | 0.0058 | 0.0059 | 0.0058 | 0.0059 | 0.0059 | 0.0058 | 0.0065 | 0.0063 | 0.0054 |
| 0.9 | -0.0006 | -0.0006 | -0.0006 | -0.0006 | -0.0006 | -0.0006 | -0.0006 | -0.0006 | -0.0006 |
| 0.95 | -0.0063 | -0.0062 | -0.0063 | -0.0061 | -0.0060 | -0.0062 | -0.0060 | -0.0057 | -0.0067 |
| women |  |  |  |  |  |  |  |  |  |
| 0.05 | 0.0003 | 0.0003 | 0.0003 | 0.0003 | 0.0003 | 0.0003 | 0.0003 | 0.0003 | 0.0003 |
| 0.1 | 0.0008 | 0.0008 | 0.0008 | 0.0008 | 0.0008 | 0.0008 | 0.0008 | 0.0008 | 0.0008 |
| 0.15 | 0.0019 | 0.0019 | 0.0020 | 0.0019 | 0.0019 | 0.0020 | 0.0019 | 0.0019 | 0.0019 |
| 0.2 | -0.0004 | -0.0004 | -0.0004 | -0.0004 | -0.0004 | -0.0004 | -0.0004 | -0.0004 | -0.0004 |
| 0.25 | -0.0015 | -0.0015 | -0.0015 | -0.0015 | -0.0015 | -0.0015 | -0.0015 | -0.0015 | -0.0015 |
| 0.3 | -0.0040 | -0.0040 | -0.0040 | -0.0040 | -0.0040 | -0.0041 | -0.0040 | -0.0039 | -0.0042 |
| 0.35 | -0.0088 | -0.0088 | -0.0088 | -0.0088 | -0.0088 | -0.0088 | -0.0086 | -0.0085 | -0.0086 |
| 0.4 | -0.0121 | -0.0119 | -0.0121 | -0.0120 | -0.0119 | -0.0121 | -0.0123 | -0.0121 | -0.0120 |
| 0.45 | -0.0207 | -0.0206 | -0.0207 | -0.0206 | -0.0205 | -0.0206 | -0.0206 | -0.0202 | -0.0203 |
| 0.5 | -0.0302 | -0.0304 | -0.0302 | -0.0304 | -0.0306 | -0.0308 | -0.0300 | -0.0295 | -0.0311 |
| 0.55 | -0.0339 | -0.0340 | -0.0338 | -0.0340 | -0.0342 | -0.0340 | -0.0361 | -0.0359 | -0.0342 |
| 0.6 | -0.0419 | -0.0417 | -0.0420 | -0.0416 | -0.0414 | -0.0417 | -0.0397 | -0.0421 | -0.0409 |
| 0.65 | -0.0451 | -0.0448 | -0.0453 | -0.0450 | -0.0447 | -0.0451 | -0.0452 | -0.0442 | -0.0448 |
| 0.7 | -0.0547 | -0.0551 | -0.0548 | -0.0553 | -0.0554 | -0.0553 | -0.0524 | -0.0597 | -0.0545 |
| 0.75 | -0.0637 | -0.0631 | -0.0638 | -0.0634 | -0.0631 | -0.0634 | -0.0618 | -0.0653 | -0.0626 |
| 0.8 | -0.0763 | -0.0756 | -0.0765 | -0.0757 | -0.0751 | -0.0759 | -0.0754 | -0.0775 | -0.0763 |
| 0.85 | -0.0845 | -0.0843 | -0.0845 | -0.0849 | -0.0854 | -0.0847 | -0.0951 | -0.0886 | -0.0908 |
| 0.9 | -0.1008 | -0.1027 | -0.1005 | -0.1023 | -0.1032 | -0.1019 | -0.1067 | -0.1019 | -0.1024 |
| 0.95 | -0.1241 | -0.1248 | -0.1240 | -0.1264 | -0.1282 | -0.1278 | -0.1090 | -0.1332 | -0.1172 |
| Secondary |  |  |  |  |  |  |  |  |  |
| men |  |  |  |  |  |  |  |  |  |
| 0.05 | -0.0006 | -0.0006 | -0.0006 | -0.0006 | -0.0006 | -0.0006 | -0.0006 | -0.0006 | -0.0006 |
| 0.1 | 0.0065 | 0.0065 | 0.0064 | 0.0065 | 0.0065 | 0.0064 | 0.0066 | 0.0067 | 0.0066 |
| 0.15 | 0.0090 | 0.0091 | 0.0090 | 0.0090 | 0.0091 | 0.0090 | 0.0091 | 0.0091 | 0.0093 |
| 0.2 | 0.0038 | 0.0038 | 0.0039 | 0.0039 | 0.0039 | 0.0039 | 0.0039 | 0.0037 | 0.0040 |
| 0.25 | 0.0012 | 0.0012 | 0.0012 | 0.0012 | 0.0012 | 0.0012 | 0.0013 | 0.0012 | 0.0012 |
| 0.3 | 0.0005 | 0.0005 | 0.0005 | 0.0005 | 0.0005 | 0.0005 | 0.0005 | 0.0005 | 0.0005 |
| 0.35 | -0.0092 | -0.0091 | -0.0092 | -0.0092 | -0.0091 | -0.0092 | -0.0092 | -0.0093 | -0.0094 |
| 0.4 | -0.0090 | -0.0089 | -0.0090 | -0.0089 | -0.0088 | -0.0089 | -0.0084 | -0.0083 | -0.0087 |
| 0.45 | -0.0111 | -0.0111 | -0.0111 | -0.0111 | -0.0111 | -0.0111 | -0.0111 | -0.0106 | -0.0111 |
| 0.5 | -0.0134 | -0.0134 | -0.0135 | -0.0136 | -0.0136 | -0.0136 | -0.0132 | -0.0139 | -0.0137 |
| 0.55 | -0.0202 | -0.0200 | -0.0204 | -0.0201 | -0.0199 | -0.0203 | -0.0194 | -0.0197 | -0.0199 |
| 0.6 | -0.0220 | -0.0217 | -0.0221 | -0.0216 | -0.0213 | -0.0219 | -0.0206 | -0.0208 | -0.0209 |
| 0.65 | -0.0219 | -0.0217 | -0.0218 | -0.0219 | -0.0219 | -0.0219 | -0.0213 | -0.0232 | -0.0220 |
| 0.7 | -0.0172 | -0.0174 | -0.0171 | -0.0174 | -0.0175 | -0.0173 | -0.0176 | -0.0179 | -0.0185 |
| 0.75 | -0.0153 | -0.0152 | -0.0155 | -0.0152 | -0.0150 | -0.0152 | -0.0138 | -0.0146 | -0.0147 |
| 0.8 | -0.0073 | -0.0073 | -0.0073 | -0.0073 | -0.0073 | -0.0073 | -0.0075 | -0.0072 | -0.0071 |
| 0.85 | -0.0148 | -0.0149 | -0.0146 | -0.0149 | -0.0151 | -0.0148 | -0.0166 | -0.0159 | -0.0136 |
| 0.9 | -0.0197 | -0.0193 | -0.0193 | -0.0193 | -0.0193 | -0.0193 | -0.0196 | -0.0173 | -0.0183 |
| 0.95 | -0.0208 | -0.0207 | -0.0210 | -0.0204 | -0.0200 | -0.0204 | -0.0201 | -0.0190 | -0.0221 |
| women |  |  |  |  |  |  |  |  |  |
| 0.05 | -0.0174 | -0.0177 | -0.0173 | -0.0175 | -0.0177 | -0.0175 | -0.0189 | -0.0187 | -0.0196 |
| 0.1 | -0.0276 | -0.0275 | -0.0277 | -0.0275 | -0.0274 | -0.0276 | -0.0282 | -0.0259 | -0.0262 |
| 0.15 | -0.0322 | -0.0318 | -0.0323 | -0.0321 | -0.0319 | -0.0323 | -0.0316 | -0.0307 | -0.0312 |
| 0.2 | -0.0444 | -0.0441 | -0.0445 | -0.0443 | -0.0439 | -0.0444 | -0.0438 | -0.0447 | -0.0428 |
| 0.25 | -0.0521 | -0.0518 | -0.0521 | -0.0520 | -0.0519 | -0.0522 | -0.0533 | -0.0530 | -0.0519 |
| 0.3 | -0.0612 | -0.0611 | -0.0614 | -0.0615 | -0.0614 | -0.0615 | -0.0611 | -0.0597 | -0.0640 |
| 0.35 | -0.0642 | -0.0641 | -0.0645 | -0.0643 | -0.0640 | -0.0645 | -0.0624 | -0.0620 | -0.0627 |
| 0.4 | -0.0702 | -0.0694 | -0.0704 | -0.0698 | -0.0692 | -0.0700 | -0.0716 | -0.0702 | -0.0696 |
| 0.45 | -0.0827 | -0.0824 | -0.0830 | -0.0825 | -0.0821 | -0.0827 | -0.0823 | -0.0809 | -0.0812 |
| 0.5 | -0.0920 | -0.0927 | -0.0921 | -0.0927 | -0.0931 | -0.0926 | -0.0914 | -0.0900 | -0.0947 |
| 0.55 | -0.0950 | -0.0952 | -0.0947 | -0.0954 | -0.0959 | -0.0953 | -0.1012 | -0.1007 | -0.0959 |
| 0.6 | -0.1027 | -0.1021 | -0.1030 | -0.1020 | -0.1014 | -0.1022 | -0.0972 | -0.1031 | -0.1003 |
| 0.65 | -0.1000 | -0.0992 | -0.1003 | -0.0997 | -0.0991 | -0.0999 | -0.1002 | -0.0978 | -0.0993 |
| 0.7 | -0.1021 | -0.1029 | -0.1023 | -0.1033 | -0.1034 | -0.1033 | -0.0979 | -0.1115 | -0.1017 |
| 0.75 | -0.1096 | -0.1087 | -0.1099 | -0.1092 | -0.1087 | -0.1094 | -0.1064 | -0.1124 | -0.1077 |
| 0.8 | -0.1143 | -0.1132 | -0.1145 | -0.1133 | -0.1125 | -0.1136 | -0.1129 | -0.1160 | -0.1142 |
| 0.85 | -0.1258 | -0.1255 | -0.1259 | -0.1264 | -0.1271 | -0.1261 | -0.1416 | -0.1319 | -0.1353 |
| 0.9 | -0.1397 | -0.1422 | -0.1392 | -0.1417 | -0.1430 | -0.1412 | -0.1478 | -0.1411 | -0.1418 |
| 0.95 | -0.1563 | -0.1571 | -0.1561 | -0.1591 | -0.1614 | -0.1610 | -0.1372 | -0.1677 | -0.1475 |
| Universitary |  |  |  |  |  |  |  |  |  |
| men |  |  |  |  |  |  |  |  |  |
| 0.05 | 0.0075 | 0.0075 | 0.0074 | 0.0074 | 0.0074 | 0.0074 | 0.0070 | 0.0070 | 0.0074 |
| 0.1 | 0.0173 | 0.0176 | 0.0173 | 0.0174 | 0.0174 | 0.0173 | 0.0179 | 0.0180 | 0.0177 |
| 0.15 | 0.0171 | 0.0172 | 0.0171 | 0.0171 | 0.0172 | 0.0170 | 0.0172 | 0.0172 | 0.0176 |
| 0.2 | 0.0078 | 0.0078 | 0.0078 | 0.0078 | 0.0079 | 0.0078 | 0.0080 | 0.0075 | 0.0082 |
| 0.25 | -0.0008 | -0.0008 | -0.0008 | -0.0008 | -0.0008 | -0.0008 | -0.0008 | -0.0008 | -0.0008 |
| 0.3 | -0.0050 | -0.0050 | -0.0050 | -0.0050 | -0.0050 | -0.0050 | -0.0047 | -0.0050 | -0.0050 |
| 0.35 | -0.0145 | -0.0144 | -0.0146 | -0.0146 | -0.0145 | -0.0146 | -0.0146 | -0.0148 | -0.0149 |
| 0.4 | -0.0201 | -0.0199 | -0.0201 | -0.0198 | -0.0197 | -0.0200 | -0.0188 | -0.0186 | -0.0194 |
| 0.45 | -0.0273 | -0.0272 | -0.0273 | -0.0273 | -0.0272 | -0.0274 | -0.0274 | -0.0260 | -0.0274 |
| 0.5 | -0.0273 | -0.0272 | -0.0273 | -0.0276 | -0.0276 | -0.0276 | -0.0267 | -0.0281 | -0.0278 |
| 0.55 | -0.0350 | -0.0347 | -0.0354 | -0.0348 | -0.0345 | -0.0352 | -0.0336 | -0.0342 | -0.0346 |
| 0.6 | -0.0341 | -0.0336 | -0.0343 | -0.0335 | -0.0330 | -0.0339 | -0.0320 | -0.0322 | -0.0324 |
| 0.65 | -0.0373 | -0.0370 | -0.0371 | -0.0373 | -0.0373 | -0.0374 | -0.0362 | -0.0395 | -0.0375 |
| 0.7 | -0.0346 | -0.0350 | -0.0344 | -0.0350 | -0.0352 | -0.0348 | -0.0354 | -0.0361 | -0.0373 |
| 0.75 | -0.0317 | -0.0314 | -0.0320 | -0.0313 | -0.0310 | -0.0315 | -0.0285 | -0.0302 | -0.0304 |
| 0.8 | -0.0223 | -0.0222 | -0.0221 | -0.0222 | -0.0222 | -0.0222 | -0.0230 | -0.0218 | -0.0216 |
| 0.85 | -0.0300 | -0.0302 | -0.0297 | -0.0303 | -0.0306 | -0.0301 | -0.0337 | -0.0323 | -0.0276 |
| 0.9 | -0.0325 | -0.0319 | -0.0319 | -0.0318 | -0.0318 | -0.0318 | -0.0324 | -0.0286 | -0.0302 |
| 0.95 | -0.0389 | -0.0388 | -0.0393 | -0.0381 | -0.0374 | -0.0374 | -0.0376 | -0.0355 | -0.0414 |
| women |  |  |  |  |  |  |  |  |  |
| 0.05 | -0.0511 | -0.0519 | -0.0508 | -0.0514 | -0.0521 | -0.0515 | -0.0556 | -0.0549 | -0.0577 |
| 0.1 | -0.0663 | -0.0663 | -0.0666 | -0.0662 | -0.0659 | -0.0664 | -0.0678 | -0.0623 | -0.0629 |
| 0.15 | -0.0738 | -0.0730 | -0.0741 | -0.0737 | -0.0731 | -0.0741 | -0.0725 | -0.0705 | -0.0715 |
| 0.2 | -0.0875 | -0.0868 | -0.0877 | -0.0872 | -0.0866 | -0.0876 | -0.0862 | -0.0882 | -0.0844 |
| 0.25 | -0.0942 | -0.0937 | -0.0942 | -0.0941 | -0.0938 | -0.0943 | -0.0964 | -0.0958 | -0.0938 |
| 0.3 | -0.1015 | -0.1014 | -0.1019 | -0.1020 | -0.1019 | -0.1022 | -0.1014 | -0.0991 | -0.1063 |
| 0.35 | -0.1150 | -0.1149 | -0.1156 | -0.1152 | -0.1146 | -0.1156 | -0.1118 | -0.1111 | -0.1123 |
| 0.4 | -0.1207 | -0.1193 | -0.1211 | -0.1200 | -0.1190 | -0.1204 | -0.1231 | -0.1207 | -0.1196 |
| 0.45 | -0.1318 | -0.1313 | -0.1321 | -0.1314 | -0.1307 | -0.1317 | -0.1311 | -0.1288 | -0.1293 |
| 0.5 | -0.1326 | -0.1337 | -0.1327 | -0.1336 | -0.1342 | -0.1335 | -0.1318 | -0.1297 | -0.1365 |
| 0.55 | -0.1339 | -0.1342 | -0.1335 | -0.1345 | -0.1351 | -0.1343 | -0.1426 | -0.1420 | -0.1352 |
| 0.6 | -0.1354 | -0.1346 | -0.1358 | -0.1345 | -0.1336 | -0.1348 | -0.1282 | -0.1360 | -0.1322 |
| 0.65 | -0.1254 | -0.1244 | -0.1259 | -0.1250 | -0.1243 | -0.1254 | -0.1257 | -0.1228 | -0.1246 |
| 0.7 | -0.1271 | -0.1281 | -0.1274 | -0.1286 | -0.1287 | -0.1287 | -0.1219 | -0.1388 | -0.1267 |
| 0.75 | -0.1329 | -0.1318 | -0.1333 | -0.1324 | -0.1318 | -0.1324 | -0.1290 | -0.1362 | -0.1306 |
| 0.8 | -0.1380 | -0.1367 | -0.1383 | -0.1368 | -0.1359 | -0.1372 | -0.1363 | -0.1401 | -0.1380 |
| 0.85 | -0.1478 | -0.1475 | -0.1479 | -0.1486 | -0.1494 | -0.1482 | -0.1665 | -0.1551 | -0.1590 |
| 0.9 | -0.1594 | -0.1623 | -0.1589 | -0.1617 | -0.1632 | -0.1611 | -0.1687 | -0.1610 | -0.1618 |
| 0.95 | -0.1651 | -0.1660 | -0.1650 | -0.1682 | -0.1706 | -0.1713 | -0.1450 | -0.1772 | -0.1559 |
| 2006 |  |  |  |  |  |  |  |  |  |
| Income 25 |  |  |  |  |  |  |  |  |  |
| men |  |  |  |  |  |  |  |  |  |
| 0.05 | 0.0171 | 0.0172 | 0.0169 | 0.0170 | 0.0171 | 0.0169 | 0.0164 | 0.0169 | 0.0171 |
| 0.1 | 0.0086 | 0.0086 | 0.0086 | 0.0086 | 0.0086 | 0.0085 | 0.0087 | 0.0087 | 0.0088 |
| 0.15 | 0.0148 | 0.0148 | 0.0149 | 0.0148 | 0.0149 | 0.0148 | 0.0149 | 0.0149 | 0.0146 |
| 0.2 | 0.0066 | 0.0066 | 0.0066 | 0.0066 | 0.0066 | 0.0066 | 0.0068 | 0.0065 | 0.0068 |
| 0.25 | 0.0068 | 0.0067 | 0.0069 | 0.0068 | 0.0068 | 0.0069 | 0.0067 | 0.0071 | 0.0068 |
| 0.3 | 0.0081 | 0.0081 | 0.0082 | 0.0080 | 0.0080 | 0.0081 | 0.0078 | 0.0079 | 0.0078 |
| 0.35 | 0.0054 | 0.0054 | 0.0055 | 0.0054 | 0.0054 | 0.0055 | 0.0054 | 0.0055 | 0.0052 |
| 0.4 | 0.0018 | 0.0018 | 0.0018 | 0.0018 | 0.0018 | 0.0018 | 0.0019 | 0.0019 | 0.0018 |
| 0.45 | -0.0040 | -0.0040 | -0.0040 | -0.0040 | -0.0040 | -0.0040 | -0.0038 | -0.0038 | -0.0040 |
| 0.5 | -0.0017 | -0.0017 | -0.0017 | -0.0017 | -0.0017 | -0.0017 | -0.0017 | -0.0017 | -0.0017 |
| 0.55 | -0.0015 | -0.0015 | -0.0015 | -0.0015 | -0.0015 | -0.0015 | -0.0015 | -0.0014 | -0.0015 |
| 0.6 | -0.0009 | -0.0009 | -0.0009 | -0.0009 | -0.0009 | -0.0009 | -0.0009 | -0.0010 | -0.0008 |
| 0.65 | 0.0034 | 0.0033 | 0.0034 | 0.0033 | 0.0033 | 0.0034 | 0.0034 | 0.0033 | 0.0032 |
| 0.7 | 0.0027 | 0.0027 | 0.0027 | 0.0027 | 0.0027 | 0.0027 | 0.0026 | 0.0027 | 0.0025 |
| 0.75 | 0.0009 | 0.0009 | 0.0009 | 0.0009 | 0.0009 | 0.0009 | 0.0011 | 0.0011 | 0.0009 |
| 0.8 | -0.0046 | -0.0045 | -0.0046 | -0.0045 | -0.0045 | -0.0045 | -0.0050 | -0.0045 | -0.0042 |
| 0.85 | 0.0027 | 0.0027 | 0.0027 | 0.0028 | 0.0028 | 0.0027 | 0.0028 | 0.0030 | 0.0030 |
| 0.9 | 0.0050 | 0.0050 | 0.0050 | 0.0050 | 0.0050 | 0.0049 | 0.0047 | 0.0051 | 0.0049 |
| 0.95 | 0.0034 | 0.0034 | 0.0034 | 0.0034 | 0.0035 | 0.0034 | 0.0034 | 0.0034 | 0.0033 |
| women |  |  |  |  |  |  |  |  |  |
| 0.05 | 0.0027 | 0.0027 | 0.0027 | 0.0027 | 0.0027 | 0.0027 | 0.0027 | 0.0028 | 0.0028 |
| 0.1 | -0.0047 | -0.0046 | -0.0047 | -0.0047 | -0.0046 | -0.0047 | -0.0048 | -0.0048 | -0.0048 |
| 0.15 | -0.0013 | -0.0013 | -0.0013 | -0.0013 | -0.0013 | -0.0013 | -0.0013 | -0.0013 | -0.0013 |
| 0.2 | -0.0109 | -0.0109 | -0.0110 | -0.0109 | -0.0109 | -0.0110 | -0.0111 | -0.0113 | -0.0112 |
| 0.25 | -0.0066 | -0.0066 | -0.0066 | -0.0066 | -0.0066 | -0.0066 | -0.0066 | -0.0068 | -0.0065 |
| 0.3 | -0.0026 | -0.0026 | -0.0026 | -0.0026 | -0.0026 | -0.0026 | -0.0026 | -0.0026 | -0.0026 |
| 0.35 | 0.0018 | 0.0018 | 0.0018 | 0.0018 | 0.0018 | 0.0018 | 0.0018 | 0.0017 | 0.0018 |
| 0.4 | -0.0017 | -0.0017 | -0.0017 | -0.0017 | -0.0017 | -0.0017 | -0.0017 | -0.0017 | -0.0017 |
| 0.45 | 0.0005 | 0.0005 | 0.0005 | 0.0005 | 0.0005 | 0.0005 | 0.0005 | 0.0005 | 0.0005 |
| 0.5 | -0.0062 | -0.0062 | -0.0062 | -0.0062 | -0.0062 | -0.0062 | -0.0062 | -0.0062 | -0.0063 |
| 0.55 | -0.0043 | -0.0043 | -0.0043 | -0.0043 | -0.0043 | -0.0043 | -0.0044 | -0.0041 | -0.0043 |
| 0.6 | -0.0077 | -0.0077 | -0.0078 | -0.0077 | -0.0077 | -0.0077 | -0.0079 | -0.0076 | -0.0077 |
| 0.65 | -0.0059 | -0.0059 | -0.0059 | -0.0059 | -0.0059 | -0.0059 | -0.0060 | -0.0059 | -0.0062 |
| 0.7 | -0.0149 | -0.0149 | -0.0149 | -0.0149 | -0.0149 | -0.0149 | -0.0147 | -0.0148 | -0.0147 |
| 0.75 | -0.0132 | -0.0133 | -0.0132 | -0.0133 | -0.0134 | -0.0132 | -0.0132 | -0.0142 | -0.0137 |
| 0.8 | -0.0123 | -0.0123 | -0.0123 | -0.0122 | -0.0122 | -0.0122 | -0.0119 | -0.0124 | -0.0122 |
| 0.85 | -0.0154 | -0.0153 | -0.0153 | -0.0155 | -0.0155 | -0.0155 | -0.0149 | -0.0176 | -0.0157 |
| 0.9 | -0.0243 | -0.0244 | -0.0243 | -0.0241 | -0.0239 | -0.0241 | -0.0217 | -0.0250 | -0.0232 |
| 0.95 | -0.0394 | -0.0391 | -0.0392 | -0.0393 | -0.0396 | -0.0392 | -0.0385 | -0.0394 | -0.0399 |
| Income 50 |  |  |  |  |  |  |  |  |  |
| men |  |  |  |  |  |  |  |  |  |
| 0.05 | 0.0348 | 0.0349 | 0.0343 | 0.0345 | 0.0347 | 0.0345 | 0.0332 | 0.0343 | 0.0346 |
| 0.1 | 0.0113 | 0.0113 | 0.0113 | 0.0113 | 0.0113 | 0.0112 | 0.0114 | 0.0115 | 0.0116 |
| 0.15 | 0.0092 | 0.0092 | 0.0092 | 0.0092 | 0.0092 | 0.0092 | 0.0093 | 0.0092 | 0.0091 |
| 0.2 | 0.0059 | 0.0059 | 0.0059 | 0.0059 | 0.0059 | 0.0059 | 0.0061 | 0.0058 | 0.0061 |
| 0.25 | 0.0090 | 0.0089 | 0.0091 | 0.0090 | 0.0090 | 0.0091 | 0.0089 | 0.0094 | 0.0089 |
| 0.3 | 0.0075 | 0.0075 | 0.0076 | 0.0075 | 0.0074 | 0.0075 | 0.0073 | 0.0073 | 0.0072 |
| 0.35 | 0.0026 | 0.0025 | 0.0026 | 0.0026 | 0.0025 | 0.0026 | 0.0025 | 0.0026 | 0.0024 |
| 0.4 | 0.0004 | 0.0004 | 0.0004 | 0.0004 | 0.0004 | 0.0004 | 0.0004 | 0.0004 | 0.0004 |
| 0.45 | 0.0006 | 0.0006 | 0.0006 | 0.0006 | 0.0006 | 0.0006 | 0.0006 | 0.0006 | 0.0006 |
| 0.5 | 0.0041 | 0.0041 | 0.0041 | 0.0041 | 0.0040 | 0.0041 | 0.0040 | 0.0039 | 0.0040 |
| 0.55 | 0.0007 | 0.0007 | 0.0007 | 0.0007 | 0.0007 | 0.0007 | 0.0007 | 0.0007 | 0.0007 |
| 0.6 | 0.0000 | 0.0000 | 0.0000 | 0.0000 | 0.0000 | 0.0000 | 0.0000 | 0.0000 | 0.0000 |
| 0.65 | 0.0035 | 0.0035 | 0.0036 | 0.0035 | 0.0034 | 0.0035 | 0.0036 | 0.0034 | 0.0034 |
| 0.7 | 0.0028 | 0.0028 | 0.0028 | 0.0028 | 0.0028 | 0.0028 | 0.0027 | 0.0028 | 0.0026 |
| 0.75 | -0.0004 | -0.0004 | -0.0004 | -0.0004 | -0.0004 | -0.0004 | -0.0005 | -0.0005 | -0.0004 |
| 0.8 | -0.0013 | -0.0013 | -0.0013 | -0.0013 | -0.0013 | -0.0013 | -0.0014 | -0.0013 | -0.0012 |
| 0.85 | 0.0021 | 0.0021 | 0.0021 | 0.0022 | 0.0022 | 0.0021 | 0.0022 | 0.0024 | 0.0024 |
| 0.9 | -0.0005 | -0.0005 | -0.0005 | -0.0005 | -0.0005 | -0.0005 | -0.0004 | -0.0005 | -0.0005 |
| 0.95 | -0.0020 | -0.0020 | -0.0020 | -0.0020 | -0.0020 | -0.0020 | -0.0020 | -0.0020 | -0.0019 |
| women |  |  |  |  |  |  |  |  |  |
| 0.05 | -0.0073 | -0.0073 | -0.0072 | -0.0073 | -0.0074 | -0.0073 | -0.0071 | -0.0074 | -0.0074 |
| 0.1 | -0.0057 | -0.0056 | -0.0057 | -0.0057 | -0.0056 | -0.0057 | -0.0058 | -0.0059 | -0.0058 |
| 0.15 | -0.0043 | -0.0043 | -0.0043 | -0.0043 | -0.0043 | -0.0043 | -0.0042 | -0.0043 | -0.0041 |
| 0.2 | -0.0169 | -0.0169 | -0.0170 | -0.0169 | -0.0169 | -0.0170 | -0.0171 | -0.0174 | -0.0174 |
| 0.25 | -0.0125 | -0.0124 | -0.0125 | -0.0125 | -0.0124 | -0.0125 | -0.0124 | -0.0129 | -0.0123 |
| 0.3 | -0.0101 | -0.0101 | -0.0101 | -0.0101 | -0.0101 | -0.0101 | -0.0101 | -0.0099 | -0.0100 |
| 0.35 | -0.0123 | -0.0123 | -0.0123 | -0.0123 | -0.0123 | -0.0124 | -0.0123 | -0.0122 | -0.0122 |
| 0.4 | -0.0153 | -0.0153 | -0.0153 | -0.0153 | -0.0152 | -0.0153 | -0.0148 | -0.0153 | -0.0151 |
| 0.45 | -0.0136 | -0.0135 | -0.0136 | -0.0136 | -0.0136 | -0.0136 | -0.0138 | -0.0140 | -0.0140 |
| 0.5 | -0.0167 | -0.0167 | -0.0167 | -0.0167 | -0.0167 | -0.0168 | -0.0167 | -0.0167 | -0.0169 |
| 0.55 | -0.0158 | -0.0157 | -0.0158 | -0.0158 | -0.0157 | -0.0158 | -0.0159 | -0.0148 | -0.0156 |
| 0.6 | -0.0213 | -0.0211 | -0.0214 | -0.0212 | -0.0211 | -0.0213 | -0.0216 | -0.0208 | -0.0213 |
| 0.65 | -0.0238 | -0.0236 | -0.0238 | -0.0238 | -0.0238 | -0.0239 | -0.0240 | -0.0238 | -0.0249 |
| 0.7 | -0.0328 | -0.0328 | -0.0328 | -0.0327 | -0.0326 | -0.0327 | -0.0324 | -0.0324 | -0.0322 |
| 0.75 | -0.0291 | -0.0293 | -0.0290 | -0.0293 | -0.0295 | -0.0292 | -0.0291 | -0.0313 | -0.0303 |
| 0.8 | -0.0339 | -0.0338 | -0.0338 | -0.0336 | -0.0335 | -0.0336 | -0.0327 | -0.0340 | -0.0334 |
| 0.85 | -0.0445 | -0.0444 | -0.0444 | -0.0448 | -0.0449 | -0.0448 | -0.0430 | -0.0509 | -0.0456 |
| 0.9 | -0.0480 | -0.0481 | -0.0479 | -0.0475 | -0.0471 | -0.0476 | -0.0428 | -0.0493 | -0.0458 |
| 0.95 | -0.0684 | -0.0678 | -0.0680 | -0.0682 | -0.0688 | -0.0685 | -0.0668 | -0.0683 | -0.0692 |
| Income 75 |  |  |  |  |  |  |  |  |  |
| men |  |  |  |  |  |  |  |  |  |
| 0.05 | 0.0373 | 0.0375 | 0.0369 | 0.0371 | 0.0372 | 0.0368 | 0.0356 | 0.0368 | 0.0371 |
| 0.1 | 0.0147 | 0.0147 | 0.0147 | 0.0146 | 0.0147 | 0.0146 | 0.0149 | 0.0149 | 0.0150 |
| 0.15 | 0.0111 | 0.0111 | 0.0112 | 0.0111 | 0.0111 | 0.0111 | 0.0112 | 0.0111 | 0.0110 |
| 0.2 | 0.0060 | 0.0060 | 0.0060 | 0.0060 | 0.0060 | 0.0060 | 0.0061 | 0.0059 | 0.0061 |
| 0.25 | 0.0081 | 0.0080 | 0.0081 | 0.0081 | 0.0080 | 0.0081 | 0.0079 | 0.0084 | 0.0080 |
| 0.3 | 0.0069 | 0.0068 | 0.0069 | 0.0068 | 0.0068 | 0.0069 | 0.0067 | 0.0067 | 0.0066 |
| 0.35 | 0.0005 | 0.0005 | 0.0005 | 0.0005 | 0.0005 | 0.0005 | 0.0005 | 0.0005 | 0.0005 |
| 0.4 | -0.0037 | -0.0037 | -0.0037 | -0.0037 | -0.0037 | -0.0037 | -0.0038 | -0.0039 | -0.0037 |
| 0.45 | -0.0055 | -0.0055 | -0.0055 | -0.0055 | -0.0054 | -0.0055 | -0.0053 | -0.0052 | -0.0054 |
| 0.5 | -0.0046 | -0.0046 | -0.0047 | -0.0046 | -0.0046 | -0.0046 | -0.0045 | -0.0044 | -0.0045 |
| 0.55 | -0.0072 | -0.0073 | -0.0071 | -0.0072 | -0.0072 | -0.0072 | -0.0071 | -0.0069 | -0.0072 |
| 0.6 | -0.0097 | -0.0097 | -0.0098 | -0.0098 | -0.0098 | -0.0098 | -0.0099 | -0.0107 | -0.0091 |
| 0.65 | -0.0065 | -0.0063 | -0.0065 | -0.0064 | -0.0063 | -0.0064 | -0.0065 | -0.0063 | -0.0061 |
| 0.7 | -0.0068 | -0.0068 | -0.0068 | -0.0068 | -0.0067 | -0.0068 | -0.0067 | -0.0069 | -0.0064 |
| 0.75 | -0.0107 | -0.0107 | -0.0107 | -0.0108 | -0.0109 | -0.0108 | -0.0125 | -0.0121 | -0.0099 |
| 0.8 | -0.0123 | -0.0121 | -0.0125 | -0.0121 | -0.0119 | -0.0122 | -0.0134 | -0.0122 | -0.0112 |
| 0.85 | -0.0119 | -0.0121 | -0.0119 | -0.0122 | -0.0123 | -0.0121 | -0.0126 | -0.0134 | -0.0133 |
| 0.9 | -0.0171 | -0.0172 | -0.0171 | -0.0170 | -0.0171 | -0.0170 | -0.0160 | -0.0176 | -0.0169 |
| 0.95 | -0.0256 | -0.0256 | -0.0256 | -0.0257 | -0.0257 | -0.0256 | -0.0251 | -0.0254 | -0.0246 |
| women |  |  |  |  |  |  |  |  |  |
| 0.05 | -0.0050 | -0.0051 | -0.0050 | -0.0051 | -0.0051 | -0.0051 | -0.0050 | -0.0052 | -0.0052 |
| 0.1 | -0.0112 | -0.0111 | -0.0112 | -0.0111 | -0.0111 | -0.0112 | -0.0114 | -0.0116 | -0.0114 |
| 0.15 | -0.0137 | -0.0136 | -0.0138 | -0.0137 | -0.0137 | -0.0137 | -0.0133 | -0.0137 | -0.0133 |
| 0.2 | -0.0269 | -0.0269 | -0.0269 | -0.0269 | -0.0268 | -0.0269 | -0.0272 | -0.0276 | -0.0276 |
| 0.25 | -0.0276 | -0.0274 | -0.0276 | -0.0275 | -0.0275 | -0.0274 | -0.0274 | -0.0284 | -0.0272 |
| 0.3 | -0.0269 | -0.0269 | -0.0270 | -0.0269 | -0.0268 | -0.0269 | -0.0269 | -0.0265 | -0.0266 |
| 0.35 | -0.0275 | -0.0274 | -0.0276 | -0.0276 | -0.0275 | -0.0276 | -0.0274 | -0.0273 | -0.0273 |
| 0.4 | -0.0301 | -0.0301 | -0.0301 | -0.0301 | -0.0300 | -0.0301 | -0.0291 | -0.0301 | -0.0298 |
| 0.45 | -0.0298 | -0.0297 | -0.0298 | -0.0298 | -0.0297 | -0.0298 | -0.0302 | -0.0306 | -0.0306 |
| 0.5 | -0.0339 | -0.0339 | -0.0340 | -0.0340 | -0.0339 | -0.0341 | -0.0339 | -0.0338 | -0.0342 |
| 0.55 | -0.0374 | -0.0373 | -0.0375 | -0.0373 | -0.0371 | -0.0375 | -0.0377 | -0.0351 | -0.0368 |
| 0.6 | -0.0393 | -0.0390 | -0.0395 | -0.0392 | -0.0389 | -0.0394 | -0.0399 | -0.0385 | -0.0393 |
| 0.65 | -0.0421 | -0.0418 | -0.0423 | -0.0422 | -0.0421 | -0.0423 | -0.0425 | -0.0422 | -0.0442 |
| 0.7 | -0.0520 | -0.0520 | -0.0519 | -0.0517 | -0.0516 | -0.0518 | -0.0512 | -0.0513 | -0.0510 |
| 0.75 | -0.0513 | -0.0517 | -0.0511 | -0.0516 | -0.0520 | -0.0515 | -0.0512 | -0.0551 | -0.0533 |
| 0.8 | -0.0509 | -0.0508 | -0.0508 | -0.0505 | -0.0504 | -0.0505 | -0.0491 | -0.0511 | -0.0502 |
| 0.85 | -0.0587 | -0.0586 | -0.0586 | -0.0592 | -0.0593 | -0.0592 | -0.0567 | -0.0672 | -0.0601 |
| 0.9 | -0.0596 | -0.0596 | -0.0594 | -0.0589 | -0.0584 | -0.0591 | -0.0530 | -0.0611 | -0.0568 |
| 0.95 | -0.0753 | -0.0747 | -0.0749 | -0.0752 | -0.0757 | -0.0759 | -0.0736 | -0.0752 | -0.0762 |
| Income 90 |  |  |  |  |  |  |  |  |  |
| men |  |  |  |  |  |  |  |  |  |
| 0.05 | 0.0537 | 0.0539 | 0.0530 | 0.0533 | 0.0535 | 0.0535 | 0.0513 | 0.0529 | 0.0534 |
| 0.1 | 0.0251 | 0.0252 | 0.0251 | 0.0250 | 0.0251 | 0.0249 | 0.0254 | 0.0255 | 0.0256 |
| 0.15 | 0.0212 | 0.0212 | 0.0214 | 0.0213 | 0.0213 | 0.0213 | 0.0215 | 0.0214 | 0.0210 |
| 0.2 | 0.0099 | 0.0099 | 0.0099 | 0.0099 | 0.0099 | 0.0099 | 0.0102 | 0.0097 | 0.0102 |
| 0.25 | 0.0055 | 0.0054 | 0.0056 | 0.0055 | 0.0055 | 0.0055 | 0.0054 | 0.0057 | 0.0054 |
| 0.3 | 0.0004 | 0.0004 | 0.0004 | 0.0004 | 0.0004 | 0.0004 | 0.0004 | 0.0004 | 0.0004 |
| 0.35 | -0.0060 | -0.0059 | -0.0060 | -0.0060 | -0.0059 | -0.0060 | -0.0059 | -0.0061 | -0.0057 |
| 0.4 | -0.0107 | -0.0108 | -0.0108 | -0.0108 | -0.0109 | -0.0109 | -0.0112 | -0.0114 | -0.0109 |
| 0.45 | -0.0105 | -0.0104 | -0.0105 | -0.0104 | -0.0103 | -0.0105 | -0.0100 | -0.0099 | -0.0104 |
| 0.5 | -0.0123 | -0.0121 | -0.0124 | -0.0122 | -0.0121 | -0.0122 | -0.0120 | -0.0117 | -0.0120 |
| 0.55 | -0.0137 | -0.0139 | -0.0137 | -0.0139 | -0.0139 | -0.0139 | -0.0137 | -0.0133 | -0.0138 |
| 0.6 | -0.0154 | -0.0154 | -0.0155 | -0.0156 | -0.0156 | -0.0156 | -0.0158 | -0.0171 | -0.0145 |
| 0.65 | -0.0110 | -0.0109 | -0.0112 | -0.0109 | -0.0107 | -0.0110 | -0.0112 | -0.0107 | -0.0105 |
| 0.7 | -0.0155 | -0.0155 | -0.0156 | -0.0155 | -0.0154 | -0.0155 | -0.0153 | -0.0157 | -0.0147 |
| 0.75 | -0.0176 | -0.0177 | -0.0177 | -0.0179 | -0.0180 | -0.0179 | -0.0207 | -0.0201 | -0.0164 |
| 0.8 | -0.0228 | -0.0223 | -0.0231 | -0.0224 | -0.0221 | -0.0226 | -0.0249 | -0.0225 | -0.0208 |
| 0.85 | -0.0248 | -0.0251 | -0.0249 | -0.0254 | -0.0257 | -0.0252 | -0.0262 | -0.0279 | -0.0277 |
| 0.9 | -0.0307 | -0.0309 | -0.0307 | -0.0306 | -0.0306 | -0.0304 | -0.0287 | -0.0315 | -0.0303 |
| 0.95 | -0.0449 | -0.0449 | -0.0448 | -0.0449 | -0.0450 | -0.0449 | -0.0440 | -0.0445 | -0.0431 |
| women |  |  |  |  |  |  |  |  |  |
| 0.05 | -0.0173 | -0.0174 | -0.0172 | -0.0174 | -0.0176 | -0.0174 | -0.0170 | -0.0177 | -0.0177 |
| 0.1 | -0.0248 | -0.0246 | -0.0250 | -0.0247 | -0.0245 | -0.0248 | -0.0253 | -0.0256 | -0.0254 |
| 0.15 | -0.0349 | -0.0346 | -0.0350 | -0.0349 | -0.0348 | -0.0349 | -0.0338 | -0.0349 | -0.0337 |
| 0.2 | -0.0506 | -0.0506 | -0.0507 | -0.0506 | -0.0505 | -0.0507 | -0.0512 | -0.0520 | -0.0519 |
| 0.25 | -0.0589 | -0.0584 | -0.0588 | -0.0587 | -0.0585 | -0.0588 | -0.0583 | -0.0605 | -0.0580 |
| 0.3 | -0.0631 | -0.0631 | -0.0633 | -0.0631 | -0.0629 | -0.0632 | -0.0631 | -0.0621 | -0.0625 |
| 0.35 | -0.0628 | -0.0626 | -0.0630 | -0.0630 | -0.0629 | -0.0631 | -0.0626 | -0.0623 | -0.0624 |
| 0.4 | -0.0671 | -0.0671 | -0.0671 | -0.0670 | -0.0669 | -0.0671 | -0.0649 | -0.0671 | -0.0664 |
| 0.45 | -0.0663 | -0.0662 | -0.0663 | -0.0663 | -0.0663 | -0.0664 | -0.0673 | -0.0683 | -0.0683 |
| 0.5 | -0.0711 | -0.0711 | -0.0712 | -0.0713 | -0.0711 | -0.0714 | -0.0711 | -0.0709 | -0.0718 |
| 0.55 | -0.0676 | -0.0674 | -0.0679 | -0.0675 | -0.0671 | -0.0678 | -0.0682 | -0.0635 | -0.0666 |
| 0.6 | -0.0702 | -0.0695 | -0.0705 | -0.0699 | -0.0694 | -0.0702 | -0.0711 | -0.0687 | -0.0701 |
| 0.65 | -0.0741 | -0.0736 | -0.0743 | -0.0743 | -0.0741 | -0.0744 | -0.0748 | -0.0743 | -0.0777 |
| 0.7 | -0.0855 | -0.0855 | -0.0854 | -0.0851 | -0.0849 | -0.0851 | -0.0842 | -0.0844 | -0.0839 |
| 0.75 | -0.0858 | -0.0865 | -0.0855 | -0.0863 | -0.0870 | -0.0865 | -0.0857 | -0.0922 | -0.0892 |
| 0.8 | -0.0819 | -0.0817 | -0.0817 | -0.0813 | -0.0811 | -0.0813 | -0.0791 | -0.0823 | -0.0809 |
| 0.85 | -0.0928 | -0.0926 | -0.0926 | -0.0936 | -0.0937 | -0.0936 | -0.0897 | -0.1062 | -0.0951 |
| 0.9 | -0.0886 | -0.0887 | -0.0884 | -0.0876 | -0.0869 | -0.0879 | -0.0788 | -0.0908 | -0.0845 |
| 0.95 | -0.1113 | -0.1104 | -0.1107 | -0.1110 | -0.1119 | -0.1116 | -0.1087 | -0.1111 | -0.1125 |
| Income 100 |  |  |  |  |  |  |  |  |  |
| men |  |  |  |  |  |  |  |  |  |
| 0.05 | 0.0517 | 0.0519 | 0.0510 | 0.0513 | 0.0515 | 0.0515 | 0.0493 | 0.0509 | 0.0514 |
| 0.1 | 0.0147 | 0.0147 | 0.0146 | 0.0146 | 0.0146 | 0.0145 | 0.0148 | 0.0149 | 0.0150 |
| 0.15 | 0.0088 | 0.0088 | 0.0089 | 0.0088 | 0.0089 | 0.0088 | 0.0089 | 0.0089 | 0.0087 |
| 0.2 | -0.0018 | -0.0018 | -0.0018 | -0.0018 | -0.0018 | -0.0018 | -0.0019 | -0.0018 | -0.0018 |
| 0.25 | -0.0009 | -0.0009 | -0.0010 | -0.0009 | -0.0009 | -0.0010 | -0.0009 | -0.0010 | -0.0009 |
| 0.3 | -0.0065 | -0.0065 | -0.0066 | -0.0065 | -0.0064 | -0.0065 | -0.0063 | -0.0063 | -0.0063 |
| 0.35 | -0.0177 | -0.0176 | -0.0178 | -0.0177 | -0.0176 | -0.0177 | -0.0176 | -0.0180 | -0.0168 |
| 0.4 | -0.0244 | -0.0244 | -0.0244 | -0.0246 | -0.0247 | -0.0246 | -0.0254 | -0.0258 | -0.0247 |
| 0.45 | -0.0304 | -0.0302 | -0.0304 | -0.0302 | -0.0300 | -0.0303 | -0.0291 | -0.0288 | -0.0300 |
| 0.5 | -0.0290 | -0.0286 | -0.0293 | -0.0288 | -0.0286 | -0.0289 | -0.0283 | -0.0278 | -0.0284 |
| 0.55 | -0.0311 | -0.0315 | -0.0310 | -0.0314 | -0.0315 | -0.0314 | -0.0309 | -0.0300 | -0.0312 |
| 0.6 | -0.0319 | -0.0319 | -0.0321 | -0.0321 | -0.0322 | -0.0322 | -0.0326 | -0.0352 | -0.0300 |
| 0.65 | -0.0281 | -0.0276 | -0.0284 | -0.0277 | -0.0273 | -0.0279 | -0.0284 | -0.0273 | -0.0267 |
| 0.7 | -0.0262 | -0.0263 | -0.0264 | -0.0262 | -0.0261 | -0.0263 | -0.0258 | -0.0266 | -0.0249 |
| 0.75 | -0.0311 | -0.0312 | -0.0312 | -0.0316 | -0.0318 | -0.0315 | -0.0365 | -0.0353 | -0.0290 |
| 0.8 | -0.0346 | -0.0339 | -0.0350 | -0.0340 | -0.0336 | -0.0342 | -0.0378 | -0.0342 | -0.0316 |
| 0.85 | -0.0331 | -0.0336 | -0.0332 | -0.0339 | -0.0344 | -0.0336 | -0.0350 | -0.0373 | -0.0370 |
| 0.9 | -0.0338 | -0.0340 | -0.0338 | -0.0336 | -0.0337 | -0.0335 | -0.0316 | -0.0347 | -0.0334 |
| 0.95 | -0.0391 | -0.0391 | -0.0390 | -0.0391 | -0.0392 | -0.0391 | -0.0383 | -0.0387 | -0.0375 |
| women |  |  |  |  |  |  |  |  |  |
| 0.05 | -0.0157 | -0.0158 | -0.0157 | -0.0158 | -0.0160 | -0.0158 | -0.0154 | -0.0161 | -0.0161 |
| 0.1 | -0.0324 | -0.0321 | -0.0326 | -0.0323 | -0.0320 | -0.0324 | -0.0330 | -0.0335 | -0.0332 |
| 0.15 | -0.0469 | -0.0464 | -0.0469 | -0.0467 | -0.0466 | -0.0468 | -0.0454 | -0.0468 | -0.0452 |
| 0.2 | -0.0662 | -0.0662 | -0.0663 | -0.0662 | -0.0661 | -0.0663 | -0.0670 | -0.0681 | -0.0679 |
| 0.25 | -0.0756 | -0.0750 | -0.0755 | -0.0754 | -0.0752 | -0.0755 | -0.0749 | -0.0777 | -0.0744 |
| 0.3 | -0.0810 | -0.0809 | -0.0812 | -0.0810 | -0.0807 | -0.0811 | -0.0809 | -0.0797 | -0.0802 |
| 0.35 | -0.0831 | -0.0828 | -0.0833 | -0.0833 | -0.0832 | -0.0834 | -0.0828 | -0.0824 | -0.0825 |
| 0.4 | -0.0916 | -0.0916 | -0.0915 | -0.0914 | -0.0912 | -0.0915 | -0.0885 | -0.0915 | -0.0905 |
| 0.45 | -0.0895 | -0.0893 | -0.0895 | -0.0895 | -0.0894 | -0.0896 | -0.0908 | -0.0921 | -0.0921 |
| 0.5 | -0.0905 | -0.0905 | -0.0907 | -0.0908 | -0.0906 | -0.0909 | -0.0906 | -0.0903 | -0.0914 |
| 0.55 | -0.0886 | -0.0884 | -0.0890 | -0.0885 | -0.0879 | -0.0888 | -0.0894 | -0.0833 | -0.0874 |
| 0.6 | -0.0917 | -0.0909 | -0.0922 | -0.0914 | -0.0908 | -0.0918 | -0.0930 | -0.0898 | -0.0916 |
| 0.65 | -0.0926 | -0.0918 | -0.0928 | -0.0928 | -0.0926 | -0.0930 | -0.0934 | -0.0927 | -0.0971 |
| 0.7 | -0.1010 | -0.1010 | -0.1008 | -0.1005 | -0.1003 | -0.1005 | -0.0995 | -0.0997 | -0.0991 |
| 0.75 | -0.0976 | -0.0984 | -0.0973 | -0.0983 | -0.0990 | -0.0989 | -0.0975 | -0.1049 | -0.1015 |
| 0.8 | -0.0962 | -0.0960 | -0.0960 | -0.0955 | -0.0953 | -0.0955 | -0.0929 | -0.0966 | -0.0949 |
| 0.85 | -0.1048 | -0.1046 | -0.1046 | -0.1057 | -0.1059 | -0.1057 | -0.1013 | -0.1200 | -0.1074 |
| 0.9 | -0.1064 | -0.1066 | -0.1062 | -0.1053 | -0.1044 | -0.1055 | -0.0947 | -0.1091 | -0.1015 |
| 0.95 | -0.1274 | -0.1264 | -0.1267 | -0.1271 | -0.1281 | -0.1277 | -0.1245 | -0.1272 | -0.1288 |
| Primary |  |  |  |  |  |  |  |  |  |
| men |  |  |  |  |  |  |  |  |  |
| 0.05 | 0.0076 | 0.0077 | 0.0075 | 0.0076 | 0.0076 | 0.0075 | 0.0073 | 0.0075 | 0.0076 |
| 0.1 | 0.0076 | 0.0076 | 0.0076 | 0.0076 | 0.0076 | 0.0076 | 0.0077 | 0.0078 | 0.0078 |
| 0.15 | 0.0054 | 0.0054 | 0.0055 | 0.0055 | 0.0055 | 0.0055 | 0.0055 | 0.0055 | 0.0054 |
| 0.2 | 0.0063 | 0.0063 | 0.0063 | 0.0063 | 0.0063 | 0.0063 | 0.0065 | 0.0062 | 0.0065 |
| 0.25 | -0.0035 | -0.0035 | -0.0035 | -0.0035 | -0.0035 | -0.0035 | -0.0035 | -0.0037 | -0.0035 |
| 0.3 | -0.0027 | -0.0027 | -0.0028 | -0.0027 | -0.0027 | -0.0027 | -0.0027 | -0.0027 | -0.0026 |
| 0.35 | -0.0045 | -0.0045 | -0.0045 | -0.0045 | -0.0045 | -0.0045 | -0.0045 | -0.0046 | -0.0043 |
| 0.4 | -0.0056 | -0.0057 | -0.0057 | -0.0057 | -0.0057 | -0.0057 | -0.0059 | -0.0060 | -0.0057 |
| 0.45 | 0.0018 | 0.0017 | 0.0018 | 0.0017 | 0.0017 | 0.0017 | 0.0017 | 0.0017 | 0.0017 |
| 0.5 | 0.0055 | 0.0054 | 0.0056 | 0.0055 | 0.0054 | 0.0055 | 0.0054 | 0.0053 | 0.0054 |
| 0.55 | 0.0028 | 0.0028 | 0.0028 | 0.0028 | 0.0028 | 0.0028 | 0.0028 | 0.0027 | 0.0028 |
| 0.6 | 0.0020 | 0.0020 | 0.0020 | 0.0020 | 0.0020 | 0.0020 | 0.0021 | 0.0022 | 0.0019 |
| 0.65 | 0.0000 | 0.0000 | 0.0000 | 0.0000 | 0.0000 | 0.0000 | 0.0000 | 0.0000 | 0.0000 |
| 0.7 | -0.0035 | -0.0035 | -0.0036 | -0.0035 | -0.0035 | -0.0035 | -0.0035 | -0.0036 | -0.0034 |
| 0.75 | 0.0013 | 0.0013 | 0.0013 | 0.0014 | 0.0014 | 0.0014 | 0.0016 | 0.0015 | 0.0013 |
| 0.8 | -0.0004 | -0.0004 | -0.0004 | -0.0004 | -0.0004 | -0.0004 | -0.0004 | -0.0004 | -0.0004 |
| 0.85 | -0.0082 | -0.0084 | -0.0083 | -0.0084 | -0.0086 | -0.0084 | -0.0087 | -0.0093 | -0.0092 |
| 0.9 | -0.0076 | -0.0077 | -0.0077 | -0.0076 | -0.0076 | -0.0076 | -0.0072 | -0.0079 | -0.0076 |
| 0.95 | -0.0170 | -0.0171 | -0.0170 | -0.0171 | -0.0171 | -0.0170 | -0.0167 | -0.0169 | -0.0164 |
| women |  |  |  |  |  |  |  |  |  |
| 0.05 | 0.0018 | 0.0018 | 0.0018 | 0.0018 | 0.0018 | 0.0018 | 0.0018 | 0.0019 | 0.0019 |
| 0.1 | 0.0064 | 0.0063 | 0.0064 | 0.0064 | 0.0063 | 0.0064 | 0.0065 | 0.0066 | 0.0065 |
| 0.15 | -0.0010 | -0.0010 | -0.0010 | -0.0010 | -0.0010 | -0.0010 | -0.0010 | -0.0010 | -0.0010 |
| 0.2 | -0.0017 | -0.0017 | -0.0017 | -0.0017 | -0.0017 | -0.0017 | -0.0017 | -0.0017 | -0.0017 |
| 0.25 | -0.0049 | -0.0048 | -0.0048 | -0.0048 | -0.0048 | -0.0048 | -0.0048 | -0.0050 | -0.0048 |
| 0.3 | -0.0096 | -0.0096 | -0.0096 | -0.0096 | -0.0096 | -0.0096 | -0.0096 | -0.0094 | -0.0095 |
| 0.35 | -0.0134 | -0.0133 | -0.0134 | -0.0134 | -0.0134 | -0.0134 | -0.0133 | -0.0133 | -0.0133 |
| 0.4 | -0.0178 | -0.0178 | -0.0178 | -0.0178 | -0.0177 | -0.0178 | -0.0172 | -0.0178 | -0.0176 |
| 0.45 | -0.0240 | -0.0240 | -0.0240 | -0.0240 | -0.0240 | -0.0240 | -0.0244 | -0.0247 | -0.0247 |
| 0.5 | -0.0316 | -0.0316 | -0.0317 | -0.0317 | -0.0316 | -0.0317 | -0.0316 | -0.0315 | -0.0319 |
| 0.55 | -0.0362 | -0.0361 | -0.0363 | -0.0361 | -0.0359 | -0.0363 | -0.0365 | -0.0340 | -0.0357 |
| 0.6 | -0.0381 | -0.0378 | -0.0383 | -0.0380 | -0.0377 | -0.0381 | -0.0387 | -0.0373 | -0.0381 |
| 0.65 | -0.0502 | -0.0499 | -0.0504 | -0.0504 | -0.0502 | -0.0505 | -0.0507 | -0.0503 | -0.0527 |
| 0.7 | -0.0605 | -0.0605 | -0.0605 | -0.0602 | -0.0601 | -0.0603 | -0.0597 | -0.0597 | -0.0594 |
| 0.75 | -0.0611 | -0.0616 | -0.0609 | -0.0615 | -0.0620 | -0.0615 | -0.0611 | -0.0657 | -0.0636 |
| 0.8 | -0.0750 | -0.0748 | -0.0748 | -0.0744 | -0.0742 | -0.0744 | -0.0724 | -0.0753 | -0.0740 |
| 0.85 | -0.0856 | -0.0854 | -0.0855 | -0.0863 | -0.0865 | -0.0863 | -0.0828 | -0.0980 | -0.0877 |
| 0.9 | -0.0883 | -0.0884 | -0.0881 | -0.0873 | -0.0866 | -0.0876 | -0.0786 | -0.0905 | -0.0842 |
| 0.95 | -0.1159 | -0.1149 | -0.1152 | -0.1156 | -0.1165 | -0.1165 | -0.1132 | -0.1157 | -0.1172 |
| Secondary |  |  |  |  |  |  |  |  |  |
| men |  |  |  |  |  |  |  |  |  |
| 0.05 | 0.0105 | 0.0105 | 0.0104 | 0.0104 | 0.0105 | 0.0104 | 0.0100 | 0.0103 | 0.0104 |
| 0.1 | 0.0091 | 0.0091 | 0.0091 | 0.0091 | 0.0091 | 0.0090 | 0.0092 | 0.0092 | 0.0093 |
| 0.15 | 0.0051 | 0.0050 | 0.0051 | 0.0051 | 0.0051 | 0.0051 | 0.0051 | 0.0051 | 0.0050 |
| 0.2 | 0.0032 | 0.0032 | 0.0032 | 0.0032 | 0.0032 | 0.0032 | 0.0033 | 0.0031 | 0.0033 |
| 0.25 | -0.0056 | -0.0056 | -0.0057 | -0.0056 | -0.0056 | -0.0057 | -0.0055 | -0.0059 | -0.0056 |
| 0.3 | -0.0102 | -0.0101 | -0.0102 | -0.0101 | -0.0100 | -0.0102 | -0.0098 | -0.0099 | -0.0097 |
| 0.35 | -0.0133 | -0.0133 | -0.0134 | -0.0133 | -0.0133 | -0.0134 | -0.0133 | -0.0135 | -0.0127 |
| 0.4 | -0.0177 | -0.0178 | -0.0178 | -0.0179 | -0.0179 | -0.0179 | -0.0185 | -0.0188 | -0.0180 |
| 0.45 | -0.0111 | -0.0110 | -0.0111 | -0.0110 | -0.0109 | -0.0111 | -0.0106 | -0.0105 | -0.0109 |
| 0.5 | -0.0064 | -0.0063 | -0.0065 | -0.0063 | -0.0063 | -0.0064 | -0.0062 | -0.0061 | -0.0063 |
| 0.55 | -0.0107 | -0.0108 | -0.0106 | -0.0108 | -0.0108 | -0.0108 | -0.0106 | -0.0103 | -0.0107 |
| 0.6 | -0.0127 | -0.0127 | -0.0128 | -0.0128 | -0.0128 | -0.0128 | -0.0130 | -0.0140 | -0.0119 |
| 0.65 | -0.0152 | -0.0150 | -0.0154 | -0.0150 | -0.0148 | -0.0151 | -0.0154 | -0.0148 | -0.0145 |
| 0.7 | -0.0192 | -0.0192 | -0.0193 | -0.0192 | -0.0191 | -0.0193 | -0.0189 | -0.0195 | -0.0183 |
| 0.75 | -0.0157 | -0.0158 | -0.0158 | -0.0160 | -0.0161 | -0.0159 | -0.0185 | -0.0179 | -0.0146 |
| 0.8 | -0.0170 | -0.0167 | -0.0173 | -0.0167 | -0.0165 | -0.0169 | -0.0186 | -0.0168 | -0.0155 |
| 0.85 | -0.0274 | -0.0278 | -0.0275 | -0.0281 | -0.0284 | -0.0278 | -0.0290 | -0.0309 | -0.0306 |
| 0.9 | -0.0273 | -0.0275 | -0.0273 | -0.0272 | -0.0272 | -0.0271 | -0.0256 | -0.0281 | -0.0270 |
| 0.95 | -0.0412 | -0.0412 | -0.0411 | -0.0412 | -0.0413 | -0.0412 | -0.0404 | -0.0408 | -0.0395 |
| women |  |  |  |  |  |  |  |  |  |
| 0.05 | -0.0035 | -0.0035 | -0.0035 | -0.0035 | -0.0036 | -0.0035 | -0.0035 | -0.0036 | -0.0036 |
| 0.1 | -0.0083 | -0.0082 | -0.0083 | -0.0082 | -0.0082 | -0.0083 | -0.0084 | -0.0085 | -0.0085 |
| 0.15 | -0.0252 | -0.0250 | -0.0252 | -0.0252 | -0.0251 | -0.0252 | -0.0244 | -0.0252 | -0.0243 |
| 0.2 | -0.0332 | -0.0332 | -0.0333 | -0.0333 | -0.0332 | -0.0333 | -0.0336 | -0.0342 | -0.0341 |
| 0.25 | -0.0433 | -0.0430 | -0.0433 | -0.0432 | -0.0431 | -0.0432 | -0.0429 | -0.0445 | -0.0426 |
| 0.3 | -0.0522 | -0.0522 | -0.0524 | -0.0522 | -0.0521 | -0.0523 | -0.0522 | -0.0514 | -0.0517 |
| 0.35 | -0.0592 | -0.0591 | -0.0594 | -0.0594 | -0.0593 | -0.0595 | -0.0590 | -0.0587 | -0.0588 |
| 0.4 | -0.0680 | -0.0680 | -0.0680 | -0.0679 | -0.0678 | -0.0680 | -0.0658 | -0.0680 | -0.0673 |
| 0.45 | -0.0785 | -0.0783 | -0.0785 | -0.0785 | -0.0784 | -0.0785 | -0.0796 | -0.0808 | -0.0808 |
| 0.5 | -0.0882 | -0.0881 | -0.0883 | -0.0884 | -0.0882 | -0.0885 | -0.0882 | -0.0879 | -0.0890 |
| 0.55 | -0.0907 | -0.0904 | -0.0910 | -0.0905 | -0.0899 | -0.0908 | -0.0914 | -0.0852 | -0.0893 |
| 0.6 | -0.0912 | -0.0904 | -0.0917 | -0.0909 | -0.0903 | -0.0913 | -0.0925 | -0.0893 | -0.0912 |
| 0.65 | -0.1034 | -0.1026 | -0.1037 | -0.1036 | -0.1034 | -0.1038 | -0.1043 | -0.1036 | -0.1084 |
| 0.7 | -0.1125 | -0.1125 | -0.1124 | -0.1120 | -0.1118 | -0.1120 | -0.1109 | -0.1110 | -0.1104 |
| 0.75 | -0.1158 | -0.1167 | -0.1154 | -0.1165 | -0.1175 | -0.1165 | -0.1157 | -0.1244 | -0.1204 |
| 0.8 | -0.1241 | -0.1237 | -0.1237 | -0.1231 | -0.1228 | -0.1231 | -0.1198 | -0.1246 | -0.1224 |
| 0.85 | -0.1371 | -0.1368 | -0.1369 | -0.1382 | -0.1385 | -0.1383 | -0.1326 | -0.1570 | -0.1405 |
| 0.9 | -0.1309 | -0.1311 | -0.1307 | -0.1295 | -0.1285 | -0.1299 | -0.1165 | -0.1343 | -0.1249 |
| 0.95 | -0.1593 | -0.1580 | -0.1584 | -0.1590 | -0.1602 | -0.1584 | -0.1557 | -0.1591 | -0.1611 |
| Social Class II |  |  |  |  |  |  |  |  |  |
| men |  |  |  |  |  |  |  |  |  |
| 0.05 | -0.0069 | -0.0069 | -0.0068 | -0.0068 | -0.0068 | -0.0068 | -0.0065 | -0.0067 | -0.0068 |
| 0.1 | -0.0059 | -0.0060 | -0.0059 | -0.0059 | -0.0059 | -0.0059 | -0.0060 | -0.0060 | -0.0061 |
| 0.15 | 0.0002 | 0.0002 | 0.0002 | 0.0002 | 0.0002 | 0.0002 | 0.0002 | 0.0002 | 0.0002 |
| 0.2 | 0.0063 | 0.0063 | 0.0063 | 0.0063 | 0.0063 | 0.0063 | 0.0065 | 0.0062 | 0.0064 |
| 0.25 | 0.0004 | 0.0004 | 0.0004 | 0.0004 | 0.0004 | 0.0004 | 0.0004 | 0.0004 | 0.0004 |
| 0.3 | -0.0005 | -0.0005 | -0.0005 | -0.0005 | -0.0005 | -0.0005 | -0.0005 | -0.0005 | -0.0005 |
| 0.35 | 0.0006 | 0.0006 | 0.0006 | 0.0006 | 0.0006 | 0.0006 | 0.0006 | 0.0006 | 0.0006 |
| 0.4 | 0.0010 | 0.0010 | 0.0010 | 0.0010 | 0.0010 | 0.0010 | 0.0011 | 0.0011 | 0.0010 |
| 0.45 | 0.0037 | 0.0036 | 0.0037 | 0.0036 | 0.0036 | 0.0036 | 0.0035 | 0.0035 | 0.0036 |
| 0.5 | 0.0018 | 0.0018 | 0.0018 | 0.0018 | 0.0018 | 0.0018 | 0.0018 | 0.0017 | 0.0018 |
| 0.55 | 0.0041 | 0.0042 | 0.0041 | 0.0042 | 0.0042 | 0.0042 | 0.0041 | 0.0040 | 0.0042 |
| 0.6 | -0.0010 | -0.0010 | -0.0010 | -0.0010 | -0.0010 | -0.0010 | -0.0010 | -0.0011 | -0.0009 |
| 0.65 | 0.0021 | 0.0021 | 0.0022 | 0.0021 | 0.0021 | 0.0021 | 0.0022 | 0.0021 | 0.0020 |
| 0.7 | 0.0011 | 0.0011 | 0.0012 | 0.0011 | 0.0011 | 0.0011 | 0.0011 | 0.0012 | 0.0011 |
| 0.75 | -0.0045 | -0.0045 | -0.0045 | -0.0046 | -0.0046 | -0.0045 | -0.0053 | -0.0051 | -0.0042 |
| 0.8 | -0.0081 | -0.0079 | -0.0082 | -0.0080 | -0.0079 | -0.0080 | -0.0088 | -0.0080 | -0.0074 |
| 0.85 | 0.0009 | 0.0009 | 0.0009 | 0.0009 | 0.0009 | 0.0009 | 0.0009 | 0.0010 | 0.0010 |
| 0.9 | -0.0028 | -0.0028 | -0.0028 | -0.0028 | -0.0028 | -0.0028 | -0.0026 | -0.0028 | -0.0027 |
| 0.95 | 0.0078 | 0.0078 | 0.0078 | 0.0078 | 0.0078 | 0.0078 | 0.0076 | 0.0077 | 0.0075 |
| women |  |  |  |  |  |  |  |  |  |
| 0.05 | 0.0119 | 0.0120 | 0.0119 | 0.0120 | 0.0121 | 0.0120 | 0.0117 | 0.0122 | 0.0122 |
| 0.1 | 0.0190 | 0.0188 | 0.0190 | 0.0189 | 0.0187 | 0.0189 | 0.0193 | 0.0196 | 0.0194 |
| 0.15 | 0.0255 | 0.0252 | 0.0255 | 0.0254 | 0.0253 | 0.0254 | 0.0246 | 0.0254 | 0.0245 |
| 0.2 | 0.0260 | 0.0260 | 0.0260 | 0.0260 | 0.0260 | 0.0261 | 0.0263 | 0.0267 | 0.0267 |
| 0.25 | 0.0226 | 0.0224 | 0.0226 | 0.0225 | 0.0225 | 0.0226 | 0.0224 | 0.0232 | 0.0223 |
| 0.3 | 0.0278 | 0.0278 | 0.0279 | 0.0278 | 0.0277 | 0.0278 | 0.0278 | 0.0273 | 0.0275 |
| 0.35 | 0.0355 | 0.0354 | 0.0356 | 0.0356 | 0.0355 | 0.0356 | 0.0353 | 0.0352 | 0.0352 |
| 0.4 | 0.0403 | 0.0403 | 0.0403 | 0.0402 | 0.0402 | 0.0403 | 0.0390 | 0.0403 | 0.0399 |
| 0.45 | 0.0385 | 0.0384 | 0.0385 | 0.0385 | 0.0385 | 0.0386 | 0.0391 | 0.0397 | 0.0397 |
| 0.5 | 0.0392 | 0.0392 | 0.0393 | 0.0393 | 0.0392 | 0.0394 | 0.0392 | 0.0391 | 0.0396 |
| 0.55 | 0.0397 | 0.0396 | 0.0398 | 0.0396 | 0.0394 | 0.0398 | 0.0400 | 0.0373 | 0.0391 |
| 0.6 | 0.0314 | 0.0311 | 0.0316 | 0.0313 | 0.0311 | 0.0315 | 0.0319 | 0.0308 | 0.0314 |
| 0.65 | 0.0311 | 0.0308 | 0.0311 | 0.0311 | 0.0311 | 0.0312 | 0.0313 | 0.0311 | 0.0326 |
| 0.7 | 0.0352 | 0.0352 | 0.0352 | 0.0351 | 0.0350 | 0.0351 | 0.0347 | 0.0348 | 0.0346 |
| 0.75 | 0.0321 | 0.0324 | 0.0320 | 0.0323 | 0.0326 | 0.0325 | 0.0321 | 0.0345 | 0.0334 |
| 0.8 | 0.0240 | 0.0239 | 0.0239 | 0.0238 | 0.0237 | 0.0238 | 0.0231 | 0.0241 | 0.0236 |
| 0.85 | 0.0155 | 0.0155 | 0.0155 | 0.0157 | 0.0157 | 0.0157 | 0.0150 | 0.0178 | 0.0159 |
| 0.9 | 0.0143 | 0.0143 | 0.0142 | 0.0141 | 0.0140 | 0.0141 | 0.0127 | 0.0146 | 0.0136 |
| 0.95 | 0.0024 | 0.0024 | 0.0024 | 0.0024 | 0.0024 | 0.0024 | 0.0023 | 0.0024 | 0.0024 |
| Social Class III |  |  |  |  |  |  |  |  |  |
| men |  |  |  |  |  |  |  |  |  |
| 0.05 | -0.0006 | -0.0006 | -0.0006 | -0.0006 | -0.0006 | -0.0006 | -0.0006 | -0.0006 | -0.0006 |
| 0.1 | 0.0100 | 0.0101 | 0.0100 | 0.0100 | 0.0100 | 0.0100 | 0.0101 | 0.0102 | 0.0102 |
| 0.15 | 0.0152 | 0.0151 | 0.0153 | 0.0152 | 0.0152 | 0.0152 | 0.0153 | 0.0153 | 0.0150 |
| 0.2 | 0.0169 | 0.0169 | 0.0169 | 0.0169 | 0.0169 | 0.0170 | 0.0174 | 0.0166 | 0.0174 |
| 0.25 | 0.0139 | 0.0138 | 0.0141 | 0.0140 | 0.0139 | 0.0140 | 0.0137 | 0.0146 | 0.0138 |
| 0.3 | 0.0149 | 0.0148 | 0.0150 | 0.0148 | 0.0146 | 0.0149 | 0.0144 | 0.0144 | 0.0143 |
| 0.35 | 0.0142 | 0.0141 | 0.0142 | 0.0142 | 0.0141 | 0.0142 | 0.0141 | 0.0144 | 0.0135 |
| 0.4 | 0.0126 | 0.0127 | 0.0127 | 0.0128 | 0.0128 | 0.0128 | 0.0132 | 0.0134 | 0.0128 |
| 0.45 | 0.0157 | 0.0157 | 0.0158 | 0.0156 | 0.0155 | 0.0157 | 0.0151 | 0.0149 | 0.0155 |
| 0.5 | 0.0189 | 0.0186 | 0.0191 | 0.0187 | 0.0186 | 0.0188 | 0.0184 | 0.0181 | 0.0185 |
| 0.55 | 0.0188 | 0.0191 | 0.0188 | 0.0190 | 0.0191 | 0.0190 | 0.0187 | 0.0182 | 0.0189 |
| 0.6 | 0.0144 | 0.0144 | 0.0145 | 0.0145 | 0.0145 | 0.0145 | 0.0147 | 0.0159 | 0.0135 |
| 0.65 | 0.0170 | 0.0167 | 0.0172 | 0.0168 | 0.0166 | 0.0169 | 0.0172 | 0.0165 | 0.0162 |
| 0.7 | 0.0188 | 0.0188 | 0.0189 | 0.0188 | 0.0187 | 0.0189 | 0.0185 | 0.0191 | 0.0179 |
| 0.75 | 0.0151 | 0.0151 | 0.0151 | 0.0153 | 0.0154 | 0.0153 | 0.0177 | 0.0171 | 0.0140 |
| 0.8 | 0.0104 | 0.0102 | 0.0105 | 0.0102 | 0.0101 | 0.0103 | 0.0113 | 0.0103 | 0.0095 |
| 0.85 | 0.0209 | 0.0212 | 0.0210 | 0.0215 | 0.0217 | 0.0213 | 0.0222 | 0.0236 | 0.0234 |
| 0.9 | 0.0182 | 0.0184 | 0.0182 | 0.0182 | 0.0182 | 0.0181 | 0.0171 | 0.0187 | 0.0180 |
| 0.95 | 0.0298 | 0.0299 | 0.0298 | 0.0299 | 0.0300 | 0.0298 | 0.0293 | 0.0296 | 0.0287 |
| women |  |  |  |  |  |  |  |  |  |
| 0.05 | 0.0253 | 0.0256 | 0.0253 | 0.0256 | 0.0258 | 0.0255 | 0.0249 | 0.0260 | 0.0260 |
| 0.1 | 0.0374 | 0.0370 | 0.0376 | 0.0372 | 0.0370 | 0.0373 | 0.0381 | 0.0386 | 0.0383 |
| 0.15 | 0.0427 | 0.0423 | 0.0427 | 0.0426 | 0.0425 | 0.0427 | 0.0414 | 0.0427 | 0.0412 |
| 0.2 | 0.0512 | 0.0512 | 0.0513 | 0.0512 | 0.0511 | 0.0513 | 0.0518 | 0.0526 | 0.0525 |
| 0.25 | 0.0575 | 0.0571 | 0.0574 | 0.0573 | 0.0572 | 0.0574 | 0.0570 | 0.0591 | 0.0566 |
| 0.3 | 0.0603 | 0.0603 | 0.0605 | 0.0603 | 0.0602 | 0.0604 | 0.0603 | 0.0594 | 0.0598 |
| 0.35 | 0.0645 | 0.0643 | 0.0647 | 0.0647 | 0.0646 | 0.0648 | 0.0643 | 0.0640 | 0.0641 |
| 0.4 | 0.0606 | 0.0606 | 0.0605 | 0.0605 | 0.0603 | 0.0605 | 0.0585 | 0.0605 | 0.0599 |
| 0.45 | 0.0615 | 0.0614 | 0.0615 | 0.0615 | 0.0615 | 0.0616 | 0.0624 | 0.0633 | 0.0633 |
| 0.5 | 0.0629 | 0.0629 | 0.0631 | 0.0631 | 0.0630 | 0.0632 | 0.0630 | 0.0628 | 0.0636 |
| 0.55 | 0.0623 | 0.0621 | 0.0625 | 0.0622 | 0.0618 | 0.0624 | 0.0628 | 0.0585 | 0.0614 |
| 0.6 | 0.0550 | 0.0544 | 0.0552 | 0.0548 | 0.0544 | 0.0550 | 0.0557 | 0.0538 | 0.0549 |
| 0.65 | 0.0547 | 0.0542 | 0.0548 | 0.0548 | 0.0547 | 0.0549 | 0.0551 | 0.0548 | 0.0573 |
| 0.7 | 0.0622 | 0.0622 | 0.0621 | 0.0619 | 0.0618 | 0.0619 | 0.0613 | 0.0614 | 0.0611 |
| 0.75 | 0.0593 | 0.0598 | 0.0591 | 0.0597 | 0.0602 | 0.0595 | 0.0593 | 0.0638 | 0.0617 |
| 0.8 | 0.0534 | 0.0533 | 0.0533 | 0.0530 | 0.0529 | 0.0530 | 0.0516 | 0.0536 | 0.0527 |
| 0.85 | 0.0466 | 0.0466 | 0.0466 | 0.0470 | 0.0471 | 0.0471 | 0.0451 | 0.0534 | 0.0478 |
| 0.9 | 0.0409 | 0.0409 | 0.0408 | 0.0404 | 0.0401 | 0.0405 | 0.0364 | 0.0419 | 0.0390 |
| 0.95 | 0.0325 | 0.0322 | 0.0323 | 0.0324 | 0.0327 | 0.0325 | 0.0317 | 0.0324 | 0.0329 |
| Social Class IV |  |  |  |  |  |  |  |  |  |
| men |  |  |  |  |  |  |  |  |  |
| 0.05 | -0.0199 | -0.0200 | -0.0197 | -0.0198 | -0.0199 | -0.0197 | -0.0190 | -0.0196 | -0.0198 |
| 0.1 | 0.0025 | 0.0025 | 0.0025 | 0.0025 | 0.0025 | 0.0025 | 0.0025 | 0.0026 | 0.0026 |
| 0.15 | 0.0084 | 0.0084 | 0.0084 | 0.0084 | 0.0084 | 0.0084 | 0.0085 | 0.0084 | 0.0083 |
| 0.2 | 0.0136 | 0.0136 | 0.0136 | 0.0136 | 0.0136 | 0.0136 | 0.0140 | 0.0133 | 0.0140 |
| 0.25 | 0.0114 | 0.0112 | 0.0115 | 0.0114 | 0.0114 | 0.0115 | 0.0112 | 0.0119 | 0.0113 |
| 0.3 | 0.0142 | 0.0141 | 0.0143 | 0.0141 | 0.0139 | 0.0142 | 0.0137 | 0.0138 | 0.0136 |
| 0.35 | 0.0161 | 0.0160 | 0.0162 | 0.0161 | 0.0160 | 0.0162 | 0.0160 | 0.0164 | 0.0153 |
| 0.4 | 0.0191 | 0.0192 | 0.0192 | 0.0193 | 0.0194 | 0.0194 | 0.0200 | 0.0203 | 0.0194 |
| 0.45 | 0.0203 | 0.0202 | 0.0203 | 0.0202 | 0.0201 | 0.0203 | 0.0195 | 0.0193 | 0.0201 |
| 0.5 | 0.0225 | 0.0222 | 0.0227 | 0.0223 | 0.0222 | 0.0225 | 0.0220 | 0.0216 | 0.0220 |
| 0.55 | 0.0212 | 0.0215 | 0.0211 | 0.0214 | 0.0214 | 0.0213 | 0.0210 | 0.0205 | 0.0212 |
| 0.6 | 0.0168 | 0.0168 | 0.0169 | 0.0169 | 0.0169 | 0.0169 | 0.0171 | 0.0185 | 0.0158 |
| 0.65 | 0.0212 | 0.0208 | 0.0214 | 0.0209 | 0.0206 | 0.0211 | 0.0215 | 0.0206 | 0.0201 |
| 0.7 | 0.0236 | 0.0237 | 0.0237 | 0.0236 | 0.0235 | 0.0237 | 0.0233 | 0.0240 | 0.0224 |
| 0.75 | 0.0201 | 0.0202 | 0.0202 | 0.0205 | 0.0206 | 0.0205 | 0.0237 | 0.0229 | 0.0188 |
| 0.8 | 0.0144 | 0.0141 | 0.0146 | 0.0142 | 0.0140 | 0.0143 | 0.0157 | 0.0143 | 0.0132 |
| 0.85 | 0.0292 | 0.0296 | 0.0293 | 0.0299 | 0.0303 | 0.0297 | 0.0309 | 0.0329 | 0.0326 |
| 0.9 | 0.0219 | 0.0221 | 0.0219 | 0.0218 | 0.0218 | 0.0217 | 0.0205 | 0.0225 | 0.0216 |
| 0.95 | 0.0214 | 0.0214 | 0.0214 | 0.0215 | 0.0215 | 0.0214 | 0.0210 | 0.0212 | 0.0206 |
| women |  |  |  |  |  |  |  |  |  |
| 0.05 | 0.0386 | 0.0390 | 0.0385 | 0.0390 | 0.0393 | 0.0389 | 0.0380 | 0.0396 | 0.0396 |
| 0.1 | 0.0485 | 0.0480 | 0.0487 | 0.0482 | 0.0479 | 0.0484 | 0.0493 | 0.0500 | 0.0496 |
| 0.15 | 0.0593 | 0.0587 | 0.0593 | 0.0591 | 0.0590 | 0.0592 | 0.0574 | 0.0592 | 0.0572 |
| 0.2 | 0.0746 | 0.0746 | 0.0747 | 0.0747 | 0.0745 | 0.0748 | 0.0755 | 0.0767 | 0.0766 |
| 0.25 | 0.0820 | 0.0813 | 0.0819 | 0.0817 | 0.0815 | 0.0814 | 0.0812 | 0.0842 | 0.0807 |
| 0.3 | 0.0910 | 0.0909 | 0.0912 | 0.0910 | 0.0907 | 0.0911 | 0.0909 | 0.0895 | 0.0901 |
| 0.35 | 0.0987 | 0.0985 | 0.0990 | 0.0990 | 0.0989 | 0.0991 | 0.0984 | 0.0979 | 0.0981 |
| 0.4 | 0.0997 | 0.0998 | 0.0997 | 0.0996 | 0.0994 | 0.0997 | 0.0964 | 0.0997 | 0.0986 |
| 0.45 | 0.1012 | 0.1010 | 0.1013 | 0.1013 | 0.1012 | 0.1013 | 0.1027 | 0.1042 | 0.1042 |
| 0.5 | 0.1049 | 0.1049 | 0.1051 | 0.1052 | 0.1050 | 0.1054 | 0.1050 | 0.1047 | 0.1059 |
| 0.55 | 0.1034 | 0.1031 | 0.1038 | 0.1032 | 0.1025 | 0.1036 | 0.1043 | 0.0971 | 0.1019 |
| 0.6 | 0.0965 | 0.0956 | 0.0969 | 0.0962 | 0.0955 | 0.0965 | 0.0978 | 0.0945 | 0.0964 |
| 0.65 | 0.0944 | 0.0937 | 0.0947 | 0.0946 | 0.0944 | 0.0948 | 0.0952 | 0.0946 | 0.0990 |
| 0.7 | 0.0991 | 0.0991 | 0.0990 | 0.0987 | 0.0985 | 0.0987 | 0.0977 | 0.0978 | 0.0973 |
| 0.75 | 0.0960 | 0.0968 | 0.0957 | 0.0966 | 0.0974 | 0.0965 | 0.0959 | 0.1032 | 0.0999 |
| 0.8 | 0.0902 | 0.0899 | 0.0899 | 0.0895 | 0.0893 | 0.0895 | 0.0871 | 0.0906 | 0.0890 |
| 0.85 | 0.0851 | 0.0850 | 0.0850 | 0.0858 | 0.0860 | 0.0859 | 0.0823 | 0.0975 | 0.0872 |
| 0.9 | 0.0754 | 0.0755 | 0.0753 | 0.0746 | 0.0740 | 0.0758 | 0.0671 | 0.0773 | 0.0719 |
| 0.95 | 0.0758 | 0.0752 | 0.0754 | 0.0757 | 0.0762 | 0.0754 | 0.0741 | 0.0757 | 0.0767 |
| Social Class V |  |  |  |  |  |  |  |  |  |
| men |  |  |  |  |  |  |  |  |  |
| 0.05 | -0.0186 | -0.0187 | -0.0184 | -0.0185 | -0.0186 | -0.0184 | -0.0178 | -0.0183 | -0.0185 |
| 0.1 | -0.0065 | -0.0065 | -0.0065 | -0.0065 | -0.0065 | -0.0064 | -0.0066 | -0.0066 | -0.0066 |
| 0.15 | 0.0080 | 0.0080 | 0.0080 | 0.0080 | 0.0080 | 0.0080 | 0.0081 | 0.0080 | 0.0079 |
| 0.2 | 0.0131 | 0.0131 | 0.0131 | 0.0131 | 0.0131 | 0.0131 | 0.0135 | 0.0128 | 0.0134 |
| 0.25 | 0.0192 | 0.0190 | 0.0194 | 0.0193 | 0.0192 | 0.0194 | 0.0189 | 0.0201 | 0.0191 |
| 0.3 | 0.0198 | 0.0197 | 0.0200 | 0.0197 | 0.0195 | 0.0199 | 0.0192 | 0.0192 | 0.0190 |
| 0.35 | 0.0226 | 0.0224 | 0.0227 | 0.0226 | 0.0225 | 0.0226 | 0.0225 | 0.0229 | 0.0214 |
| 0.4 | 0.0227 | 0.0228 | 0.0228 | 0.0229 | 0.0230 | 0.0230 | 0.0237 | 0.0240 | 0.0230 |
| 0.45 | 0.0253 | 0.0252 | 0.0253 | 0.0251 | 0.0250 | 0.0253 | 0.0242 | 0.0240 | 0.0250 |
| 0.5 | 0.0299 | 0.0295 | 0.0302 | 0.0297 | 0.0295 | 0.0294 | 0.0292 | 0.0287 | 0.0293 |
| 0.55 | 0.0313 | 0.0318 | 0.0312 | 0.0316 | 0.0317 | 0.0316 | 0.0312 | 0.0303 | 0.0315 |
| 0.6 | 0.0263 | 0.0263 | 0.0265 | 0.0265 | 0.0265 | 0.0265 | 0.0269 | 0.0290 | 0.0247 |
| 0.65 | 0.0241 | 0.0237 | 0.0243 | 0.0237 | 0.0234 | 0.0239 | 0.0244 | 0.0234 | 0.0229 |
| 0.7 | 0.0245 | 0.0246 | 0.0246 | 0.0245 | 0.0244 | 0.0246 | 0.0242 | 0.0249 | 0.0233 |
| 0.75 | 0.0221 | 0.0221 | 0.0221 | 0.0224 | 0.0225 | 0.0225 | 0.0259 | 0.0251 | 0.0206 |
| 0.8 | 0.0190 | 0.0186 | 0.0192 | 0.0187 | 0.0184 | 0.0188 | 0.0207 | 0.0188 | 0.0173 |
| 0.85 | 0.0285 | 0.0289 | 0.0286 | 0.0292 | 0.0295 | 0.0289 | 0.0301 | 0.0321 | 0.0318 |
| 0.9 | 0.0280 | 0.0282 | 0.0281 | 0.0279 | 0.0280 | 0.0278 | 0.0263 | 0.0288 | 0.0277 |
| 0.95 | 0.0423 | 0.0424 | 0.0423 | 0.0424 | 0.0425 | 0.0423 | 0.0415 | 0.0420 | 0.0407 |
| women |  |  |  |  |  |  |  |  |  |
| 0.05 | 0.0330 | 0.0334 | 0.0330 | 0.0334 | 0.0336 | 0.0333 | 0.0325 | 0.0339 | 0.0339 |
| 0.1 | 0.0449 | 0.0445 | 0.0451 | 0.0447 | 0.0444 | 0.0448 | 0.0457 | 0.0464 | 0.0459 |
| 0.15 | 0.0582 | 0.0576 | 0.0582 | 0.0580 | 0.0579 | 0.0581 | 0.0563 | 0.0581 | 0.0561 |
| 0.2 | 0.0713 | 0.0712 | 0.0713 | 0.0713 | 0.0711 | 0.0714 | 0.0721 | 0.0733 | 0.0731 |
| 0.25 | 0.0806 | 0.0800 | 0.0806 | 0.0804 | 0.0802 | 0.0805 | 0.0799 | 0.0829 | 0.0794 |
| 0.3 | 0.0879 | 0.0879 | 0.0882 | 0.0879 | 0.0877 | 0.0881 | 0.0879 | 0.0865 | 0.0871 |
| 0.35 | 0.0940 | 0.0937 | 0.0942 | 0.0942 | 0.0941 | 0.0943 | 0.0937 | 0.0932 | 0.0934 |
| 0.4 | 0.0949 | 0.0950 | 0.0949 | 0.0948 | 0.0946 | 0.0949 | 0.0918 | 0.0949 | 0.0939 |
| 0.45 | 0.0961 | 0.0959 | 0.0961 | 0.0961 | 0.0961 | 0.0962 | 0.0975 | 0.0989 | 0.0990 |
| 0.5 | 0.1005 | 0.1004 | 0.1006 | 0.1007 | 0.1005 | 0.1004 | 0.1005 | 0.1002 | 0.1014 |
| 0.55 | 0.0984 | 0.0981 | 0.0987 | 0.0982 | 0.0976 | 0.0986 | 0.0992 | 0.0924 | 0.0969 |
| 0.6 | 0.0870 | 0.0861 | 0.0873 | 0.0867 | 0.0861 | 0.0870 | 0.0882 | 0.0851 | 0.0869 |
| 0.65 | 0.0855 | 0.0849 | 0.0858 | 0.0857 | 0.0855 | 0.0859 | 0.0863 | 0.0857 | 0.0897 |
| 0.7 | 0.0909 | 0.0909 | 0.0908 | 0.0905 | 0.0903 | 0.0905 | 0.0896 | 0.0897 | 0.0892 |
| 0.75 | 0.0853 | 0.0860 | 0.0850 | 0.0858 | 0.0865 | 0.0865 | 0.0852 | 0.0917 | 0.0887 |
| 0.8 | 0.0846 | 0.0843 | 0.0844 | 0.0839 | 0.0837 | 0.0839 | 0.0817 | 0.0849 | 0.0835 |
| 0.85 | 0.0824 | 0.0822 | 0.0823 | 0.0831 | 0.0832 | 0.0831 | 0.0797 | 0.0943 | 0.0844 |
| 0.9 | 0.0665 | 0.0666 | 0.0663 | 0.0657 | 0.0652 | 0.0659 | 0.0592 | 0.0682 | 0.0634 |
| 0.95 | 0.0619 | 0.0614 | 0.0615 | 0.0617 | 0.0622 | 0.0615 | 0.0604 | 0.0618 | 0.0626 |
| Social Class VI |  |  |  |  |  |  |  |  |  |
| men |  |  |  |  |  |  |  |  |  |
| 0.05 | -0.0333 | -0.0334 | -0.0329 | -0.0330 | -0.0332 | -0.0328 | -0.0318 | -0.0328 | -0.0331 |
| 0.1 | -0.0124 | -0.0125 | -0.0124 | -0.0124 | -0.0124 | -0.0123 | -0.0126 | -0.0126 | -0.0127 |
| 0.15 | 0.0023 | 0.0023 | 0.0023 | 0.0023 | 0.0023 | 0.0023 | 0.0023 | 0.0023 | 0.0022 |
| 0.2 | 0.0063 | 0.0063 | 0.0063 | 0.0063 | 0.0063 | 0.0063 | 0.0064 | 0.0061 | 0.0064 |
| 0.25 | 0.0099 | 0.0098 | 0.0100 | 0.0099 | 0.0099 | 0.0100 | 0.0097 | 0.0103 | 0.0098 |
| 0.3 | 0.0129 | 0.0129 | 0.0130 | 0.0128 | 0.0127 | 0.0129 | 0.0125 | 0.0125 | 0.0124 |
| 0.35 | 0.0169 | 0.0168 | 0.0169 | 0.0169 | 0.0168 | 0.0169 | 0.0168 | 0.0171 | 0.0160 |
| 0.4 | 0.0192 | 0.0193 | 0.0193 | 0.0194 | 0.0195 | 0.0195 | 0.0201 | 0.0204 | 0.0195 |
| 0.45 | 0.0183 | 0.0182 | 0.0184 | 0.0182 | 0.0181 | 0.0183 | 0.0176 | 0.0174 | 0.0181 |
| 0.5 | 0.0170 | 0.0168 | 0.0171 | 0.0168 | 0.0167 | 0.0169 | 0.0166 | 0.0163 | 0.0166 |
| 0.55 | 0.0213 | 0.0216 | 0.0212 | 0.0215 | 0.0216 | 0.0215 | 0.0212 | 0.0206 | 0.0214 |
| 0.6 | 0.0180 | 0.0180 | 0.0181 | 0.0182 | 0.0182 | 0.0182 | 0.0184 | 0.0199 | 0.0169 |
| 0.65 | 0.0192 | 0.0189 | 0.0194 | 0.0190 | 0.0187 | 0.0191 | 0.0195 | 0.0187 | 0.0182 |
| 0.7 | 0.0225 | 0.0225 | 0.0226 | 0.0224 | 0.0223 | 0.0225 | 0.0221 | 0.0228 | 0.0213 |
| 0.75 | 0.0171 | 0.0171 | 0.0171 | 0.0174 | 0.0175 | 0.0173 | 0.0201 | 0.0194 | 0.0159 |
| 0.8 | 0.0141 | 0.0138 | 0.0142 | 0.0138 | 0.0136 | 0.0139 | 0.0154 | 0.0139 | 0.0128 |
| 0.85 | 0.0291 | 0.0295 | 0.0292 | 0.0298 | 0.0302 | 0.0295 | 0.0308 | 0.0328 | 0.0325 |
| 0.9 | 0.0350 | 0.0352 | 0.0350 | 0.0348 | 0.0349 | 0.0347 | 0.0327 | 0.0359 | 0.0346 |
| 0.95 | 0.0460 | 0.0461 | 0.0460 | 0.0461 | 0.0462 | 0.0460 | 0.0452 | 0.0457 | 0.0442 |
| women |  |  |  |  |  |  |  |  |  |
| 0.05 | 0.0355 | 0.0359 | 0.0354 | 0.0359 | 0.0361 | 0.0357 | 0.0349 | 0.0364 | 0.0364 |
| 0.1 | 0.0546 | 0.0540 | 0.0548 | 0.0543 | 0.0539 | 0.0545 | 0.0555 | 0.0563 | 0.0558 |
| 0.15 | 0.0694 | 0.0687 | 0.0694 | 0.0692 | 0.0690 | 0.0693 | 0.0672 | 0.0693 | 0.0669 |
| 0.2 | 0.0805 | 0.0805 | 0.0806 | 0.0806 | 0.0804 | 0.0807 | 0.0815 | 0.0828 | 0.0826 |
| 0.25 | 0.0875 | 0.0868 | 0.0874 | 0.0872 | 0.0870 | 0.0873 | 0.0867 | 0.0899 | 0.0861 |
| 0.3 | 0.0974 | 0.0973 | 0.0976 | 0.0974 | 0.0971 | 0.0975 | 0.0973 | 0.0958 | 0.0964 |
| 0.35 | 0.1089 | 0.1086 | 0.1092 | 0.1092 | 0.1091 | 0.1093 | 0.1085 | 0.1080 | 0.1082 |
| 0.4 | 0.1043 | 0.1043 | 0.1042 | 0.1041 | 0.1039 | 0.1042 | 0.1008 | 0.1042 | 0.1031 |
| 0.45 | 0.1072 | 0.1070 | 0.1072 | 0.1072 | 0.1072 | 0.1073 | 0.1088 | 0.1104 | 0.1104 |
| 0.5 | 0.1129 | 0.1129 | 0.1131 | 0.1132 | 0.1130 | 0.1134 | 0.1130 | 0.1127 | 0.1140 |
| 0.55 | 0.1062 | 0.1059 | 0.1066 | 0.1060 | 0.1053 | 0.1064 | 0.1071 | 0.0997 | 0.1046 |
| 0.6 | 0.1044 | 0.1034 | 0.1049 | 0.1040 | 0.1033 | 0.1044 | 0.1059 | 0.1022 | 0.1043 |
| 0.65 | 0.1071 | 0.1063 | 0.1074 | 0.1074 | 0.1071 | 0.1076 | 0.1080 | 0.1073 | 0.1123 |
| 0.7 | 0.1167 | 0.1167 | 0.1166 | 0.1162 | 0.1160 | 0.1162 | 0.1150 | 0.1152 | 0.1145 |
| 0.75 | 0.1129 | 0.1138 | 0.1126 | 0.1137 | 0.1146 | 0.1145 | 0.1128 | 0.1214 | 0.1175 |
| 0.8 | 0.1152 | 0.1148 | 0.1149 | 0.1142 | 0.1140 | 0.1143 | 0.1112 | 0.1157 | 0.1136 |
| 0.85 | 0.1100 | 0.1097 | 0.1098 | 0.1109 | 0.1111 | 0.1109 | 0.1064 | 0.1259 | 0.1127 |
| 0.9 | 0.1081 | 0.1082 | 0.1079 | 0.1069 | 0.1060 | 0.1072 | 0.0962 | 0.1108 | 0.1031 |
| 0.95 | 0.1029 | 0.1021 | 0.1023 | 0.1027 | 0.1035 | 0.1025 | 0.1006 | 0.1027 | 0.1041 |
| Universitary |  |  |  |  |  |  |  |  |  |
| men |  |  |  |  |  |  |  |  |  |
| 0.05 | 0.0329 | 0.0330 | 0.0325 | 0.0326 | 0.0328 | 0.0325 | 0.0314 | 0.0324 | 0.0327 |
| 0.1 | 0.0082 | 0.0082 | 0.0082 | 0.0082 | 0.0082 | 0.0082 | 0.0083 | 0.0084 | 0.0084 |
| 0.15 | -0.0040 | -0.0040 | -0.0040 | -0.0040 | -0.0040 | -0.0040 | -0.0041 | -0.0040 | -0.0040 |
| 0.2 | -0.0073 | -0.0073 | -0.0073 | -0.0073 | -0.0073 | -0.0074 | -0.0075 | -0.0072 | -0.0075 |
| 0.25 | -0.0206 | -0.0204 | -0.0209 | -0.0207 | -0.0206 | -0.0208 | -0.0203 | -0.0216 | -0.0205 |
| 0.3 | -0.0235 | -0.0234 | -0.0236 | -0.0233 | -0.0231 | -0.0235 | -0.0227 | -0.0228 | -0.0225 |
| 0.35 | -0.0340 | -0.0339 | -0.0342 | -0.0340 | -0.0339 | -0.0342 | -0.0339 | -0.0346 | -0.0323 |
| 0.4 | -0.0382 | -0.0383 | -0.0383 | -0.0385 | -0.0386 | -0.0386 | -0.0398 | -0.0404 | -0.0386 |
| 0.45 | -0.0343 | -0.0342 | -0.0344 | -0.0341 | -0.0339 | -0.0343 | -0.0329 | -0.0326 | -0.0339 |
| 0.5 | -0.0288 | -0.0284 | -0.0291 | -0.0286 | -0.0284 | -0.0284 | -0.0281 | -0.0276 | -0.0282 |
| 0.55 | -0.0320 | -0.0324 | -0.0319 | -0.0322 | -0.0323 | -0.0322 | -0.0318 | -0.0309 | -0.0321 |
| 0.6 | -0.0348 | -0.0347 | -0.0350 | -0.0350 | -0.0351 | -0.0351 | -0.0355 | -0.0384 | -0.0327 |
| 0.65 | -0.0384 | -0.0377 | -0.0387 | -0.0378 | -0.0373 | -0.0381 | -0.0388 | -0.0373 | -0.0364 |
| 0.7 | -0.0432 | -0.0432 | -0.0434 | -0.0431 | -0.0429 | -0.0433 | -0.0425 | -0.0438 | -0.0410 |
| 0.75 | -0.0394 | -0.0396 | -0.0396 | -0.0401 | -0.0403 | -0.0400 | -0.0464 | -0.0449 | -0.0368 |
| 0.8 | -0.0420 | -0.0411 | -0.0425 | -0.0412 | -0.0407 | -0.0415 | -0.0458 | -0.0415 | -0.0383 |
| 0.85 | -0.0558 | -0.0566 | -0.0560 | -0.0571 | -0.0579 | -0.0567 | -0.0590 | -0.0629 | -0.0624 |
| 0.9 | -0.0539 | -0.0543 | -0.0539 | -0.0537 | -0.0538 | -0.0535 | -0.0505 | -0.0554 | -0.0533 |
| 0.95 | -0.0710 | -0.0710 | -0.0709 | -0.0711 | -0.0712 | -0.0710 | -0.0696 | -0.0704 | -0.0682 |
| women |  |  |  |  |  |  |  |  |  |
| 0.05 | -0.0226 | -0.0229 | -0.0226 | -0.0229 | -0.0231 | -0.0228 | -0.0223 | -0.0232 | -0.0232 |
| 0.1 | -0.0385 | -0.0381 | -0.0386 | -0.0383 | -0.0380 | -0.0384 | -0.0391 | -0.0397 | -0.0393 |
| 0.15 | -0.0649 | -0.0643 | -0.0649 | -0.0647 | -0.0646 | -0.0649 | -0.0628 | -0.0648 | -0.0626 |
| 0.2 | -0.0821 | -0.0821 | -0.0822 | -0.0821 | -0.0819 | -0.0823 | -0.0831 | -0.0844 | -0.0842 |
| 0.25 | -0.0990 | -0.0983 | -0.0989 | -0.0987 | -0.0985 | -0.0984 | -0.0981 | -0.1018 | -0.0975 |
| 0.3 | -0.1099 | -0.1098 | -0.1102 | -0.1099 | -0.1095 | -0.1100 | -0.1098 | -0.1081 | -0.1088 |
| 0.35 | -0.1188 | -0.1185 | -0.1191 | -0.1192 | -0.1190 | -0.1193 | -0.1184 | -0.1178 | -0.1181 |
| 0.4 | -0.1278 | -0.1278 | -0.1277 | -0.1276 | -0.1274 | -0.1278 | -0.1236 | -0.1278 | -0.1264 |
| 0.45 | -0.1377 | -0.1374 | -0.1378 | -0.1378 | -0.1377 | -0.1379 | -0.1398 | -0.1418 | -0.1418 |
| 0.5 | -0.1452 | -0.1451 | -0.1454 | -0.1455 | -0.1452 | -0.1455 | -0.1452 | -0.1448 | -0.1466 |
| 0.55 | -0.1468 | -0.1464 | -0.1473 | -0.1465 | -0.1456 | -0.1470 | -0.1480 | -0.1379 | -0.1446 |
| 0.6 | -0.1460 | -0.1447 | -0.1467 | -0.1455 | -0.1445 | -0.1461 | -0.1481 | -0.1430 | -0.1459 |
| 0.65 | -0.1545 | -0.1533 | -0.1550 | -0.1549 | -0.1545 | -0.1552 | -0.1559 | -0.1548 | -0.1620 |
| 0.7 | -0.1655 | -0.1655 | -0.1653 | -0.1648 | -0.1645 | -0.1648 | -0.1632 | -0.1634 | -0.1625 |
| 0.75 | -0.1643 | -0.1656 | -0.1638 | -0.1653 | -0.1666 | -0.1668 | -0.1641 | -0.1766 | -0.1709 |
| 0.8 | -0.1700 | -0.1695 | -0.1695 | -0.1686 | -0.1683 | -0.1687 | -0.1642 | -0.1707 | -0.1677 |
| 0.85 | -0.1781 | -0.1778 | -0.1779 | -0.1796 | -0.1799 | -0.1797 | -0.1723 | -0.2039 | -0.1825 |
| 0.9 | -0.1706 | -0.1708 | -0.1702 | -0.1687 | -0.1674 | -0.1692 | -0.1518 | -0.1749 | -0.1627 |
| 0.95 | -0.1925 | -0.1909 | -0.1914 | -0.1921 | -0.1936 | -0.1942 | -0.1881 | -0.1922 | -0.1946 |
| 2014 |  |  |  |  |  |  |  |  |  |
| Income 25 |  |  |  |  |  |  |  |  |  |
| men |  |  |  |  |  |  |  |  |  |
| 0.05 | 0.0001 | 0.0001 | 0.0001 | 0.0001 | 0.0001 | 0.0001 | 0.0001 | 0.0001 | 0.0001 |
| 0.1 | -0.0154 | -0.0155 | -0.0152 | -0.0153 | -0.0155 | -0.0152 | -0.0160 | -0.0156 | -0.0156 |
| 0.15 | -0.0133 | -0.0133 | -0.0133 | -0.0133 | -0.0134 | -0.0133 | -0.0131 | -0.0136 | -0.0135 |
| 0.2 | -0.0196 | -0.0196 | -0.0196 | -0.0196 | -0.0196 | -0.0197 | -0.0199 | -0.0193 | -0.0209 |
| 0.25 | -0.0167 | -0.0164 | -0.0169 | -0.0167 | -0.0165 | -0.0168 | -0.0165 | -0.0171 | -0.0160 |
| 0.3 | -0.0160 | -0.0159 | -0.0162 | -0.0159 | -0.0157 | -0.0161 | -0.0149 | -0.0159 | -0.0153 |
| 0.35 | -0.0169 | -0.0168 | -0.0169 | -0.0168 | -0.0167 | -0.0169 | -0.0175 | -0.0172 | -0.0164 |
| 0.4 | -0.0175 | -0.0176 | -0.0175 | -0.0177 | -0.0178 | -0.0177 | -0.0175 | -0.0181 | -0.0175 |
| 0.45 | -0.0114 | -0.0113 | -0.0115 | -0.0114 | -0.0113 | -0.0115 | -0.0109 | -0.0113 | -0.0114 |
| 0.5 | -0.0162 | -0.0159 | -0.0164 | -0.0159 | -0.0157 | -0.0161 | -0.0154 | -0.0152 | -0.0157 |
| 0.55 | -0.0167 | -0.0168 | -0.0167 | -0.0167 | -0.0167 | -0.0168 | -0.0166 | -0.0164 | -0.0166 |
| 0.6 | -0.0226 | -0.0229 | -0.0226 | -0.0229 | -0.0231 | -0.0228 | -0.0232 | -0.0261 | -0.0214 |
| 0.65 | -0.0186 | -0.0185 | -0.0187 | -0.0184 | -0.0183 | -0.0185 | -0.0199 | -0.0192 | -0.0172 |
| 0.7 | -0.0197 | -0.0196 | -0.0198 | -0.0197 | -0.0197 | -0.0198 | -0.0190 | -0.0192 | -0.0202 |
| 0.75 | -0.0213 | -0.0214 | -0.0214 | -0.0215 | -0.0215 | -0.0215 | -0.0202 | -0.0218 | -0.0211 |
| 0.8 | -0.0243 | -0.0246 | -0.0242 | -0.0242 | -0.0241 | -0.0242 | -0.0233 | -0.0224 | -0.0255 |
| 0.85 | -0.0283 | -0.0285 | -0.0286 | -0.0288 | -0.0290 | -0.0287 | -0.0309 | -0.0309 | -0.0268 |
| 0.9 | -0.0274 | -0.0274 | -0.0274 | -0.0276 | -0.0277 | -0.0276 | -0.0274 | -0.0267 | -0.0295 |
| 0.95 | -0.0487 | -0.0493 | -0.0491 | -0.0499 | -0.0506 | -0.0506 | -0.0532 | -0.0576 | -0.0511 |
| women |  |  |  |  |  |  |  |  |  |
| 0.05 | 0.0051 | 0.0051 | 0.0052 | 0.0051 | 0.0051 | 0.0051 | 0.0051 | 0.0047 | 0.0050 |
| 0.1 | 0.0084 | 0.0083 | 0.0084 | 0.0084 | 0.0084 | 0.0084 | 0.0081 | 0.0087 | 0.0084 |
| 0.15 | 0.0105 | 0.0106 | 0.0105 | 0.0105 | 0.0105 | 0.0105 | 0.0110 | 0.0102 | 0.0104 |
| 0.2 | 0.0075 | 0.0075 | 0.0075 | 0.0075 | 0.0075 | 0.0075 | 0.0077 | 0.0075 | 0.0076 |
| 0.25 | 0.0054 | 0.0054 | 0.0054 | 0.0054 | 0.0054 | 0.0054 | 0.0054 | 0.0053 | 0.0052 |
| 0.3 | 0.0091 | 0.0091 | 0.0092 | 0.0091 | 0.0091 | 0.0092 | 0.0087 | 0.0087 | 0.0089 |
| 0.35 | 0.0086 | 0.0085 | 0.0086 | 0.0086 | 0.0085 | 0.0086 | 0.0085 | 0.0083 | 0.0087 |
| 0.4 | 0.0030 | 0.0030 | 0.0030 | 0.0030 | 0.0030 | 0.0030 | 0.0029 | 0.0030 | 0.0030 |
| 0.45 | 0.0010 | 0.0010 | 0.0010 | 0.0010 | 0.0010 | 0.0010 | 0.0009 | 0.0010 | 0.0010 |
| 0.5 | -0.0031 | -0.0031 | -0.0031 | -0.0031 | -0.0031 | -0.0031 | -0.0030 | -0.0032 | -0.0031 |
| 0.55 | -0.0060 | -0.0060 | -0.0060 | -0.0060 | -0.0059 | -0.0060 | -0.0058 | -0.0058 | -0.0060 |
| 0.6 | -0.0125 | -0.0125 | -0.0125 | -0.0125 | -0.0125 | -0.0125 | -0.0122 | -0.0123 | -0.0123 |
| 0.65 | -0.0137 | -0.0137 | -0.0137 | -0.0138 | -0.0138 | -0.0137 | -0.0140 | -0.0135 | -0.0146 |
| 0.7 | -0.0284 | -0.0281 | -0.0284 | -0.0281 | -0.0279 | -0.0281 | -0.0263 | -0.0260 | -0.0272 |
| 0.75 | -0.0344 | -0.0343 | -0.0345 | -0.0345 | -0.0344 | -0.0345 | -0.0351 | -0.0336 | -0.0325 |
| 0.8 | -0.0578 | -0.0578 | -0.0571 | -0.0574 | -0.0575 | -0.0573 | -0.0564 | -0.0588 | -0.0579 |
| 0.85 | -0.0463 | -0.0461 | -0.0464 | -0.0465 | -0.0465 | -0.0464 | -0.0447 | -0.0404 | -0.0469 |
| 0.9 | -0.0363 | -0.0361 | -0.0365 | -0.0367 | -0.0368 | -0.0367 | -0.0339 | -0.0358 | -0.0358 |
| 0.95 | -0.0450 | -0.0454 | -0.0451 | -0.0457 | -0.0464 | -0.0455 | -0.0527 | -0.0494 | -0.0469 |
| Income 50 |  |  |  |  |  |  |  |  |  |
| men |  |  |  |  |  |  |  |  |  |
| 0.05 | 0.0137 | 0.0136 | 0.0137 | 0.0136 | 0.0135 | 0.0136 | 0.0138 | 0.0132 | 0.0129 |
| 0.1 | 0.0130 | 0.0131 | 0.0128 | 0.0130 | 0.0131 | 0.0128 | 0.0135 | 0.0132 | 0.0132 |
| 0.15 | 0.0097 | 0.0097 | 0.0097 | 0.0097 | 0.0098 | 0.0097 | 0.0096 | 0.0099 | 0.0098 |
| 0.2 | 0.0060 | 0.0061 | 0.0060 | 0.0060 | 0.0060 | 0.0061 | 0.0061 | 0.0059 | 0.0064 |
| 0.25 | 0.0042 | 0.0042 | 0.0043 | 0.0042 | 0.0042 | 0.0043 | 0.0042 | 0.0044 | 0.0041 |
| 0.3 | 0.0050 | 0.0049 | 0.0050 | 0.0049 | 0.0049 | 0.0050 | 0.0046 | 0.0049 | 0.0048 |
| 0.35 | 0.0029 | 0.0029 | 0.0029 | 0.0029 | 0.0029 | 0.0029 | 0.0030 | 0.0030 | 0.0029 |
| 0.4 | -0.0031 | -0.0032 | -0.0031 | -0.0032 | -0.0032 | -0.0032 | -0.0031 | -0.0032 | -0.0031 |
| 0.45 | -0.0058 | -0.0058 | -0.0059 | -0.0058 | -0.0058 | -0.0059 | -0.0056 | -0.0058 | -0.0059 |
| 0.5 | -0.0045 | -0.0044 | -0.0045 | -0.0044 | -0.0043 | -0.0044 | -0.0042 | -0.0042 | -0.0043 |
| 0.55 | -0.0007 | -0.0007 | -0.0007 | -0.0007 | -0.0007 | -0.0007 | -0.0007 | -0.0007 | -0.0007 |
| 0.6 | -0.0083 | -0.0084 | -0.0083 | -0.0084 | -0.0085 | -0.0084 | -0.0085 | -0.0096 | -0.0079 |
| 0.65 | -0.0050 | -0.0050 | -0.0050 | -0.0050 | -0.0050 | -0.0050 | -0.0054 | -0.0052 | -0.0046 |
| 0.7 | -0.0100 | -0.0099 | -0.0101 | -0.0100 | -0.0100 | -0.0100 | -0.0096 | -0.0098 | -0.0103 |
| 0.75 | -0.0176 | -0.0178 | -0.0177 | -0.0178 | -0.0178 | -0.0178 | -0.0167 | -0.0181 | -0.0175 |
| 0.8 | -0.0273 | -0.0276 | -0.0272 | -0.0272 | -0.0271 | -0.0272 | -0.0262 | -0.0252 | -0.0286 |
| 0.85 | -0.0255 | -0.0256 | -0.0257 | -0.0259 | -0.0260 | -0.0258 | -0.0278 | -0.0277 | -0.0241 |
| 0.9 | -0.0260 | -0.0260 | -0.0259 | -0.0261 | -0.0263 | -0.0261 | -0.0259 | -0.0253 | -0.0279 |
| 0.95 | -0.0469 | -0.0475 | -0.0473 | -0.0480 | -0.0487 | -0.0485 | -0.0513 | -0.0555 | -0.0492 |
| women |  |  |  |  |  |  |  |  |  |
| 0.05 | -0.0050 | -0.0049 | -0.0050 | -0.0049 | -0.0049 | -0.0050 | -0.0049 | -0.0045 | -0.0048 |
| 0.1 | 0.0068 | 0.0068 | 0.0068 | 0.0068 | 0.0068 | 0.0068 | 0.0066 | 0.0071 | 0.0068 |
| 0.15 | 0.0094 | 0.0094 | 0.0094 | 0.0094 | 0.0094 | 0.0094 | 0.0098 | 0.0091 | 0.0093 |
| 0.2 | 0.0058 | 0.0057 | 0.0058 | 0.0058 | 0.0057 | 0.0058 | 0.0059 | 0.0058 | 0.0058 |
| 0.25 | 0.0051 | 0.0051 | 0.0051 | 0.0051 | 0.0050 | 0.0051 | 0.0051 | 0.0050 | 0.0049 |
| 0.3 | 0.0074 | 0.0074 | 0.0074 | 0.0074 | 0.0074 | 0.0075 | 0.0071 | 0.0071 | 0.0072 |
| 0.35 | 0.0015 | 0.0014 | 0.0015 | 0.0015 | 0.0014 | 0.0015 | 0.0014 | 0.0014 | 0.0015 |
| 0.4 | -0.0024 | -0.0024 | -0.0024 | -0.0024 | -0.0024 | -0.0024 | -0.0023 | -0.0024 | -0.0024 |
| 0.45 | 0.0010 | 0.0010 | 0.0010 | 0.0010 | 0.0010 | 0.0010 | 0.0010 | 0.0010 | 0.0010 |
| 0.5 | 0.0051 | 0.0051 | 0.0052 | 0.0052 | 0.0052 | 0.0052 | 0.0051 | 0.0054 | 0.0052 |
| 0.55 | 0.0048 | 0.0047 | 0.0048 | 0.0047 | 0.0047 | 0.0047 | 0.0046 | 0.0046 | 0.0048 |
| 0.6 | -0.0050 | -0.0050 | -0.0050 | -0.0050 | -0.0051 | -0.0050 | -0.0049 | -0.0050 | -0.0049 |
| 0.65 | -0.0046 | -0.0046 | -0.0046 | -0.0046 | -0.0047 | -0.0046 | -0.0047 | -0.0046 | -0.0049 |
| 0.7 | -0.0134 | -0.0133 | -0.0134 | -0.0133 | -0.0132 | -0.0133 | -0.0124 | -0.0123 | -0.0128 |
| 0.75 | -0.0034 | -0.0034 | -0.0034 | -0.0034 | -0.0034 | -0.0034 | -0.0035 | -0.0033 | -0.0032 |
| 0.8 | -0.0164 | -0.0164 | -0.0162 | -0.0163 | -0.0163 | -0.0162 | -0.0160 | -0.0167 | -0.0164 |
| 0.85 | -0.0216 | -0.0215 | -0.0217 | -0.0217 | -0.0217 | -0.0217 | -0.0208 | -0.0189 | -0.0219 |
| 0.9 | -0.0005 | -0.0005 | -0.0005 | -0.0005 | -0.0005 | -0.0005 | -0.0005 | -0.0005 | -0.0005 |
| 0.95 | -0.0322 | -0.0324 | -0.0322 | -0.0326 | -0.0331 | -0.0326 | -0.0376 | -0.0353 | -0.0335 |
| Income 75 |  |  |  |  |  |  |  |  |  |
| men |  |  |  |  |  |  |  |  |  |
| 0.05 | 0.0236 | 0.0234 | 0.0236 | 0.0234 | 0.0234 | 0.0234 | 0.0238 | 0.0227 | 0.0222 |
| 0.1 | 0.0190 | 0.0192 | 0.0188 | 0.0189 | 0.0191 | 0.0188 | 0.0197 | 0.0193 | 0.0193 |
| 0.15 | 0.0064 | 0.0064 | 0.0064 | 0.0064 | 0.0064 | 0.0064 | 0.0063 | 0.0065 | 0.0065 |
| 0.2 | 0.0014 | 0.0014 | 0.0014 | 0.0014 | 0.0014 | 0.0014 | 0.0014 | 0.0014 | 0.0015 |
| 0.25 | -0.0048 | -0.0047 | -0.0048 | -0.0048 | -0.0047 | -0.0048 | -0.0047 | -0.0049 | -0.0046 |
| 0.3 | 0.0003 | 0.0003 | 0.0003 | 0.0003 | 0.0003 | 0.0003 | 0.0002 | 0.0003 | 0.0003 |
| 0.35 | -0.0001 | -0.0001 | -0.0001 | -0.0001 | -0.0001 | -0.0001 | -0.0001 | -0.0001 | -0.0001 |
| 0.4 | -0.0039 | -0.0039 | -0.0039 | -0.0040 | -0.0040 | -0.0040 | -0.0039 | -0.0041 | -0.0039 |
| 0.45 | -0.0018 | -0.0018 | -0.0018 | -0.0018 | -0.0018 | -0.0018 | -0.0017 | -0.0018 | -0.0018 |
| 0.5 | -0.0080 | -0.0079 | -0.0081 | -0.0079 | -0.0078 | -0.0080 | -0.0076 | -0.0076 | -0.0078 |
| 0.55 | -0.0113 | -0.0114 | -0.0114 | -0.0114 | -0.0113 | -0.0114 | -0.0113 | -0.0111 | -0.0113 |
| 0.6 | -0.0162 | -0.0164 | -0.0161 | -0.0164 | -0.0165 | -0.0163 | -0.0166 | -0.0187 | -0.0153 |
| 0.65 | -0.0124 | -0.0124 | -0.0125 | -0.0123 | -0.0123 | -0.0124 | -0.0133 | -0.0128 | -0.0115 |
| 0.7 | -0.0132 | -0.0131 | -0.0133 | -0.0132 | -0.0132 | -0.0132 | -0.0127 | -0.0129 | -0.0135 |
| 0.75 | -0.0122 | -0.0123 | -0.0123 | -0.0123 | -0.0124 | -0.0123 | -0.0116 | -0.0125 | -0.0121 |
| 0.8 | -0.0230 | -0.0233 | -0.0230 | -0.0229 | -0.0229 | -0.0230 | -0.0221 | -0.0212 | -0.0241 |
| 0.85 | -0.0238 | -0.0239 | -0.0239 | -0.0241 | -0.0243 | -0.0241 | -0.0260 | -0.0259 | -0.0224 |
| 0.9 | -0.0227 | -0.0227 | -0.0227 | -0.0229 | -0.0230 | -0.0228 | -0.0227 | -0.0221 | -0.0244 |
| 0.95 | -0.0492 | -0.0497 | -0.0496 | -0.0503 | -0.0510 | -0.0508 | -0.0537 | -0.0581 | -0.0515 |
| women |  |  |  |  |  |  |  |  |  |
| 0.05 | 0.0026 | 0.0026 | 0.0026 | 0.0026 | 0.0026 | 0.0026 | 0.0026 | 0.0024 | 0.0025 |
| 0.1 | 0.0073 | 0.0072 | 0.0073 | 0.0073 | 0.0073 | 0.0073 | 0.0071 | 0.0075 | 0.0073 |
| 0.15 | 0.0070 | 0.0070 | 0.0069 | 0.0070 | 0.0069 | 0.0070 | 0.0073 | 0.0068 | 0.0069 |
| 0.2 | -0.0016 | -0.0016 | -0.0016 | -0.0016 | -0.0016 | -0.0016 | -0.0016 | -0.0016 | -0.0016 |
| 0.25 | -0.0042 | -0.0042 | -0.0042 | -0.0042 | -0.0042 | -0.0042 | -0.0042 | -0.0041 | -0.0041 |
| 0.3 | -0.0069 | -0.0069 | -0.0069 | -0.0069 | -0.0069 | -0.0070 | -0.0066 | -0.0066 | -0.0068 |
| 0.35 | -0.0127 | -0.0126 | -0.0128 | -0.0127 | -0.0126 | -0.0128 | -0.0127 | -0.0123 | -0.0129 |
| 0.4 | -0.0250 | -0.0249 | -0.0250 | -0.0249 | -0.0248 | -0.0250 | -0.0238 | -0.0246 | -0.0246 |
| 0.45 | -0.0335 | -0.0335 | -0.0334 | -0.0336 | -0.0336 | -0.0336 | -0.0324 | -0.0349 | -0.0337 |
| 0.5 | -0.0414 | -0.0412 | -0.0415 | -0.0415 | -0.0415 | -0.0415 | -0.0407 | -0.0434 | -0.0421 |
| 0.55 | -0.0453 | -0.0451 | -0.0455 | -0.0451 | -0.0448 | -0.0452 | -0.0440 | -0.0438 | -0.0454 |
| 0.6 | -0.0571 | -0.0572 | -0.0570 | -0.0572 | -0.0572 | -0.0572 | -0.0555 | -0.0561 | -0.0561 |
| 0.65 | -0.0506 | -0.0507 | -0.0506 | -0.0509 | -0.0511 | -0.0509 | -0.0518 | -0.0501 | -0.0541 |
| 0.7 | -0.0576 | -0.0572 | -0.0577 | -0.0570 | -0.0567 | -0.0572 | -0.0534 | -0.0528 | -0.0552 |
| 0.75 | -0.0562 | -0.0561 | -0.0563 | -0.0564 | -0.0562 | -0.0565 | -0.0574 | -0.0549 | -0.0530 |
| 0.8 | -0.0745 | -0.0745 | -0.0735 | -0.0739 | -0.0741 | -0.0738 | -0.0726 | -0.0758 | -0.0746 |
| 0.85 | -0.0761 | -0.0758 | -0.0763 | -0.0764 | -0.0765 | -0.0763 | -0.0734 | -0.0665 | -0.0771 |
| 0.9 | -0.0637 | -0.0633 | -0.0640 | -0.0643 | -0.0644 | -0.0643 | -0.0594 | -0.0627 | -0.0628 |
| 0.95 | -0.0690 | -0.0696 | -0.0691 | -0.0700 | -0.0711 | -0.0706 | -0.0807 | -0.0758 | -0.0719 |
| Income 90 |  |  |  |  |  |  |  |  |  |
| men |  |  |  |  |  |  |  |  |  |
| 0.05 | 0.0135 | 0.0134 | 0.0135 | 0.0134 | 0.0134 | 0.0134 | 0.0136 | 0.0130 | 0.0127 |
| 0.1 | 0.0088 | 0.0089 | 0.0087 | 0.0088 | 0.0088 | 0.0087 | 0.0091 | 0.0089 | 0.0089 |
| 0.15 | -0.0031 | -0.0031 | -0.0031 | -0.0031 | -0.0031 | -0.0031 | -0.0031 | -0.0032 | -0.0032 |
| 0.2 | -0.0104 | -0.0105 | -0.0104 | -0.0104 | -0.0104 | -0.0105 | -0.0106 | -0.0103 | -0.0111 |
| 0.25 | -0.0131 | -0.0129 | -0.0133 | -0.0131 | -0.0130 | -0.0132 | -0.0130 | -0.0135 | -0.0126 |
| 0.3 | -0.0135 | -0.0134 | -0.0137 | -0.0134 | -0.0133 | -0.0136 | -0.0126 | -0.0134 | -0.0129 |
| 0.35 | -0.0163 | -0.0162 | -0.0163 | -0.0162 | -0.0161 | -0.0163 | -0.0168 | -0.0166 | -0.0158 |
| 0.4 | -0.0184 | -0.0185 | -0.0184 | -0.0186 | -0.0187 | -0.0186 | -0.0184 | -0.0190 | -0.0184 |
| 0.45 | -0.0194 | -0.0193 | -0.0196 | -0.0194 | -0.0193 | -0.0195 | -0.0186 | -0.0193 | -0.0195 |
| 0.5 | -0.0265 | -0.0260 | -0.0268 | -0.0261 | -0.0257 | -0.0263 | -0.0252 | -0.0249 | -0.0256 |
| 0.55 | -0.0287 | -0.0288 | -0.0288 | -0.0288 | -0.0287 | -0.0289 | -0.0286 | -0.0282 | -0.0286 |
| 0.6 | -0.0391 | -0.0395 | -0.0390 | -0.0396 | -0.0399 | -0.0394 | -0.0401 | -0.0451 | -0.0369 |
| 0.65 | -0.0405 | -0.0402 | -0.0407 | -0.0401 | -0.0399 | -0.0402 | -0.0432 | -0.0418 | -0.0374 |
| 0.7 | -0.0400 | -0.0397 | -0.0402 | -0.0400 | -0.0400 | -0.0401 | -0.0385 | -0.0390 | -0.0410 |
| 0.75 | -0.0405 | -0.0408 | -0.0407 | -0.0409 | -0.0410 | -0.0409 | -0.0384 | -0.0416 | -0.0402 |
| 0.8 | -0.0465 | -0.0470 | -0.0464 | -0.0463 | -0.0462 | -0.0464 | -0.0447 | -0.0428 | -0.0488 |
| 0.85 | -0.0478 | -0.0481 | -0.0481 | -0.0485 | -0.0488 | -0.0483 | -0.0521 | -0.0520 | -0.0451 |
| 0.9 | -0.0472 | -0.0472 | -0.0471 | -0.0475 | -0.0477 | -0.0474 | -0.0471 | -0.0459 | -0.0508 |
| 0.95 | -0.0662 | -0.0670 | -0.0667 | -0.0678 | -0.0688 | -0.0690 | -0.0723 | -0.0783 | -0.0694 |
| women |  |  |  |  |  |  |  |  |  |
| 0.05 | -0.0169 | -0.0168 | -0.0170 | -0.0169 | -0.0168 | -0.0169 | -0.0167 | -0.0155 | -0.0164 |
| 0.1 | -0.0157 | -0.0156 | -0.0158 | -0.0157 | -0.0157 | -0.0157 | -0.0153 | -0.0163 | -0.0157 |
| 0.15 | -0.0267 | -0.0268 | -0.0267 | -0.0267 | -0.0267 | -0.0267 | -0.0280 | -0.0260 | -0.0265 |
| 0.2 | -0.0344 | -0.0342 | -0.0345 | -0.0344 | -0.0343 | -0.0345 | -0.0352 | -0.0345 | -0.0347 |
| 0.25 | -0.0344 | -0.0343 | -0.0344 | -0.0344 | -0.0342 | -0.0344 | -0.0344 | -0.0338 | -0.0333 |
| 0.3 | -0.0387 | -0.0386 | -0.0389 | -0.0388 | -0.0387 | -0.0389 | -0.0371 | -0.0369 | -0.0378 |
| 0.35 | -0.0376 | -0.0373 | -0.0378 | -0.0375 | -0.0373 | -0.0377 | -0.0374 | -0.0363 | -0.0381 |
| 0.4 | -0.0535 | -0.0533 | -0.0537 | -0.0534 | -0.0531 | -0.0535 | -0.0509 | -0.0527 | -0.0528 |
| 0.45 | -0.0582 | -0.0583 | -0.0581 | -0.0584 | -0.0584 | -0.0585 | -0.0563 | -0.0607 | -0.0585 |
| 0.5 | -0.0606 | -0.0604 | -0.0608 | -0.0608 | -0.0608 | -0.0609 | -0.0596 | -0.0635 | -0.0617 |
| 0.55 | -0.0692 | -0.0689 | -0.0694 | -0.0688 | -0.0684 | -0.0690 | -0.0671 | -0.0669 | -0.0693 |
| 0.6 | -0.0774 | -0.0775 | -0.0772 | -0.0775 | -0.0775 | -0.0775 | -0.0752 | -0.0760 | -0.0760 |
| 0.65 | -0.0819 | -0.0819 | -0.0818 | -0.0824 | -0.0825 | -0.0823 | -0.0837 | -0.0809 | -0.0875 |
| 0.7 | -0.0922 | -0.0915 | -0.0924 | -0.0913 | -0.0907 | -0.0916 | -0.0855 | -0.0846 | -0.0883 |
| 0.75 | -0.0878 | -0.0877 | -0.0881 | -0.0881 | -0.0879 | -0.0883 | -0.0898 | -0.0858 | -0.0829 |
| 0.8 | -0.1096 | -0.1096 | -0.1081 | -0.1087 | -0.1090 | -0.1085 | -0.1068 | -0.1115 | -0.1098 |
| 0.85 | -0.1025 | -0.1021 | -0.1028 | -0.1028 | -0.1030 | -0.1028 | -0.0989 | -0.0895 | -0.1039 |
| 0.9 | -0.0852 | -0.0847 | -0.0855 | -0.0859 | -0.0861 | -0.0859 | -0.0794 | -0.0839 | -0.0839 |
| 0.95 | -0.0869 | -0.0877 | -0.0870 | -0.0882 | -0.0895 | -0.0887 | -0.1016 | -0.0954 | -0.0905 |
| Income 100 |  |  |  |  |  |  |  |  |  |
| men |  |  |  |  |  |  |  |  |  |
| 0.05 | 0.0375 | 0.0372 | 0.0374 | 0.0371 | 0.0370 | 0.0371 | 0.0377 | 0.0360 | 0.0352 |
| 0.1 | 0.0304 | 0.0306 | 0.0300 | 0.0303 | 0.0305 | 0.0300 | 0.0315 | 0.0309 | 0.0308 |
| 0.15 | 0.0065 | 0.0065 | 0.0065 | 0.0065 | 0.0065 | 0.0065 | 0.0064 | 0.0066 | 0.0066 |
| 0.2 | 0.0005 | 0.0005 | 0.0005 | 0.0005 | 0.0005 | 0.0005 | 0.0005 | 0.0005 | 0.0005 |
| 0.25 | -0.0102 | -0.0100 | -0.0103 | -0.0102 | -0.0101 | -0.0103 | -0.0101 | -0.0105 | -0.0098 |
| 0.3 | -0.0115 | -0.0114 | -0.0116 | -0.0114 | -0.0113 | -0.0116 | -0.0107 | -0.0114 | -0.0110 |
| 0.35 | -0.0213 | -0.0212 | -0.0214 | -0.0213 | -0.0212 | -0.0214 | -0.0221 | -0.0217 | -0.0207 |
| 0.4 | -0.0326 | -0.0329 | -0.0327 | -0.0331 | -0.0332 | -0.0331 | -0.0326 | -0.0338 | -0.0327 |
| 0.45 | -0.0311 | -0.0309 | -0.0313 | -0.0311 | -0.0309 | -0.0313 | -0.0298 | -0.0309 | -0.0312 |
| 0.5 | -0.0382 | -0.0375 | -0.0386 | -0.0376 | -0.0371 | -0.0370 | -0.0363 | -0.0359 | -0.0369 |
| 0.55 | -0.0392 | -0.0395 | -0.0394 | -0.0394 | -0.0393 | -0.0395 | -0.0391 | -0.0386 | -0.0391 |
| 0.6 | -0.0557 | -0.0563 | -0.0556 | -0.0564 | -0.0569 | -0.0562 | -0.0571 | -0.0643 | -0.0526 |
| 0.65 | -0.0508 | -0.0505 | -0.0510 | -0.0503 | -0.0501 | -0.0505 | -0.0542 | -0.0524 | -0.0469 |
| 0.7 | -0.0470 | -0.0467 | -0.0472 | -0.0471 | -0.0470 | -0.0472 | -0.0452 | -0.0458 | -0.0482 |
| 0.75 | -0.0392 | -0.0394 | -0.0393 | -0.0395 | -0.0396 | -0.0396 | -0.0371 | -0.0402 | -0.0388 |
| 0.8 | -0.0435 | -0.0439 | -0.0433 | -0.0433 | -0.0431 | -0.0433 | -0.0418 | -0.0400 | -0.0456 |
| 0.85 | -0.0485 | -0.0488 | -0.0489 | -0.0493 | -0.0496 | -0.0491 | -0.0530 | -0.0528 | -0.0458 |
| 0.9 | -0.0441 | -0.0441 | -0.0440 | -0.0443 | -0.0445 | -0.0442 | -0.0439 | -0.0429 | -0.0474 |
| 0.95 | -0.0691 | -0.0699 | -0.0696 | -0.0707 | -0.0717 | -0.0719 | -0.0754 | -0.0816 | -0.0724 |
| women |  |  |  |  |  |  |  |  |  |
| 0.05 | -0.0219 | -0.0217 | -0.0220 | -0.0218 | -0.0218 | -0.0219 | -0.0216 | -0.0200 | -0.0213 |
| 0.1 | -0.0445 | -0.0442 | -0.0446 | -0.0445 | -0.0444 | -0.0445 | -0.0433 | -0.0462 | -0.0445 |
| 0.15 | -0.0640 | -0.0642 | -0.0638 | -0.0640 | -0.0639 | -0.0640 | -0.0670 | -0.0623 | -0.0635 |
| 0.2 | -0.0640 | -0.0636 | -0.0642 | -0.0639 | -0.0637 | -0.0640 | -0.0655 | -0.0642 | -0.0644 |
| 0.25 | -0.0742 | -0.0739 | -0.0741 | -0.0741 | -0.0738 | -0.0742 | -0.0740 | -0.0728 | -0.0717 |
| 0.3 | -0.0853 | -0.0850 | -0.0856 | -0.0855 | -0.0852 | -0.0857 | -0.0817 | -0.0812 | -0.0833 |
| 0.35 | -0.0799 | -0.0792 | -0.0802 | -0.0797 | -0.0792 | -0.0800 | -0.0795 | -0.0770 | -0.0809 |
| 0.4 | -0.0932 | -0.0928 | -0.0934 | -0.0929 | -0.0924 | -0.0931 | -0.0887 | -0.0918 | -0.0918 |
| 0.45 | -0.1030 | -0.1032 | -0.1029 | -0.1035 | -0.1034 | -0.1035 | -0.0996 | -0.1074 | -0.1036 |
| 0.5 | -0.0971 | -0.0967 | -0.0974 | -0.0974 | -0.0973 | -0.0975 | -0.0954 | -0.1018 | -0.0988 |
| 0.55 | -0.1025 | -0.1021 | -0.1028 | -0.1020 | -0.1014 | -0.1022 | -0.0994 | -0.0991 | -0.1027 |
| 0.6 | -0.1114 | -0.1116 | -0.1112 | -0.1116 | -0.1116 | -0.1116 | -0.1083 | -0.1094 | -0.1094 |
| 0.65 | -0.1138 | -0.1139 | -0.1138 | -0.1145 | -0.1148 | -0.1145 | -0.1163 | -0.1125 | -0.1216 |
| 0.7 | -0.1250 | -0.1240 | -0.1253 | -0.1238 | -0.1230 | -0.1241 | -0.1159 | -0.1147 | -0.1197 |
| 0.75 | -0.1198 | -0.1197 | -0.1202 | -0.1202 | -0.1199 | -0.1204 | -0.1225 | -0.1171 | -0.1131 |
| 0.8 | -0.1367 | -0.1366 | -0.1348 | -0.1356 | -0.1359 | -0.1353 | -0.1332 | -0.1390 | -0.1369 |
| 0.85 | -0.1407 | -0.1401 | -0.1411 | -0.1411 | -0.1414 | -0.1410 | -0.1357 | -0.1228 | -0.1426 |
| 0.9 | -0.1075 | -0.1069 | -0.1080 | -0.1085 | -0.1088 | -0.1085 | -0.1002 | -0.1059 | -0.1059 |
| 0.95 | -0.1039 | -0.1047 | -0.1039 | -0.1054 | -0.1069 | -0.1068 | -0.1214 | -0.1140 | -0.1082 |
| Primary |  |  |  |  |  |  |  |  |  |
| men |  |  |  |  |  |  |  |  |  |
| 0.05 | -0.0098 | -0.0097 | -0.0098 | -0.0097 | -0.0097 | -0.0097 | -0.0099 | -0.0094 | -0.0092 |
| 0.1 | -0.0045 | -0.0046 | -0.0045 | -0.0045 | -0.0046 | -0.0045 | -0.0047 | -0.0046 | -0.0046 |
| 0.15 | -0.0018 | -0.0018 | -0.0018 | -0.0018 | -0.0018 | -0.0018 | -0.0017 | -0.0018 | -0.0018 |
| 0.2 | 0.0006 | 0.0006 | 0.0006 | 0.0006 | 0.0006 | 0.0006 | 0.0006 | 0.0005 | 0.0006 |
| 0.25 | -0.0001 | -0.0001 | -0.0001 | -0.0001 | -0.0001 | -0.0001 | -0.0001 | -0.0001 | -0.0001 |
| 0.3 | 0.0017 | 0.0017 | 0.0017 | 0.0017 | 0.0017 | 0.0017 | 0.0016 | 0.0017 | 0.0016 |
| 0.35 | 0.0044 | 0.0044 | 0.0044 | 0.0044 | 0.0043 | 0.0044 | 0.0045 | 0.0045 | 0.0043 |
| 0.4 | -0.0001 | -0.0001 | -0.0001 | -0.0001 | -0.0001 | -0.0001 | -0.0001 | -0.0001 | -0.0001 |
| 0.45 | -0.0003 | -0.0003 | -0.0003 | -0.0003 | -0.0003 | -0.0003 | -0.0003 | -0.0003 | -0.0003 |
| 0.5 | -0.0040 | -0.0039 | -0.0040 | -0.0039 | -0.0038 | -0.0039 | -0.0038 | -0.0037 | -0.0038 |
| 0.55 | -0.0053 | -0.0053 | -0.0053 | -0.0053 | -0.0053 | -0.0053 | -0.0052 | -0.0052 | -0.0052 |
| 0.6 | -0.0069 | -0.0070 | -0.0069 | -0.0070 | -0.0071 | -0.0070 | -0.0071 | -0.0080 | -0.0065 |
| 0.65 | -0.0009 | -0.0009 | -0.0009 | -0.0009 | -0.0009 | -0.0009 | -0.0009 | -0.0009 | -0.0008 |
| 0.7 | -0.0046 | -0.0045 | -0.0046 | -0.0046 | -0.0045 | -0.0046 | -0.0044 | -0.0044 | -0.0047 |
| 0.75 | -0.0045 | -0.0046 | -0.0045 | -0.0046 | -0.0046 | -0.0046 | -0.0043 | -0.0046 | -0.0045 |
| 0.8 | -0.0098 | -0.0099 | -0.0097 | -0.0097 | -0.0097 | -0.0097 | -0.0094 | -0.0090 | -0.0102 |
| 0.85 | -0.0137 | -0.0138 | -0.0138 | -0.0140 | -0.0140 | -0.0139 | -0.0150 | -0.0150 | -0.0130 |
| 0.9 | -0.0245 | -0.0245 | -0.0245 | -0.0247 | -0.0248 | -0.0246 | -0.0245 | -0.0239 | -0.0264 |
| 0.95 | -0.0385 | -0.0390 | -0.0388 | -0.0394 | -0.0400 | -0.0390 | -0.0421 | -0.0455 | -0.0404 |
| women |  |  |  |  |  |  |  |  |  |
| 0.05 | 0.0021 | 0.0021 | 0.0021 | 0.0021 | 0.0021 | 0.0021 | 0.0021 | 0.0019 | 0.0020 |
| 0.1 | -0.0001 | -0.0001 | -0.0001 | -0.0001 | -0.0001 | -0.0001 | -0.0001 | -0.0001 | -0.0001 |
| 0.15 | -0.0010 | -0.0010 | -0.0010 | -0.0010 | -0.0010 | -0.0010 | -0.0010 | -0.0009 | -0.0010 |
| 0.2 | -0.0054 | -0.0053 | -0.0054 | -0.0054 | -0.0054 | -0.0054 | -0.0055 | -0.0054 | -0.0054 |
| 0.25 | -0.0132 | -0.0132 | -0.0132 | -0.0132 | -0.0132 | -0.0133 | -0.0132 | -0.0130 | -0.0128 |
| 0.3 | -0.0159 | -0.0158 | -0.0159 | -0.0159 | -0.0159 | -0.0160 | -0.0152 | -0.0151 | -0.0155 |
| 0.35 | -0.0159 | -0.0157 | -0.0159 | -0.0159 | -0.0158 | -0.0159 | -0.0158 | -0.0153 | -0.0161 |
| 0.4 | -0.0182 | -0.0181 | -0.0182 | -0.0181 | -0.0180 | -0.0181 | -0.0173 | -0.0179 | -0.0179 |
| 0.45 | -0.0216 | -0.0217 | -0.0216 | -0.0217 | -0.0217 | -0.0217 | -0.0209 | -0.0225 | -0.0217 |
| 0.5 | -0.0230 | -0.0229 | -0.0230 | -0.0230 | -0.0230 | -0.0231 | -0.0226 | -0.0241 | -0.0234 |
| 0.55 | -0.0296 | -0.0295 | -0.0297 | -0.0295 | -0.0293 | -0.0295 | -0.0287 | -0.0287 | -0.0297 |
| 0.6 | -0.0310 | -0.0310 | -0.0309 | -0.0310 | -0.0310 | -0.0310 | -0.0301 | -0.0304 | -0.0304 |
| 0.65 | -0.0344 | -0.0344 | -0.0344 | -0.0346 | -0.0347 | -0.0346 | -0.0352 | -0.0340 | -0.0368 |
| 0.7 | -0.0420 | -0.0417 | -0.0421 | -0.0416 | -0.0413 | -0.0417 | -0.0389 | -0.0385 | -0.0402 |
| 0.75 | -0.0521 | -0.0521 | -0.0523 | -0.0523 | -0.0522 | -0.0524 | -0.0533 | -0.0509 | -0.0492 |
| 0.8 | -0.0665 | -0.0665 | -0.0656 | -0.0659 | -0.0661 | -0.0658 | -0.0648 | -0.0676 | -0.0666 |
| 0.85 | -0.0721 | -0.0718 | -0.0723 | -0.0723 | -0.0725 | -0.0723 | -0.0695 | -0.0630 | -0.0731 |
| 0.9 | -0.0703 | -0.0699 | -0.0707 | -0.0710 | -0.0711 | -0.0710 | -0.0655 | -0.0693 | -0.0693 |
| 0.95 | -0.0701 | -0.0707 | -0.0702 | -0.0711 | -0.0722 | -0.0717 | -0.0820 | -0.0769 | -0.0730 |
| Secondary |  |  |  |  |  |  |  |  |  |
| men |  |  |  |  |  |  |  |  |  |
| 0.05 | 0.0030 | 0.0030 | 0.0030 | 0.0030 | 0.0030 | 0.0030 | 0.0030 | 0.0029 | 0.0028 |
| 0.1 | 0.0065 | 0.0065 | 0.0064 | 0.0064 | 0.0065 | 0.0064 | 0.0067 | 0.0066 | 0.0066 |
| 0.15 | 0.0011 | 0.0011 | 0.0011 | 0.0011 | 0.0011 | 0.0011 | 0.0011 | 0.0011 | 0.0011 |
| 0.2 | 0.0012 | 0.0012 | 0.0012 | 0.0012 | 0.0012 | 0.0012 | 0.0012 | 0.0012 | 0.0013 |
| 0.25 | -0.0032 | -0.0032 | -0.0033 | -0.0032 | -0.0032 | -0.0032 | -0.0032 | -0.0033 | -0.0031 |
| 0.3 | -0.0037 | -0.0037 | -0.0038 | -0.0037 | -0.0037 | -0.0038 | -0.0035 | -0.0037 | -0.0036 |
| 0.35 | -0.0023 | -0.0023 | -0.0024 | -0.0023 | -0.0023 | -0.0024 | -0.0024 | -0.0024 | -0.0023 |
| 0.4 | -0.0088 | -0.0089 | -0.0089 | -0.0090 | -0.0090 | -0.0090 | -0.0088 | -0.0092 | -0.0089 |
| 0.45 | -0.0093 | -0.0093 | -0.0094 | -0.0093 | -0.0093 | -0.0094 | -0.0089 | -0.0093 | -0.0093 |
| 0.5 | -0.0109 | -0.0107 | -0.0111 | -0.0108 | -0.0106 | -0.0109 | -0.0104 | -0.0103 | -0.0106 |
| 0.55 | -0.0164 | -0.0165 | -0.0165 | -0.0165 | -0.0164 | -0.0165 | -0.0164 | -0.0161 | -0.0164 |
| 0.6 | -0.0229 | -0.0231 | -0.0228 | -0.0231 | -0.0233 | -0.0231 | -0.0234 | -0.0264 | -0.0216 |
| 0.65 | -0.0207 | -0.0206 | -0.0208 | -0.0205 | -0.0204 | -0.0206 | -0.0221 | -0.0214 | -0.0191 |
| 0.7 | -0.0266 | -0.0264 | -0.0267 | -0.0266 | -0.0266 | -0.0267 | -0.0256 | -0.0259 | -0.0273 |
| 0.75 | -0.0283 | -0.0285 | -0.0284 | -0.0286 | -0.0286 | -0.0286 | -0.0268 | -0.0291 | -0.0281 |
| 0.8 | -0.0376 | -0.0380 | -0.0374 | -0.0374 | -0.0373 | -0.0374 | -0.0361 | -0.0346 | -0.0394 |
| 0.85 | -0.0361 | -0.0363 | -0.0364 | -0.0367 | -0.0369 | -0.0366 | -0.0394 | -0.0393 | -0.0341 |
| 0.9 | -0.0499 | -0.0499 | -0.0498 | -0.0502 | -0.0504 | -0.0501 | -0.0497 | -0.0485 | -0.0536 |
| 0.95 | -0.0591 | -0.0597 | -0.0595 | -0.0604 | -0.0613 | -0.0608 | -0.0645 | -0.0698 | -0.0619 |
| women |  |  |  |  |  |  |  |  |  |
| 0.05 | -0.0139 | -0.0138 | -0.0139 | -0.0138 | -0.0138 | -0.0139 | -0.0137 | -0.0127 | -0.0135 |
| 0.1 | -0.0222 | -0.0220 | -0.0223 | -0.0222 | -0.0222 | -0.0222 | -0.0216 | -0.0230 | -0.0222 |
| 0.15 | -0.0295 | -0.0296 | -0.0294 | -0.0295 | -0.0294 | -0.0295 | -0.0308 | -0.0287 | -0.0292 |
| 0.2 | -0.0406 | -0.0404 | -0.0407 | -0.0406 | -0.0404 | -0.0406 | -0.0416 | -0.0407 | -0.0409 |
| 0.25 | -0.0474 | -0.0473 | -0.0474 | -0.0474 | -0.0472 | -0.0475 | -0.0473 | -0.0466 | -0.0459 |
| 0.3 | -0.0574 | -0.0572 | -0.0576 | -0.0576 | -0.0574 | -0.0577 | -0.0550 | -0.0547 | -0.0561 |
| 0.35 | -0.0596 | -0.0591 | -0.0598 | -0.0595 | -0.0591 | -0.0596 | -0.0593 | -0.0574 | -0.0603 |
| 0.4 | -0.0693 | -0.0690 | -0.0695 | -0.0691 | -0.0687 | -0.0693 | -0.0660 | -0.0683 | -0.0683 |
| 0.45 | -0.0774 | -0.0775 | -0.0773 | -0.0777 | -0.0777 | -0.0778 | -0.0748 | -0.0806 | -0.0778 |
| 0.5 | -0.0798 | -0.0795 | -0.0801 | -0.0801 | -0.0800 | -0.0801 | -0.0784 | -0.0836 | -0.0812 |
| 0.55 | -0.0935 | -0.0931 | -0.0937 | -0.0930 | -0.0924 | -0.0932 | -0.0906 | -0.0903 | -0.0936 |
| 0.6 | -0.0989 | -0.0991 | -0.0987 | -0.0990 | -0.0991 | -0.0990 | -0.0961 | -0.0971 | -0.0971 |
| 0.65 | -0.1005 | -0.1005 | -0.1004 | -0.1011 | -0.1013 | -0.1010 | -0.1027 | -0.0993 | -0.1074 |
| 0.7 | -0.1099 | -0.1090 | -0.1101 | -0.1088 | -0.1081 | -0.1091 | -0.1018 | -0.1008 | -0.1053 |
| 0.75 | -0.1197 | -0.1196 | -0.1201 | -0.1202 | -0.1199 | -0.1204 | -0.1224 | -0.1170 | -0.1130 |
| 0.8 | -0.1378 | -0.1377 | -0.1359 | -0.1366 | -0.1369 | -0.1364 | -0.1342 | -0.1401 | -0.1380 |
| 0.85 | -0.1480 | -0.1475 | -0.1485 | -0.1485 | -0.1488 | -0.1485 | -0.1428 | -0.1293 | -0.1501 |
| 0.9 | -0.1304 | -0.1297 | -0.1310 | -0.1316 | -0.1319 | -0.1315 | -0.1215 | -0.1284 | -0.1285 |
| 0.95 | -0.1267 | -0.1277 | -0.1268 | -0.1285 | -0.1304 | -0.1277 | -0.1481 | -0.1390 | -0.1319 |
| Social Class II |  |  |  |  |  |  |  |  |  |
| men |  |  |  |  |  |  |  |  |  |
| 0.05 | 0.0031 | 0.0031 | 0.0031 | 0.0031 | 0.0031 | 0.0031 | 0.0032 | 0.0030 | 0.0029 |
| 0.1 | -0.0066 | -0.0067 | -0.0065 | -0.0066 | -0.0066 | -0.0065 | -0.0068 | -0.0067 | -0.0067 |
| 0.15 | -0.0049 | -0.0049 | -0.0049 | -0.0049 | -0.0049 | -0.0049 | -0.0048 | -0.0050 | -0.0049 |
| 0.2 | -0.0047 | -0.0047 | -0.0047 | -0.0047 | -0.0047 | -0.0047 | -0.0048 | -0.0047 | -0.0050 |
| 0.25 | -0.0027 | -0.0026 | -0.0027 | -0.0027 | -0.0026 | -0.0027 | -0.0026 | -0.0027 | -0.0026 |
| 0.3 | -0.0015 | -0.0015 | -0.0016 | -0.0015 | -0.0015 | -0.0015 | -0.0014 | -0.0015 | -0.0015 |
| 0.35 | 0.0025 | 0.0025 | 0.0025 | 0.0025 | 0.0025 | 0.0025 | 0.0026 | 0.0026 | 0.0025 |
| 0.4 | 0.0060 | 0.0061 | 0.0061 | 0.0061 | 0.0061 | 0.0061 | 0.0060 | 0.0063 | 0.0061 |
| 0.45 | 0.0049 | 0.0049 | 0.0050 | 0.0049 | 0.0049 | 0.0050 | 0.0047 | 0.0049 | 0.0049 |
| 0.5 | 0.0095 | 0.0094 | 0.0097 | 0.0094 | 0.0093 | 0.0095 | 0.0091 | 0.0090 | 0.0092 |
| 0.55 | 0.0142 | 0.0143 | 0.0142 | 0.0142 | 0.0142 | 0.0143 | 0.0141 | 0.0139 | 0.0141 |
| 0.6 | 0.0106 | 0.0107 | 0.0105 | 0.0107 | 0.0108 | 0.0106 | 0.0108 | 0.0122 | 0.0100 |
| 0.65 | 0.0118 | 0.0118 | 0.0119 | 0.0117 | 0.0117 | 0.0118 | 0.0126 | 0.0122 | 0.0109 |
| 0.7 | 0.0142 | 0.0141 | 0.0142 | 0.0142 | 0.0142 | 0.0142 | 0.0136 | 0.0138 | 0.0145 |
| 0.75 | 0.0119 | 0.0120 | 0.0119 | 0.0120 | 0.0120 | 0.0120 | 0.0112 | 0.0122 | 0.0118 |
| 0.8 | 0.0140 | 0.0141 | 0.0139 | 0.0139 | 0.0138 | 0.0139 | 0.0134 | 0.0128 | 0.0146 |
| 0.85 | 0.0053 | 0.0054 | 0.0054 | 0.0054 | 0.0055 | 0.0054 | 0.0058 | 0.0058 | 0.0050 |
| 0.9 | 0.0008 | 0.0008 | 0.0008 | 0.0008 | 0.0008 | 0.0008 | 0.0008 | 0.0008 | 0.0009 |
| 0.95 | 0.0234 | 0.0237 | 0.0236 | 0.0240 | 0.0243 | 0.0237 | 0.0256 | 0.0277 | 0.0245 |
| women |  |  |  |  |  |  |  |  |  |
| 0.05 | 0.0173 | 0.0171 | 0.0174 | 0.0172 | 0.0172 | 0.0172 | 0.0170 | 0.0158 | 0.0168 |
| 0.1 | 0.0185 | 0.0183 | 0.0185 | 0.0185 | 0.0184 | 0.0185 | 0.0179 | 0.0191 | 0.0185 |
| 0.15 | 0.0340 | 0.0341 | 0.0339 | 0.0340 | 0.0340 | 0.0340 | 0.0356 | 0.0331 | 0.0337 |
| 0.2 | 0.0339 | 0.0336 | 0.0339 | 0.0338 | 0.0337 | 0.0339 | 0.0346 | 0.0339 | 0.0341 |
| 0.25 | 0.0309 | 0.0308 | 0.0308 | 0.0308 | 0.0307 | 0.0309 | 0.0308 | 0.0303 | 0.0299 |
| 0.3 | 0.0334 | 0.0333 | 0.0335 | 0.0335 | 0.0333 | 0.0336 | 0.0320 | 0.0318 | 0.0326 |
| 0.35 | 0.0370 | 0.0367 | 0.0372 | 0.0369 | 0.0367 | 0.0371 | 0.0368 | 0.0357 | 0.0375 |
| 0.4 | 0.0304 | 0.0303 | 0.0305 | 0.0303 | 0.0301 | 0.0304 | 0.0289 | 0.0299 | 0.0299 |
| 0.45 | 0.0264 | 0.0264 | 0.0263 | 0.0265 | 0.0265 | 0.0265 | 0.0255 | 0.0275 | 0.0265 |
| 0.5 | 0.0336 | 0.0335 | 0.0337 | 0.0337 | 0.0337 | 0.0338 | 0.0330 | 0.0352 | 0.0342 |
| 0.55 | 0.0225 | 0.0224 | 0.0226 | 0.0224 | 0.0223 | 0.0225 | 0.0219 | 0.0218 | 0.0226 |
| 0.6 | 0.0171 | 0.0171 | 0.0170 | 0.0171 | 0.0171 | 0.0171 | 0.0166 | 0.0168 | 0.0168 |
| 0.65 | 0.0195 | 0.0195 | 0.0195 | 0.0196 | 0.0197 | 0.0196 | 0.0199 | 0.0193 | 0.0208 |
| 0.7 | 0.0217 | 0.0215 | 0.0217 | 0.0215 | 0.0213 | 0.0215 | 0.0201 | 0.0199 | 0.0208 |
| 0.75 | 0.0249 | 0.0249 | 0.0250 | 0.0250 | 0.0250 | 0.0251 | 0.0255 | 0.0244 | 0.0235 |
| 0.8 | 0.0248 | 0.0248 | 0.0244 | 0.0246 | 0.0246 | 0.0245 | 0.0241 | 0.0252 | 0.0248 |
| 0.85 | 0.0149 | 0.0149 | 0.0150 | 0.0150 | 0.0150 | 0.0150 | 0.0144 | 0.0130 | 0.0151 |
| 0.9 | -0.0006 | -0.0006 | -0.0006 | -0.0006 | -0.0006 | -0.0006 | -0.0005 | -0.0005 | -0.0005 |
| 0.95 | -0.0049 | -0.0049 | -0.0049 | -0.0050 | -0.0050 | -0.0049 | -0.0057 | -0.0054 | -0.0051 |
| Social Class III |  |  |  |  |  |  |  |  |  |
| men |  |  |  |  |  |  |  |  |  |
| 0.05 | 0.0039 | 0.0038 | 0.0039 | 0.0038 | 0.0038 | 0.0038 | 0.0039 | 0.0037 | 0.0036 |
| 0.1 | 0.0029 | 0.0029 | 0.0028 | 0.0028 | 0.0029 | 0.0028 | 0.0030 | 0.0029 | 0.0029 |
| 0.15 | 0.0086 | 0.0086 | 0.0086 | 0.0087 | 0.0087 | 0.0086 | 0.0085 | 0.0088 | 0.0088 |
| 0.2 | 0.0091 | 0.0091 | 0.0091 | 0.0091 | 0.0091 | 0.0092 | 0.0093 | 0.0090 | 0.0097 |
| 0.25 | 0.0079 | 0.0078 | 0.0080 | 0.0079 | 0.0079 | 0.0080 | 0.0079 | 0.0081 | 0.0076 |
| 0.3 | 0.0096 | 0.0094 | 0.0097 | 0.0095 | 0.0094 | 0.0096 | 0.0089 | 0.0095 | 0.0091 |
| 0.35 | 0.0085 | 0.0085 | 0.0086 | 0.0085 | 0.0085 | 0.0086 | 0.0088 | 0.0087 | 0.0083 |
| 0.4 | 0.0113 | 0.0114 | 0.0114 | 0.0115 | 0.0115 | 0.0115 | 0.0113 | 0.0117 | 0.0114 |
| 0.45 | 0.0126 | 0.0125 | 0.0127 | 0.0126 | 0.0125 | 0.0126 | 0.0121 | 0.0125 | 0.0126 |
| 0.5 | 0.0154 | 0.0151 | 0.0156 | 0.0152 | 0.0150 | 0.0153 | 0.0147 | 0.0145 | 0.0149 |
| 0.55 | 0.0154 | 0.0155 | 0.0155 | 0.0154 | 0.0154 | 0.0155 | 0.0153 | 0.0151 | 0.0153 |
| 0.6 | 0.0121 | 0.0122 | 0.0120 | 0.0122 | 0.0123 | 0.0122 | 0.0124 | 0.0139 | 0.0114 |
| 0.65 | 0.0125 | 0.0124 | 0.0125 | 0.0124 | 0.0123 | 0.0124 | 0.0133 | 0.0129 | 0.0115 |
| 0.7 | 0.0165 | 0.0164 | 0.0166 | 0.0166 | 0.0165 | 0.0166 | 0.0159 | 0.0161 | 0.0170 |
| 0.75 | 0.0187 | 0.0188 | 0.0187 | 0.0188 | 0.0189 | 0.0188 | 0.0177 | 0.0191 | 0.0185 |
| 0.8 | 0.0206 | 0.0208 | 0.0206 | 0.0205 | 0.0205 | 0.0206 | 0.0198 | 0.0190 | 0.0216 |
| 0.85 | 0.0187 | 0.0188 | 0.0189 | 0.0190 | 0.0191 | 0.0189 | 0.0204 | 0.0204 | 0.0177 |
| 0.9 | 0.0190 | 0.0190 | 0.0189 | 0.0191 | 0.0192 | 0.0190 | 0.0189 | 0.0184 | 0.0204 |
| 0.95 | 0.0530 | 0.0536 | 0.0534 | 0.0542 | 0.0550 | 0.0546 | 0.0578 | 0.0626 | 0.0555 |
| women |  |  |  |  |  |  |  |  |  |
| 0.05 | 0.0229 | 0.0228 | 0.0231 | 0.0229 | 0.0228 | 0.0229 | 0.0226 | 0.0210 | 0.0223 |
| 0.1 | 0.0318 | 0.0316 | 0.0319 | 0.0318 | 0.0318 | 0.0319 | 0.0309 | 0.0330 | 0.0318 |
| 0.15 | 0.0425 | 0.0426 | 0.0424 | 0.0425 | 0.0424 | 0.0425 | 0.0445 | 0.0413 | 0.0421 |
| 0.2 | 0.0520 | 0.0517 | 0.0521 | 0.0519 | 0.0518 | 0.0520 | 0.0532 | 0.0521 | 0.0523 |
| 0.25 | 0.0508 | 0.0506 | 0.0507 | 0.0507 | 0.0505 | 0.0508 | 0.0507 | 0.0499 | 0.0491 |
| 0.3 | 0.0514 | 0.0513 | 0.0516 | 0.0516 | 0.0514 | 0.0517 | 0.0493 | 0.0490 | 0.0502 |
| 0.35 | 0.0511 | 0.0507 | 0.0513 | 0.0510 | 0.0507 | 0.0512 | 0.0509 | 0.0493 | 0.0517 |
| 0.4 | 0.0439 | 0.0437 | 0.0440 | 0.0438 | 0.0435 | 0.0439 | 0.0418 | 0.0433 | 0.0433 |
| 0.45 | 0.0481 | 0.0482 | 0.0481 | 0.0484 | 0.0483 | 0.0484 | 0.0465 | 0.0502 | 0.0484 |
| 0.5 | 0.0508 | 0.0506 | 0.0510 | 0.0510 | 0.0509 | 0.0510 | 0.0499 | 0.0532 | 0.0517 |
| 0.55 | 0.0540 | 0.0537 | 0.0541 | 0.0537 | 0.0534 | 0.0538 | 0.0523 | 0.0522 | 0.0540 |
| 0.6 | 0.0476 | 0.0477 | 0.0475 | 0.0477 | 0.0477 | 0.0477 | 0.0462 | 0.0467 | 0.0467 |
| 0.65 | 0.0518 | 0.0518 | 0.0518 | 0.0521 | 0.0522 | 0.0521 | 0.0530 | 0.0512 | 0.0554 |
| 0.7 | 0.0611 | 0.0606 | 0.0612 | 0.0605 | 0.0601 | 0.0606 | 0.0566 | 0.0560 | 0.0585 |
| 0.75 | 0.0627 | 0.0626 | 0.0628 | 0.0629 | 0.0627 | 0.0630 | 0.0641 | 0.0612 | 0.0592 |
| 0.8 | 0.0630 | 0.0630 | 0.0621 | 0.0625 | 0.0626 | 0.0624 | 0.0614 | 0.0641 | 0.0631 |
| 0.85 | 0.0519 | 0.0517 | 0.0520 | 0.0521 | 0.0521 | 0.0520 | 0.0501 | 0.0453 | 0.0526 |
| 0.9 | 0.0394 | 0.0392 | 0.0396 | 0.0398 | 0.0399 | 0.0398 | 0.0367 | 0.0388 | 0.0388 |
| 0.95 | 0.0309 | 0.0311 | 0.0309 | 0.0313 | 0.0318 | 0.0315 | 0.0361 | 0.0339 | 0.0321 |
| Social Class IV |  |  |  |  |  |  |  |  |  |
| men |  |  |  |  |  |  |  |  |  |
| 0.05 | 0.0063 | 0.0063 | 0.0063 | 0.0063 | 0.0063 | 0.0063 | 0.0064 | 0.0061 | 0.0060 |
| 0.1 | 0.0091 | 0.0092 | 0.0090 | 0.0091 | 0.0091 | 0.0090 | 0.0094 | 0.0092 | 0.0092 |
| 0.15 | 0.0183 | 0.0183 | 0.0182 | 0.0183 | 0.0184 | 0.0182 | 0.0180 | 0.0187 | 0.0185 |
| 0.2 | 0.0198 | 0.0199 | 0.0199 | 0.0198 | 0.0198 | 0.0199 | 0.0202 | 0.0195 | 0.0211 |
| 0.25 | 0.0204 | 0.0201 | 0.0207 | 0.0204 | 0.0203 | 0.0205 | 0.0203 | 0.0210 | 0.0196 |
| 0.3 | 0.0240 | 0.0237 | 0.0242 | 0.0238 | 0.0235 | 0.0240 | 0.0223 | 0.0238 | 0.0229 |
| 0.35 | 0.0278 | 0.0277 | 0.0279 | 0.0277 | 0.0276 | 0.0279 | 0.0287 | 0.0283 | 0.0270 |
| 0.4 | 0.0315 | 0.0318 | 0.0316 | 0.0319 | 0.0321 | 0.0320 | 0.0316 | 0.0327 | 0.0316 |
| 0.45 | 0.0328 | 0.0327 | 0.0331 | 0.0329 | 0.0327 | 0.0331 | 0.0315 | 0.0327 | 0.0330 |
| 0.5 | 0.0324 | 0.0318 | 0.0328 | 0.0319 | 0.0315 | 0.0312 | 0.0308 | 0.0305 | 0.0313 |
| 0.55 | 0.0359 | 0.0361 | 0.0361 | 0.0361 | 0.0360 | 0.0362 | 0.0358 | 0.0353 | 0.0358 |
| 0.6 | 0.0319 | 0.0322 | 0.0318 | 0.0323 | 0.0325 | 0.0321 | 0.0327 | 0.0368 | 0.0301 |
| 0.65 | 0.0354 | 0.0352 | 0.0355 | 0.0351 | 0.0349 | 0.0352 | 0.0378 | 0.0365 | 0.0327 |
| 0.7 | 0.0394 | 0.0391 | 0.0396 | 0.0395 | 0.0394 | 0.0396 | 0.0379 | 0.0384 | 0.0404 |
| 0.75 | 0.0376 | 0.0379 | 0.0378 | 0.0380 | 0.0380 | 0.0380 | 0.0356 | 0.0386 | 0.0373 |
| 0.8 | 0.0335 | 0.0339 | 0.0334 | 0.0334 | 0.0333 | 0.0334 | 0.0322 | 0.0309 | 0.0352 |
| 0.85 | 0.0269 | 0.0271 | 0.0271 | 0.0273 | 0.0275 | 0.0272 | 0.0294 | 0.0293 | 0.0254 |
| 0.9 | 0.0256 | 0.0256 | 0.0255 | 0.0257 | 0.0258 | 0.0257 | 0.0255 | 0.0249 | 0.0275 |
| 0.95 | 0.0367 | 0.0371 | 0.0369 | 0.0375 | 0.0381 | 0.0375 | 0.0400 | 0.0433 | 0.0384 |
| women |  |  |  |  |  |  |  |  |  |
| 0.05 | 0.0446 | 0.0443 | 0.0449 | 0.0445 | 0.0444 | 0.0446 | 0.0440 | 0.0408 | 0.0434 |
| 0.1 | 0.0558 | 0.0554 | 0.0559 | 0.0558 | 0.0557 | 0.0558 | 0.0542 | 0.0579 | 0.0558 |
| 0.15 | 0.0721 | 0.0723 | 0.0719 | 0.0721 | 0.0720 | 0.0721 | 0.0755 | 0.0702 | 0.0715 |
| 0.2 | 0.0812 | 0.0806 | 0.0814 | 0.0810 | 0.0808 | 0.0812 | 0.0830 | 0.0814 | 0.0817 |
| 0.25 | 0.0788 | 0.0785 | 0.0787 | 0.0786 | 0.0783 | 0.0788 | 0.0786 | 0.0773 | 0.0761 |
| 0.3 | 0.0808 | 0.0805 | 0.0811 | 0.0810 | 0.0807 | 0.0812 | 0.0774 | 0.0769 | 0.0789 |
| 0.35 | 0.0886 | 0.0878 | 0.0889 | 0.0884 | 0.0879 | 0.0887 | 0.0882 | 0.0854 | 0.0897 |
| 0.4 | 0.0925 | 0.0921 | 0.0927 | 0.0922 | 0.0917 | 0.0924 | 0.0880 | 0.0911 | 0.0911 |
| 0.45 | 0.0945 | 0.0947 | 0.0944 | 0.0949 | 0.0949 | 0.0950 | 0.0914 | 0.0985 | 0.0951 |
| 0.5 | 0.0996 | 0.0992 | 0.0999 | 0.0999 | 0.0998 | 0.1000 | 0.0978 | 0.1044 | 0.1013 |
| 0.55 | 0.1007 | 0.1003 | 0.1010 | 0.1002 | 0.0996 | 0.1004 | 0.0977 | 0.0974 | 0.1009 |
| 0.6 | 0.0954 | 0.0956 | 0.0952 | 0.0955 | 0.0956 | 0.0955 | 0.0927 | 0.0937 | 0.0937 |
| 0.65 | 0.1012 | 0.1012 | 0.1011 | 0.1018 | 0.1020 | 0.1017 | 0.1034 | 0.1000 | 0.1081 |
| 0.7 | 0.1022 | 0.1014 | 0.1024 | 0.1012 | 0.1005 | 0.1015 | 0.0947 | 0.0937 | 0.0979 |
| 0.75 | 0.0972 | 0.0971 | 0.0975 | 0.0976 | 0.0973 | 0.0975 | 0.0994 | 0.0950 | 0.0918 |
| 0.8 | 0.1014 | 0.1013 | 0.1000 | 0.1005 | 0.1008 | 0.1004 | 0.0988 | 0.1031 | 0.1015 |
| 0.85 | 0.0805 | 0.0802 | 0.0808 | 0.0808 | 0.0809 | 0.0808 | 0.0777 | 0.0703 | 0.0816 |
| 0.9 | 0.0708 | 0.0704 | 0.0711 | 0.0715 | 0.0716 | 0.0715 | 0.0660 | 0.0697 | 0.0698 |
| 0.95 | 0.0716 | 0.0722 | 0.0717 | 0.0727 | 0.0737 | 0.0735 | 0.0837 | 0.0786 | 0.0746 |
| Social Class V |  |  |  |  |  |  |  |  |  |
| men |  |  |  |  |  |  |  |  |  |
| 0.05 | 0.0033 | 0.0033 | 0.0033 | 0.0033 | 0.0033 | 0.0033 | 0.0033 | 0.0032 | 0.0031 |
| 0.1 | 0.0058 | 0.0058 | 0.0057 | 0.0058 | 0.0058 | 0.0057 | 0.0060 | 0.0059 | 0.0059 |
| 0.15 | 0.0155 | 0.0155 | 0.0154 | 0.0155 | 0.0156 | 0.0154 | 0.0152 | 0.0158 | 0.0157 |
| 0.2 | 0.0194 | 0.0194 | 0.0194 | 0.0194 | 0.0193 | 0.0194 | 0.0197 | 0.0191 | 0.0206 |
| 0.25 | 0.0185 | 0.0182 | 0.0187 | 0.0185 | 0.0183 | 0.0185 | 0.0183 | 0.0190 | 0.0177 |
| 0.3 | 0.0211 | 0.0209 | 0.0214 | 0.0210 | 0.0207 | 0.0212 | 0.0197 | 0.0210 | 0.0202 |
| 0.35 | 0.0256 | 0.0255 | 0.0257 | 0.0255 | 0.0254 | 0.0257 | 0.0265 | 0.0261 | 0.0249 |
| 0.4 | 0.0313 | 0.0315 | 0.0314 | 0.0317 | 0.0318 | 0.0317 | 0.0313 | 0.0324 | 0.0314 |
| 0.45 | 0.0329 | 0.0328 | 0.0332 | 0.0330 | 0.0328 | 0.0332 | 0.0316 | 0.0328 | 0.0330 |
| 0.5 | 0.0373 | 0.0366 | 0.0378 | 0.0367 | 0.0362 | 0.0361 | 0.0355 | 0.0351 | 0.0361 |
| 0.55 | 0.0377 | 0.0380 | 0.0379 | 0.0379 | 0.0378 | 0.0380 | 0.0376 | 0.0371 | 0.0376 |
| 0.6 | 0.0413 | 0.0418 | 0.0412 | 0.0419 | 0.0422 | 0.0417 | 0.0424 | 0.0477 | 0.0390 |
| 0.65 | 0.0410 | 0.0407 | 0.0411 | 0.0406 | 0.0404 | 0.0407 | 0.0437 | 0.0423 | 0.0378 |
| 0.7 | 0.0424 | 0.0421 | 0.0426 | 0.0424 | 0.0423 | 0.0425 | 0.0408 | 0.0413 | 0.0434 |
| 0.75 | 0.0446 | 0.0449 | 0.0448 | 0.0450 | 0.0451 | 0.0450 | 0.0422 | 0.0457 | 0.0442 |
| 0.8 | 0.0483 | 0.0488 | 0.0481 | 0.0480 | 0.0479 | 0.0481 | 0.0463 | 0.0444 | 0.0506 |
| 0.85 | 0.0470 | 0.0473 | 0.0474 | 0.0478 | 0.0480 | 0.0476 | 0.0513 | 0.0512 | 0.0444 |
| 0.9 | 0.0484 | 0.0484 | 0.0484 | 0.0487 | 0.0489 | 0.0486 | 0.0483 | 0.0471 | 0.0521 |
| 0.95 | 0.0689 | 0.0697 | 0.0695 | 0.0705 | 0.0716 | 0.0718 | 0.0753 | 0.0815 | 0.0723 |
| women |  |  |  |  |  |  |  |  |  |
| 0.05 | 0.0374 | 0.0371 | 0.0376 | 0.0373 | 0.0372 | 0.0374 | 0.0369 | 0.0342 | 0.0364 |
| 0.1 | 0.0532 | 0.0527 | 0.0532 | 0.0531 | 0.0530 | 0.0532 | 0.0517 | 0.0551 | 0.0531 |
| 0.15 | 0.0704 | 0.0706 | 0.0702 | 0.0704 | 0.0703 | 0.0704 | 0.0737 | 0.0685 | 0.0698 |
| 0.2 | 0.0872 | 0.0866 | 0.0873 | 0.0870 | 0.0867 | 0.0872 | 0.0891 | 0.0873 | 0.0877 |
| 0.25 | 0.0855 | 0.0852 | 0.0853 | 0.0853 | 0.0850 | 0.0855 | 0.0853 | 0.0839 | 0.0826 |
| 0.3 | 0.0891 | 0.0888 | 0.0894 | 0.0893 | 0.0890 | 0.0896 | 0.0853 | 0.0848 | 0.0870 |
| 0.35 | 0.0935 | 0.0927 | 0.0938 | 0.0933 | 0.0927 | 0.0936 | 0.0930 | 0.0901 | 0.0946 |
| 0.4 | 0.0980 | 0.0976 | 0.0982 | 0.0976 | 0.0971 | 0.0979 | 0.0932 | 0.0965 | 0.0965 |
| 0.45 | 0.1018 | 0.1020 | 0.1017 | 0.1022 | 0.1022 | 0.1023 | 0.0984 | 0.1061 | 0.1024 |
| 0.5 | 0.1034 | 0.1030 | 0.1038 | 0.1037 | 0.1036 | 0.1038 | 0.1016 | 0.1084 | 0.1052 |
| 0.55 | 0.1055 | 0.1050 | 0.1057 | 0.1049 | 0.1043 | 0.1051 | 0.1023 | 0.1019 | 0.1056 |
| 0.6 | 0.1029 | 0.1030 | 0.1027 | 0.1030 | 0.1030 | 0.1030 | 0.0999 | 0.1010 | 0.1010 |
| 0.65 | 0.1058 | 0.1058 | 0.1057 | 0.1064 | 0.1067 | 0.1064 | 0.1081 | 0.1046 | 0.1130 |
| 0.7 | 0.1099 | 0.1091 | 0.1102 | 0.1089 | 0.1082 | 0.1092 | 0.1019 | 0.1008 | 0.1053 |
| 0.75 | 0.1033 | 0.1032 | 0.1036 | 0.1037 | 0.1034 | 0.1039 | 0.1056 | 0.1010 | 0.0975 |
| 0.8 | 0.1069 | 0.1069 | 0.1055 | 0.1060 | 0.1063 | 0.1059 | 0.1042 | 0.1087 | 0.1071 |
| 0.85 | 0.0974 | 0.0970 | 0.0977 | 0.0977 | 0.0979 | 0.0977 | 0.0939 | 0.0850 | 0.0987 |
| 0.9 | 0.0875 | 0.0870 | 0.0879 | 0.0883 | 0.0885 | 0.0882 | 0.0815 | 0.0861 | 0.0862 |
| 0.95 | 0.0790 | 0.0797 | 0.0791 | 0.0801 | 0.0813 | 0.0807 | 0.0924 | 0.0867 | 0.0823 |
| Social Class VI |  |  |  |  |  |  |  |  |  |
| men |  |  |  |  |  |  |  |  |  |
| 0.05 | -0.0073 | -0.0073 | -0.0073 | -0.0073 | -0.0073 | -0.0073 | -0.0074 | -0.0070 | -0.0069 |
| 0.1 | -0.0167 | -0.0168 | -0.0165 | -0.0166 | -0.0168 | -0.0165 | -0.0173 | -0.0169 | -0.0169 |
| 0.15 | -0.0010 | -0.0010 | -0.0010 | -0.0010 | -0.0010 | -0.0010 | -0.0010 | -0.0010 | -0.0010 |
| 0.2 | 0.0027 | 0.0027 | 0.0027 | 0.0027 | 0.0027 | 0.0027 | 0.0028 | 0.0027 | 0.0029 |
| 0.25 | 0.0100 | 0.0098 | 0.0101 | 0.0099 | 0.0099 | 0.0100 | 0.0099 | 0.0102 | 0.0095 |
| 0.3 | 0.0147 | 0.0145 | 0.0149 | 0.0146 | 0.0144 | 0.0148 | 0.0137 | 0.0146 | 0.0141 |
| 0.35 | 0.0192 | 0.0192 | 0.0193 | 0.0192 | 0.0191 | 0.0193 | 0.0199 | 0.0196 | 0.0187 |
| 0.4 | 0.0265 | 0.0267 | 0.0265 | 0.0268 | 0.0269 | 0.0268 | 0.0265 | 0.0274 | 0.0265 |
| 0.45 | 0.0304 | 0.0303 | 0.0307 | 0.0305 | 0.0303 | 0.0307 | 0.0292 | 0.0303 | 0.0306 |
| 0.5 | 0.0332 | 0.0325 | 0.0336 | 0.0326 | 0.0322 | 0.0324 | 0.0315 | 0.0312 | 0.0321 |
| 0.55 | 0.0361 | 0.0363 | 0.0362 | 0.0362 | 0.0361 | 0.0363 | 0.0359 | 0.0354 | 0.0360 |
| 0.6 | 0.0413 | 0.0418 | 0.0412 | 0.0418 | 0.0422 | 0.0417 | 0.0424 | 0.0477 | 0.0390 |
| 0.65 | 0.0415 | 0.0413 | 0.0417 | 0.0412 | 0.0409 | 0.0413 | 0.0443 | 0.0428 | 0.0383 |
| 0.7 | 0.0393 | 0.0391 | 0.0395 | 0.0394 | 0.0393 | 0.0395 | 0.0379 | 0.0384 | 0.0404 |
| 0.75 | 0.0360 | 0.0362 | 0.0361 | 0.0363 | 0.0364 | 0.0363 | 0.0341 | 0.0369 | 0.0357 |
| 0.8 | 0.0434 | 0.0439 | 0.0432 | 0.0432 | 0.0431 | 0.0432 | 0.0417 | 0.0400 | 0.0455 |
| 0.85 | 0.0408 | 0.0410 | 0.0411 | 0.0414 | 0.0417 | 0.0413 | 0.0445 | 0.0444 | 0.0385 |
| 0.9 | 0.0480 | 0.0480 | 0.0480 | 0.0483 | 0.0485 | 0.0482 | 0.0479 | 0.0467 | 0.0516 |
| 0.95 | 0.0637 | 0.0644 | 0.0642 | 0.0652 | 0.0661 | 0.0655 | 0.0696 | 0.0753 | 0.0668 |
| women |  |  |  |  |  |  |  |  |  |
| 0.05 | 0.0479 | 0.0476 | 0.0482 | 0.0478 | 0.0477 | 0.0479 | 0.0473 | 0.0438 | 0.0466 |
| 0.1 | 0.0684 | 0.0679 | 0.0685 | 0.0684 | 0.0683 | 0.0685 | 0.0665 | 0.0710 | 0.0684 |
| 0.15 | 0.0909 | 0.0912 | 0.0907 | 0.0909 | 0.0908 | 0.0910 | 0.0952 | 0.0885 | 0.0902 |
| 0.2 | 0.1035 | 0.1028 | 0.1038 | 0.1034 | 0.1030 | 0.1035 | 0.1059 | 0.1038 | 0.1042 |
| 0.25 | 0.1031 | 0.1028 | 0.1030 | 0.1029 | 0.1025 | 0.1032 | 0.1029 | 0.1012 | 0.0997 |
| 0.3 | 0.1026 | 0.1023 | 0.1030 | 0.1029 | 0.1026 | 0.1032 | 0.0983 | 0.0977 | 0.1003 |
| 0.35 | 0.1049 | 0.1040 | 0.1053 | 0.1047 | 0.1040 | 0.1050 | 0.1044 | 0.1011 | 0.1062 |
| 0.4 | 0.1067 | 0.1063 | 0.1070 | 0.1064 | 0.1058 | 0.1066 | 0.1015 | 0.1051 | 0.1052 |
| 0.45 | 0.1135 | 0.1137 | 0.1134 | 0.1140 | 0.1139 | 0.1141 | 0.1097 | 0.1183 | 0.1141 |
| 0.5 | 0.1247 | 0.1242 | 0.1251 | 0.1251 | 0.1250 | 0.1252 | 0.1225 | 0.1307 | 0.1268 |
| 0.55 | 0.1256 | 0.1250 | 0.1259 | 0.1249 | 0.1242 | 0.1252 | 0.1218 | 0.1214 | 0.1258 |
| 0.6 | 0.1204 | 0.1206 | 0.1202 | 0.1206 | 0.1206 | 0.1206 | 0.1170 | 0.1183 | 0.1182 |
| 0.65 | 0.1255 | 0.1255 | 0.1254 | 0.1262 | 0.1265 | 0.1262 | 0.1282 | 0.1240 | 0.1341 |
| 0.7 | 0.1324 | 0.1314 | 0.1327 | 0.1311 | 0.1303 | 0.1315 | 0.1227 | 0.1215 | 0.1268 |
| 0.75 | 0.1270 | 0.1269 | 0.1274 | 0.1275 | 0.1271 | 0.1274 | 0.1298 | 0.1241 | 0.1199 |
| 0.8 | 0.1345 | 0.1344 | 0.1326 | 0.1333 | 0.1337 | 0.1331 | 0.1310 | 0.1367 | 0.1347 |
| 0.85 | 0.1308 | 0.1303 | 0.1312 | 0.1313 | 0.1315 | 0.1312 | 0.1262 | 0.1143 | 0.1326 |
| 0.9 | 0.0984 | 0.0979 | 0.0989 | 0.0993 | 0.0996 | 0.0993 | 0.0917 | 0.0969 | 0.0970 |
| 0.95 | 0.0980 | 0.0988 | 0.0980 | 0.0994 | 0.1008 | 0.1007 | 0.1145 | 0.1075 | 0.1020 |
| Universitary |  |  |  |  |  |  |  |  |  |
| men |  |  |  |  |  |  |  |  |  |
| 0.05 | 0.0090 | 0.0089 | 0.0090 | 0.0089 | 0.0089 | 0.0089 | 0.0090 | 0.0086 | 0.0084 |
| 0.1 | -0.0014 | -0.0014 | -0.0014 | -0.0014 | -0.0014 | -0.0014 | -0.0015 | -0.0014 | -0.0014 |
| 0.15 | -0.0183 | -0.0183 | -0.0182 | -0.0183 | -0.0184 | -0.0182 | -0.0180 | -0.0187 | -0.0185 |
| 0.2 | -0.0209 | -0.0209 | -0.0209 | -0.0209 | -0.0209 | -0.0210 | -0.0212 | -0.0206 | -0.0222 |
| 0.25 | -0.0296 | -0.0292 | -0.0300 | -0.0296 | -0.0294 | -0.0299 | -0.0294 | -0.0304 | -0.0284 |
| 0.3 | -0.0326 | -0.0322 | -0.0329 | -0.0323 | -0.0319 | -0.0327 | -0.0303 | -0.0323 | -0.0311 |
| 0.35 | -0.0335 | -0.0334 | -0.0336 | -0.0334 | -0.0333 | -0.0336 | -0.0347 | -0.0341 | -0.0326 |
| 0.4 | -0.0417 | -0.0420 | -0.0418 | -0.0422 | -0.0424 | -0.0422 | -0.0417 | -0.0432 | -0.0417 |
| 0.45 | -0.0428 | -0.0426 | -0.0432 | -0.0428 | -0.0426 | -0.0431 | -0.0411 | -0.0426 | -0.0430 |
| 0.5 | -0.0443 | -0.0435 | -0.0448 | -0.0436 | -0.0430 | -0.0431 | -0.0421 | -0.0417 | -0.0428 |
| 0.55 | -0.0508 | -0.0511 | -0.0510 | -0.0510 | -0.0508 | -0.0512 | -0.0506 | -0.0499 | -0.0506 |
| 0.6 | -0.0594 | -0.0601 | -0.0592 | -0.0602 | -0.0607 | -0.0599 | -0.0609 | -0.0686 | -0.0561 |
| 0.65 | -0.0583 | -0.0579 | -0.0585 | -0.0578 | -0.0575 | -0.0579 | -0.0622 | -0.0601 | -0.0538 |
| 0.7 | -0.0652 | -0.0648 | -0.0655 | -0.0653 | -0.0652 | -0.0654 | -0.0627 | -0.0636 | -0.0669 |
| 0.75 | -0.0667 | -0.0672 | -0.0670 | -0.0673 | -0.0674 | -0.0674 | -0.0632 | -0.0684 | -0.0661 |
| 0.8 | -0.0749 | -0.0756 | -0.0746 | -0.0745 | -0.0743 | -0.0746 | -0.0719 | -0.0689 | -0.0784 |
| 0.85 | -0.0755 | -0.0759 | -0.0760 | -0.0766 | -0.0771 | -0.0764 | -0.0824 | -0.0822 | -0.0713 |
| 0.9 | -0.0846 | -0.0846 | -0.0845 | -0.0852 | -0.0855 | -0.0850 | -0.0844 | -0.0824 | -0.0910 |
| 0.95 | -0.1105 | -0.1117 | -0.1113 | -0.1130 | -0.1147 | -0.1151 | -0.1206 | -0.1305 | -0.1158 |
| women |  |  |  |  |  |  |  |  |  |
| 0.05 | -0.0356 | -0.0353 | -0.0358 | -0.0355 | -0.0354 | -0.0356 | -0.0351 | -0.0326 | -0.0346 |
| 0.1 | -0.0602 | -0.0597 | -0.0603 | -0.0602 | -0.0601 | -0.0603 | -0.0585 | -0.0624 | -0.0602 |
| 0.15 | -0.0801 | -0.0803 | -0.0798 | -0.0800 | -0.0800 | -0.0801 | -0.0838 | -0.0779 | -0.0794 |
| 0.2 | -0.1019 | -0.1012 | -0.1021 | -0.1017 | -0.1014 | -0.1019 | -0.1042 | -0.1021 | -0.1025 |
| 0.25 | -0.1087 | -0.1084 | -0.1086 | -0.1085 | -0.1081 | -0.1088 | -0.1085 | -0.1067 | -0.1051 |
| 0.3 | -0.1159 | -0.1156 | -0.1164 | -0.1163 | -0.1159 | -0.1166 | -0.1111 | -0.1104 | -0.1133 |
| 0.35 | -0.1231 | -0.1220 | -0.1235 | -0.1228 | -0.1221 | -0.1232 | -0.1225 | -0.1186 | -0.1246 |
| 0.4 | -0.1349 | -0.1344 | -0.1352 | -0.1345 | -0.1337 | -0.1348 | -0.1284 | -0.1329 | -0.1329 |
| 0.45 | -0.1473 | -0.1476 | -0.1472 | -0.1480 | -0.1479 | -0.1481 | -0.1424 | -0.1536 | -0.1482 |
| 0.5 | -0.1514 | -0.1507 | -0.1519 | -0.1519 | -0.1517 | -0.1520 | -0.1487 | -0.1586 | -0.1540 |
| 0.55 | -0.1661 | -0.1654 | -0.1665 | -0.1652 | -0.1643 | -0.1656 | -0.1611 | -0.1605 | -0.1664 |
| 0.6 | -0.1697 | -0.1700 | -0.1694 | -0.1699 | -0.1700 | -0.1700 | -0.1649 | -0.1667 | -0.1666 |
| 0.65 | -0.1708 | -0.1709 | -0.1707 | -0.1719 | -0.1723 | -0.1718 | -0.1746 | -0.1689 | -0.1826 |
| 0.7 | -0.1777 | -0.1763 | -0.1781 | -0.1759 | -0.1748 | -0.1764 | -0.1647 | -0.1630 | -0.1702 |
| 0.75 | -0.1790 | -0.1789 | -0.1795 | -0.1797 | -0.1792 | -0.1793 | -0.1830 | -0.1749 | -0.1690 |
| 0.8 | -0.1946 | -0.1945 | -0.1920 | -0.1930 | -0.1935 | -0.1927 | -0.1896 | -0.1979 | -0.1949 |
| 0.85 | -0.1995 | -0.1988 | -0.2002 | -0.2002 | -0.2005 | -0.2001 | -0.1925 | -0.1742 | -0.2022 |
| 0.9 | -0.1822 | -0.1811 | -0.1830 | -0.1838 | -0.1843 | -0.1838 | -0.1698 | -0.1794 | -0.1795 |
| 0.95 | -0.1685 | -0.1699 | -0.1686 | -0.1709 | -0.1734 | -0.1731 | -0.1970 | -0.1849 | -0.1755 |
